# Associations of common breast cancer susceptibility alleles with risk of breast cancer subtypes in *BRCA1* and *BRCA2* mutation carriers

**DOI:** 10.1186/s13058-014-0492-9

**Published:** 2014-12-31

**Authors:** Karoline B Kuchenbaecker, Susan L Neuhausen, Mark Robson, Daniel Barrowdale, Lesley McGuffog, Anna Marie Mulligan, Irene L Andrulis, Amanda B Spurdle, Marjanka K Schmidt, Rita K Schmutzler, Christoph Engel, Barbara Wappenschmidt, Heli Nevanlinna, Mads Thomassen, Melissa Southey, Paolo Radice, Susan J Ramus, Susan M Domchek, Katherine L Nathanson, Andrew Lee, Sue Healey, Robert L Nussbaum, Timothy R Rebbeck, Banu K Arun, Paul James, Beth Y Karlan, Jenny Lester, Ilana Cass, Breast Cancer Family Registry, Mary Beth Terry, Mary B Daly, David E Goldgar, Saundra S Buys, Ramunas Janavicius, Laima Tihomirova, Nadine Tung, Cecilia M Dorfling, Elizabeth J van Rensburg, Linda Steele, Thomas v O Hansen, Bent Ejlertsen, Anne-Marie Gerdes, Finn C Nielsen, Joe Dennis, Julie Cunningham, Steven Hart, Susan Slager, Ana Osorio, Javier Benitez, Mercedes Duran, Jeffrey N Weitzel, Isaac Tafur, Mary Hander, Paolo Peterlongo, Siranoush Manoukian, Bernard Peissel, Gaia Roversi, Giulietta Scuvera, Bernardo Bonanni, Paolo Mariani, Sara Volorio, Riccardo Dolcetti, Liliana Varesco, Laura Papi, Maria Grazia Tibiletti, Giuseppe Giannini, Florentia Fostira, Irene Konstantopoulou, Judy Garber, Ute Hamann, Alan Donaldson, Carole Brewer, Claire Foo, D Gareth Evans, Debra Frost, Diana Eccles, Fiona Douglas, Angela Brady, Jackie Cook, Marc Tischkowitz, Julian Adlard, Julian Barwell, Kai-ren Ong, Lisa Walker, Louise Izatt, Lucy E Side, M John Kennedy, Mark T Rogers, Mary E Porteous, Patrick J Morrison, Radka Platte, Ros Eeles, Rosemarie Davidson, Shirley Hodgson, Steve Ellis, Andrew K Godwin, Kerstin Rhiem, Alfons Meindl, Nina Ditsch, Norbert Arnold, Hansjoerg Plendl, Dieter Niederacher, Christian Sutter, Doris Steinemann, Nadja Bogdanova-Markov, Karin Kast, Raymonda Varon-Mateeva, Shan Wang-Gohrke, Andrea Gehrig, Birgid Markiefka, Bruno Buecher, Cédrick Lefol, Dominique Stoppa-Lyonnet, Etienne Rouleau, Fabienne Prieur, Francesca Damiola, Laure Barjhoux, Laurence Faivre, Michel Longy, Nicolas Sevenet, Olga M Sinilnikova, Sylvie Mazoyer, Valérie Bonadona, Virginie Caux-Moncoutier, Claudine Isaacs, Tom Van Maerken, Kathleen Claes, Marion Piedmonte, Lesley Andrews, John Hays, Gustavo C Rodriguez, Trinidad Caldes, Miguel de la Hoya, Sofia Khan, Frans BL Hogervorst, Cora M Aalfs, JL de Lange, Hanne EJ Meijers-Heijboer, Annemarie H van der Hout, Juul T Wijnen, KEP van Roozendaal, Arjen R Mensenkamp, Ans MW van den Ouweland, Carolien HM van Deurzen, Rob B van der Luijt, Edith Olah, Orland Diez, Conxi Lazaro, Ignacio Blanco, Alex Teulé, Mireia Menendez, Anna Jakubowska, Jan Lubinski, Cezary Cybulski, Jacek Gronwald, Katarzyna Jaworska-Bieniek, Katarzyna Durda, Adalgeir Arason, Christine Maugard, Penny Soucy, Marco Montagna, Simona Agata, Manuel R Teixeira, Curtis Olswold, Noralane Lindor, Vernon S Pankratz, Emily Hallberg, Xianshu Wang, Csilla I Szabo, Joseph Vijai, Lauren Jacobs, Marina Corines, Anne Lincoln, Andreas Berger, Anneliese Fink-Retter, Christian F Singer, Christine Rappaport, Daphne Gschwantler Kaulich, Georg Pfeiler, Muy-Kheng Tea, Catherine M Phelan, Phuong L Mai, Mark H Greene, Gad Rennert, Evgeny N Imyanitov, Gord Glendon, Amanda Ewart Toland, Anders Bojesen, Inge Sokilde Pedersen, Uffe Birk Jensen, Maria A Caligo, Eitan Friedman, Raanan Berger, Yael Laitman, Johanna Rantala, Brita Arver, Niklas Loman, Ake Borg, Hans Ehrencrona, Olufunmilayo I Olopade, Jacques Simard, Douglas F Easton, Georgia Chenevix-Trench, Kenneth Offit, Fergus J Couch, Antonis C Antoniou, EMBRACE Study, GEMO Study Collaborators, HEBON, KConFab Investigators

**Affiliations:** 1 Centre for Cancer Genetic Epidemiology, Department of Public Health and Primary Care, University of Cambridge, Cambridge, UK; 2 Department of Population Sciences, Beckman Research Institute of City of Hope, Duarte, CA USA; 3 Clinical Genetics Research Laboratory, Memorial Sloan-Kettering Cancer Center, New York, NY USA; 4 Department of Laboratory Medicine and Pathobiology, University of Toronto, Toronto, ON Canada; 5 Laboratory Medicine Program, University Health Network, Toronto, ON Canada; 6 Lunenfeld-Tanenbaum Research Institute of Mount Sinai Hospital, Toronto, ON Canada; 7 Departments of Molecular Genetics and Laboratory Medicine and Pathobiology, University of Toronto, Toronto, ON Canada; 8 Department of Genetics and Computational Biology, QIMR Berghofer Medical Research Institute, Brisbane, Australia; 9 Division of Psychosocial Research and Epidemiology, Netherlands Cancer Institute, Amsterdam, the Netherlands; 10 Center for Hereditary Breast and Ovarian Cancer, Medical Faculty, University Hospital Cologne, Cologne, Germany; 11 Center for Integrated Oncology (CIO), Medical Faculty, University Hospital Cologne, Cologne, Germany; 12 Center for Molecular Medicine Cologne (CMMC), University of Cologne, on behalf of the German Consortium of Hereditary Breast and Ovarian Cancer (GC-HBOC), Cologne, Germany; 13 Institute for Medical Informatics, Statistics and Epidemiology University of Leipzig, Leipzig, Germany; 14 Center for Molecular Medicine Cologne (CMMC), University of Cologne, Cologne, Germany; 15 Department of Obstetrics and Gynecology, University of Helsinki and Helsinki University Central Hospital, Helsinki, HUS Finland; 16 Department of Clinical Genetics, Odense University Hospital, Odense C, Denmark; 17 Department of Pathology, Genetic Epidemiology Laboratory, University of Melbourne, Parkville, Australia; 18 Department of Preventive and Predictive Medicine, Unit of Molecular Bases of Genetic Risk and Genetic Testing, Fondazione IRCCS Istituto Nazionale Tumori (INT), Milan, Italy; 19 Department of Preventive Medicine, Keck School of Medicine, University of Southern California, Los Angeles, CA USA; 20 Department of Medicine, Abramson Cancer Center, Perelman School of Medicine, University of Pennsylvania, Philadelphia, PA USA; 21 Department of Genetics & Computational Biology, Queensland Institute of Medical Research, Herston, Australia; 22 Department of Medicine and Institute for Human Genetics, University of California, San Francisco, CA USA; 23 Abramson Cancer Center and Center for Clinical Epidemiology and Biostatistics, Perelman School of Medicine, University of Pennsylvania, Philadelphia, PA USA; 24 University of Texas MD Anderson Cancer Center, Houston, TX USA; 25 Familial Cancer Centre, Peter MacCallum Cancer Centre, Melbourne, Australia; 26 Sir Peter MacCallum Department of Oncology, University of Melbourne, Parkville, Australia; 27 Women’s Cancer Program at the Samuel Oschin Comprehensive Cancer Institute, Cedars-Sinai Medical Center, Los Angeles, CA USA; 28 Department of Epidemiology, Cancer Prevention Institute of California, Fremont, CA USA; 29 Department of Epidemiology, Columbia University, New York, NY USA; 30 Fox Chase Cancer Center, Philadelphia, PA USA; 31 Department of Dermatology, University of Utah School of Medicine, Salt Lake City, UT USA; 32 Department of Oncological Sciences, Huntsman Cancer Institute, University of Utah School of Medicine, Salt Lake City, UT USA; 33 Department of Molecular and Regenerative Medicine, Vilnius University Hospital Santariskiu Clinics, Hematology, Oncology and Transfusion Medicine Center, Vilnius, Lithuania; 34 State Research Institute Centre for Innovative Medicine, Vilnius, Lithuania; 35 Latvian Biomedical Research and Study Centre, Riga, Latvia; 36 Department of Medical Oncology, Beth Israel Deaconess Medical Center, Boston, MA USA; 37 Department of Genetics, University of Pretoria, Pretoria, South Africa; 38 Center for Genomic Medicine, Rigshospitalet, Copenhagen University Hospital, Copenhagen, Denmark; 39 Department of Oncology, Rigshospitalet, Copenhagen University Hospital, Copenhagen, Denmark; 40 Department of Clinical Genetics, Rigshospitalet, Copenhagen University Hospital, Copenhagen, Denmark; 41 Department of Laboratory Medicine and Pathology, Mayo Clinic, Rochester, MN USA; 42 Department of Health Sciences Research, Mayo Clinic, Rochester, MN USA; 43 Human Genetics Group, Spanish National Cancer Centre (CNIO), and Biomedical Network on Rare Diseases (CIBERER), Madrid, Spain; 44 Human Genetics Group and Genotyping Unit, Spanish National Cancer Centre (CNIO), and Biomedical Network on Rare Diseases (CIBERER), Madrid, Spain; 45 Institute of Biology and Molecular Genetics, Universidad de Valladolid (IBGM-UVA), Valladolid, Spain; 46 Clinical Cancer Genetics, City of Hope (for the City of Hope Clinical Cancer Genetics Community Research Network), Duarte, CA USA; 47 Covenant Health Joe Arrington Cancer Research Center, care of City of Hope Clinical Cancer Genetics Community Research Network, Duarte, CA USA; 48 Kootenai Cancer Center, care of City of Hope Clinical Cancer Genetics Community Research Network, Duarte, CA USA; 49 IFOM, Fondazione Istituto FIRC di Oncologia Molecolare, Milan, Italy; 50 Unit of Medical Genetics, Department of Preventive and Predictive Medicine, Fondazione IRCCS Istituto Nazionale Tumori (INT), Milan, Italy; 51 Division of Cancer Prevention and Genetics, European Institute of Oncology, Milan, Italy; 52 IFOM, Fondazione Istituto FIRC di Oncologia Molecolare and Cogentech Cancer Genetic Test Laboratory, Milan, Italy; 53 Cancer Bioimmunotherapy Unit, Centro di Riferimento Oncologico, IRCCS, Aviano, PN Italy; 54 Unit of Hereditary Cancer, IRCCS AOU San Martino, IST Istituto Nazionale per la Ricerca sul Cancro, Genoa, Italy; 55 Unit of Medical Genetics, Department of Biomedical, Experimental and Clinical Sciences, University of Florence, Florence, Italy; 56 Ospedale di Circolo e Fondazione Macchi Polo Universitario, Varese, Italy; 57 Department of Molecular Medicine, University La Sapienza, Rome, Italy; 58 Molecular Diagnostics Laboratory, INRASTES, National Centre for Scientific Research “Demokritos”, Aghia Paraskevi Attikis, Athens, Greece; 59 Dana-Farber Cancer Institute, Boston, MA USA; 60 Molecular Genetics of Breast Cancer, Deutsches Krebsforschungszentrum (DKFZ), Heidelberg, Germany; 61 Clinical Genetics Department, St Michael’s Hospital, Bristol, UK; 62 Department of Clinical Genetics, Royal Devon and Exeter Hospital, Exeter, UK; 63 Cheshire and Merseyside Clinical Genetics Service, Liverpool Women’s NHS Foundation Trust, Liverpool, UK; 64 Genetic Medicine, Manchester Academic Health Sciences Centre, Central Manchester University Hospitals NHS Foundation Trust, Manchester, UK; 65 University of Southampton Faculty of Medicine, Southampton University Hospitals NHS Trust, Southampton, UK; 66 Institute of Genetic Medicine, Centre for Life, Newcastle Upon Tyne Hospitals NHS Trust, Newcastle upon Tyne, UK; 67 North West Thames Regional Genetics Service, Kennedy-Galton Centre, Harrow, UK; 68 Sheffield Clinical Genetics Service, Sheffield Children’s Hospital, Sheffield, UK; 69 Department of Clinical Genetics, East Anglian Regional Genetics Service, Addenbrookes Hospital, Cambridge, UK; 70 Yorkshire Regional Genetics Service, Leeds, UK; 71 Leicestershire Clinical Genetics Service, University Hospitals of Leicester NHS Trust, Leicester, UK; 72 West Midlands Regional Genetics Service, Birmingham Women’s Hospital Healthcare NHS Trust, Edgbaston, Birmingham UK; 73 Oxford Regional Genetics Service, Churchill Hospital, Oxford, UK; 74 Clinical Genetics, Guy’s and St Thomas’ NHS Foundation Trust, London, UK; 75 North East Thames Regional Genetics Service, Great Ormond Street Hospital for Children NHS Trust, London, UK; 76 Academic Unit of Clinical and Molecular Oncology, Trinity College Dublin and St James’s Hospital, Dublin, Ireland; 77 All Wales Medical Genetics Services, University Hospital of Wales, Cardiff, UK; 78 South East of Scotland Regional Genetics Service, Western General Hospital, Edinburgh, UK; 79 Department of Medical Genetics, Belfast Health and Social Care Trust, Centre for Cancer Research & Cell Biology, Queen’s University Belfast, Belfast, UK; 80 Oncogenetics Team, The Institute of Cancer Research and Royal Marsden NHS Foundation Trust, London, UK; 81 Ferguson-Smith Centre for Clinical Genetics, Yorkhill Hospitals, Glasgow, UK; 82 Medical Genetics Unit, St George’s, University of London, London, UK; 83 Department of Pathology and Laboratory Medicine, University of Kansas Medical Center, Kansas City, KS USA; 84 Department of Gynaecology and Obstetrics, Division of Tumor Genetics, Klinikum rechts der Isar, Technical University of Munich, Munich, Germany; 85 Department of Gynaecology and Obstetrics, University Munich, Munich, Germany; 86 University Hospital of Schleswig-Holstein/University Kiel, Kiel, Germany; 87 Institute of Human Genetics, University Hospital of Schleswig-Holstein, University Kiel, Kiel, Germany; 88 University Düsseldorf, Dusseldorf, Germany; 89 University Heidelberg, Heidelberg, Germany; 90 Hannover Medical School, Hannover, Germany; 91 Institute of Human Genetics, Münster, Germany; 92 University Dresden, Dresden, Germany; 93 Institute of Medical Genetics and Human Genetics, Charité, Berlin Germany; 94 Department of Gynaecology and Obstetrics, University Hospital Ulm, Ulm, Germany; 95 Institute of Human Genetics, University Wurzburg, Wurzburg, Germany; 96 Department of Tumour Biology, Institut Curie, Paris, France; 97 Institut Curie, INSERM U830, Paris, France; 98 Université Paris Descartes, Sorbonne Paris Cité, Paris France; 99 Laboratoire d’Oncogénétique, Hôpital René Huguenin, Institut Curie, Saint-Cloud, France; 100 Service de Génétique Clinique Chromosomique et Moléculaire, Centre Hospitalier Universitaire de St Etienne, St Etienne, France; 101 INSERM U1052, CNRS UMR5286, Université Lyon, Centre de Recherche en Cancérologie de Lyon, Lyon, France; 102 GEMO Study: National Cancer Genetics Network, UNICANCER Genetic Group, Paris, France; 103 Centre de Génétique, CHU Dijon, Université de Bourgogne, Dijon, France; 104 Centre Georges François Leclerc, Dijon, France; 105 Cancer Genetics Unit, INSERM U916, Institut Bergonié, Université de Bordeaux, Bordeaux, France; 106 Unité Mixte de Génétique Constitutionnelle des Cancers Fréquents, Hospices Civils de Lyon, Centre Léon Bérard, Lyon, France; 107 Université Lyon 1, CNRS UMR5558, Lyon, France; 108 Unité de Prévention et d’Epidémiologie Génétique, Centre Léon Bérard, Lyon, France; 109 Lombardi Comprehensive Cancer Center, Georgetown University, Washington, DC USA; 110 Center for Medical Genetics, Ghent University, Ghent, Belgium; 111 Gynecologic Oncology Group Statistical and Data Center, Roswell Park Cancer Institute, Buffalo, NY USA; 112 ANZGOG Australia, New Zealand Gynaecological Oncology Group, Prince of Wales Hospital, Randwick, Australia; 113 The Ohio State University, Columbus Cancer Council, Columbus, OH USA; 114 Division of Gynecologic Oncology, NorthShore University HealthSystem, Evanston, IL USA; 115 Molecular Oncology Laboratory, Instituto de Investigación Sanitaria del Hospital Clinico San Carlos (IdISSC), Madrid, Spain; 116 Family Cancer Clinic, Netherlands Cancer Institute, Amsterdam, the Netherlands; 117 Department of Clinical Genetics, Academic Medical Center, Amsterdam, The Netherlands; 118 Department of Epidemiology, Netherlands Cancer Institute, Amsterdam, The Netherlands; 119 Department of Clinical Genetics, VU University Medical Centre, Amsterdam, The Netherlands; 120 Department of Genetics, University Medical Center, Groningen University, Groningen, The Netherlands; 121 Department of Human Genetics and Department of Clinical Genetics, Leiden University Medical Center, Leiden, The Netherlands; 122 Department of Clinical Genetics, Maastricht University Medical Center, Maastricht, The Netherlands; 123 Department of Human Genetics, Radboud University Nijmegen Medical Centre, Nijmegen, The Netherlands; 124 Department of Clinical Genetics, Erasmus University Medical Center, Rotterdam, The Netherlands; 125 Department of Pathology, Family Cancer Clinic, Erasmus University Medical Center, Rotterdam, The Netherlands; 126 Department of Medical Genetics, University Medical Center Utrecht, Utrecht, The Netherlands; 127 The Hereditary Breast and Ovarian Cancer Research Group Netherlands (HEBON), coordinating center: Netherlands Cancer Institute, Amsterdam, The Netherlands; 128 Department of Molecular Genetics, National Institute of Oncology, Budapest, Hungary; 129 Oncogenetics Group, University Hospital Vall d’Hebron, Vall d’Hebron Institute of Oncology (VHIO), Vall d’Hebron Research Institute (VHIR), Universitat Autònoma de Barcelona, Barcelona, Spain; 130 Molecular Diagnostic Unit, Hereditary Cancer Program, IDIBELL-Catalan Institute of Oncology, Barcelona, Spain; 131 Genetic Counseling Unit, Hereditary Cancer Program, IDIBELL-Catalan Institute of Oncology, Barcelona, Spain; 132 Department of Genetics and Pathology, Pomeranian Medical University, Szczecin, Poland; 133 Department of Pathology, Landspitali University Hospital and BMC, Faculty of Medicine, University of Iceland, Reykjavik, Iceland; 134 Laboratoire de diagnostic génétique et Service d’Onco-hématologie, Hopitaux Universitaire de Strasbourg, CHRU Nouvel Hôpital Civil, Strasbourg, France; 135 Centre Hospitalier Universitaire de Québec Research Center and Laval University, Quebec City, QC Canada; 136 Immunology and Molecular Oncology Unit, Istituto Oncologico Veneto IOV, IRCCS, Padua, Italy; 137 Department of Genetics, Portuguese Oncology Institute, Porto, Portugal; 138 kConFab: Kathleen Cuningham Consortium for Research into Familial Breast Cancer, Peter MacCallum Cancer Center, Melbourne, Australia; 139 Health Sciences Research, Mayo Clinic, Scottsdale, AZ USA; 140 Department of Laboratory Medicine and Pathology, Mayo Clinic, Rochester, MN USA; 141 National Human Genome Research Institute, National Institutes of Health, Bethesda, MD USA; 142 Clinical Genetics Service, Department of Medicine, Memorial Sloan-Kettering Cancer Center, New York, NY USA; 143 Department of Obstetrics and Gynecology and Comprehensive Cancer Center, , Medical University of Vienna, Vienna, Austria; 144 Department of Cancer Epidemiology, Moffitt Cancer Center, Tampa, FL USA; 145 Clinical Genetics Branch, Division of Cancer Epidemiology and Genetics, National Cancer Institute, National Institutes of Health, Rockville, MD USA; 146 Clalit National Israeli Cancer Control Center and Department of Community Medicine and Epidemiology, Carmel Medical Center and B Rappaport Faculty of Medicine, Haifa, Israel; 147 NN Petrov Institute of Oncology, St Petersburg, Russia; 148 Ontario Cancer Genetics Network: Samuel Lunenfeld Research Institute, Mount Sinai Hospital, Cancer Care Ontario, Toronto, ON Canada; 149 Divison of Human Cancer Genetics, Departments of Internal Medicine and Molecular Virology, Immunology and Medical Genetics, Comprehensive Cancer Center, The Ohio State University, Columbus, OH USA; 150 Department of Clinical Genetics, Vejle Hospital, Vejle, Denmark; 151 Section of Molecular Diagnostics, Department of Biochemistry, Aalborg University Hospital, Aalborg, Denmark; 152 Department of Clinical Genetics, Aarhus University Hospital, Aarhus N, Denmark; 153 Section of Genetic Oncology, Department of Laboratory Medicine, University and University Hospital of Pisa, Pisa, Italy; 154 Sheba Medical Center, Tel Aviv, Israel; 155 Department of Clinical Genetics, Karolinska University Hospital, Stockholm, Sweden; 156 Department of Oncology, Karolinska University Hospital, Stockholm, Sweden; 157 Department of Oncology, Lund University Hospital, Lund, Sweden; 158 Department of Oncology, Lund University, Lund, Sweden; 161 Center for Clinical Cancer Genetics and Global Health, University of Chicago Medical Center, Chicago, IL USA; 162 Department of Clinical Genetics, Lund University Hospital, Lund, Sweden; 163 5841 South Maryland Avenue, Chicago, MC, 2115 IL USA

## Abstract

**Introduction:**

More than 70 common alleles are known to be involved in breast cancer (BC) susceptibility, and several exhibit significant heterogeneity in their associations with different BC subtypes. Although there are differences in the association patterns between *BRCA1* and *BRCA2* mutation carriers and the general population for several loci, no study has comprehensively evaluated the associations of all known BC susceptibility alleles with risk of BC subtypes in *BRCA1* and *BRCA2* carriers.

**Methods:**

We used data from 15,252 *BRCA1* and 8,211 *BRCA2* carriers to analyze the associations between approximately 200,000 genetic variants on the iCOGS array and risk of BC subtypes defined by estrogen receptor (ER), progesterone receptor (PR), human epidermal growth factor receptor 2 (HER2) and triple-negative- (TN) status; morphologic subtypes; histological grade; and nodal involvement.

**Results:**

The estimated BC hazard ratios (HRs) for the 74 known BC alleles in *BRCA1* carriers exhibited moderate correlations with the corresponding odds ratios from the general population. However, their associations with ER-positive BC in *BRCA1* carriers were more consistent with the ER-positive associations in the general population (intraclass correlation (ICC) = 0.61, 95% confidence interval (CI): 0.45 to 0.74), and the same was true when considering ER-negative associations in both groups (ICC = 0.59, 95% CI: 0.42 to 0.72). Similarly, there was strong correlation between the ER-positive associations for *BRCA1* and *BRCA2* carriers (ICC = 0.67, 95% CI: 0.52 to 0.78), whereas ER-positive associations in any one of the groups were generally inconsistent with ER-negative associations in any of the others. After stratifying by ER status in mutation carriers, additional significant associations were observed. Several previously unreported variants exhibited associations at *P* <10^−6^ in the analyses by PR status, HER2 status, TN phenotype, morphologic subtypes, histological grade and nodal involvement.

**Conclusions:**

Differences in associations of common BC susceptibility alleles between *BRCA1* and *BRCA2* carriers and the general population are explained to a large extent by differences in the prevalence of ER-positive and ER-negative tumors. Estimates of the risks associated with these variants based on population-based studies are likely to be applicable to mutation carriers after taking ER status into account, which has implications for risk prediction.

**Electronic supplementary material:**

The online version of this article (doi:10.1186/s13058-014-0492-9) contains supplementary material, which is available to authorized users.

## Introduction

Women who carry pathogenic mutations in *BRCA1* or *BRCA2* have markedly increased risks of developing breast cancer. The distributions of breast cancer tumor characteristics differ between *BRCA1* mutation carriers, *BRCA2* mutation carriers and those arising in the general population. The majority of breast tumors arising in *BRCA1* carriers show low or absent expression of estrogen receptor (ER) [1]-[3], whereas the majority of *BRCA2*-associated tumors are ER-positive [1],[4],[5].

Many common breast cancer susceptibility alleles identified through population-based genome-wide association studies (GWASs) have also been associated with breast cancer risk in *BRCA1* and *BRCA2* carriers [6],[7]. Several of these variants are specifically associated with the ER status of the breast cancer in the general population [8],[9]. Among the single-nucleotide polymorphisms (SNPs) that have been evaluated in mutation carriers so far, the variants found to be associated with breast cancer risk for *BRCA1* carriers largely overlap with loci for which stronger associations with ER-negative breast cancer have been reported in the general population [8]-[12]. An important question for risk modelling and prevention studies is whether the effects of common variants on breast cancer risk in mutation carriers are mediated through a generic influence on the development of particular hormone receptor subtypes of breast cancer or through epistatic interaction with the *BRCA1/2* mutation itself.

Previous studies by the Consortium of Investigators of Modifiers of BRCA1/2 (CIMBA) described the impact of 29 breast cancer susceptibility variants from non-hereditary breast cancer studies on ER-positive and ER-negative breast cancer risk in *BRCA1* and *BRCA2* carriers [6],[7],[13]-[15]. These analyses demonstrated that, despite the lack of an association between some susceptibility variants and overall breast cancer risk for *BRCA1* or *BRCA2* carriers, residual associations exist with specific disease subtypes. In addition, the ER-specific associations in *BRCA1* and *BRCA2* carriers were mainly in the same direction and of a magnitude similar to the associations observed with breast cancer stratified by ER expression status in the general population. However, these studies were conducted on smaller numbers of mutation carriers than currently available and evaluated only a subset of the currently known breast cancer susceptibility alleles for their associations with ER-specific subtypes in carriers. Recently, 45 additional SNPs have been found to be associated with breast cancer risk in the general population [8]-[10],[16]. Eighteen of these SNPs showed evidence of association with ER-positive breast cancer, but not with ER-negative breast cancer, and four loci (1q32.1 *LGR6*, 2p24.1, 16q12 and 20q11) were associated only with ER-negative breast cancer in the general population. These 45 newly discovered loci have not yet been evaluated for their associations with breast cancer risk for mutation carriers.

In the present study, we assessed the disease subtype-specific associations of all 74 previously reported breast cancer susceptibility variants in 15,252 *BRCA1* and 8,211 *BRCA2* carriers. We evaluated whether differences in associations of known breast cancer susceptibility variants between *BRCA1* carriers, *BRCA2* carriers and the general population are mediated by tumor ER status in mutation carriers. We also analyzed the associations of about 200,000 variants on the iCOGS genotyping array with subtype-specific breast cancer risk in carriers in an attempt to uncover previously unreported subtype-specific associations in women with *BRCA1* and *BRCA2* mutations. In addition to ER and progesterone receptor (PR) status, we report, for the first time to our knowledge, associations by HER2 status and with triple-negative disease (TN, referring to ER-, PR- and human epidermal growth factor receptor 2 (HER2)-negative), and we also describe associations with clinical features such as “ductal, no specified subtype” (hereafter referred to as *ductal*) and lobular morphologic subtypes, nodal status and histological grade.

## Methods

### Study subjects

Data were obtained from 47 studies in 27 different countries in CIMBA [17]. Eligible study subjects were women who carry pathogenic mutations in *BRCA1* or *BRCA2*. The majority were recruited through cancer genetics clinics and enrolled into national or regional studies. Written informed consent was obtained from all subjects. Each of the host institutions recruited under ethically approved protocols. A list of the local institutional review boards that provided ethical approval for this study is given in Additional file 1: Table S1. Eligibility was restricted to mutation carriers who were 18 years of age or older at recruitment. Data collected included year of birth, age at cancer diagnosis, personal history of bilateral prophylactic mastectomy and/or bilateral salpingo-oophorectomy, mutation description, tumor pathology and ethnicity.

### Tumor pathology data

Breast tumor pathology data were gathered from a range of sources, specifically patient pathology reports, pathology review data, tumor registry records and tissue microarray results. These included information on ER, PR and HER2 status; morphologic subtype; lymph node involvement; and histological grade. For ER, PR and HER2, status was classified as negative or positive, with supplementary immunohistochemistry scoring or biochemical data and methodology provided when available. The vast majority of centers employed a cutoff of either ≥10% or ≥1% of tumor nuclei staining positive to define ER and PR positivity. Additional file 1: Table S2 lists the subtype definitions used by each study, which were not centrally reclassified, owing to the low proportion of records with supporting staining data. Similarly, HER2 status was determined using immunohistochemistry to detect strong complete membrane staining (with a score of 3+ considered positive) and/or *in situ* hybridization to detect HER2 gene amplification. To ensure consistency across studies, when information on the cells stained was available, we used the same cutoff to define ER-, PR- and HER2-positive tumors. The cutoffs used for the small number of cases where composite scoring methods based on the proportion and intensity of staining were available (Allred score, immunoreactive Remmele score) are given in Additional file 1: Table S2. Consistency checks were performed to validate receptor data against supplementary scoring information, if provided. Each cancer was assigned to a morphologic subgroup (ductal, lobular, medullary, other), which we confirmed using the World Health Organization International Classification of Diseases for Oncology (ICD-O) code for the classification of tumor type when sufficient information was provided [18]. Lymph node status, along with the number of nodes showing metastatic carcinoma, was provided when available. Histologic grade was assigned as grade 1, 2 or 3 by local pathologists who used a modified Scarff-Bloom-Richardson malignancy grading system.

### Genotyping and quality control

Genotyping was carried out using the iCOGS custom array. The array development and details of the genotyping and quality control for the CIMBA samples are described in detail elsewhere [6],[7]. Briefly, genotyping for *BRCA2* carriers was conducted at McGill University and Génome Québec Innovation Centre (Canada) and for *BRCA1* carriers at the Mayo Clinic (USA). SNPs were excluded if they were located on the Y chromosome, if they were monomorphic, if they deviated significantly from Hardy-Weinberg equilibrium (*P* <10^−7^) or if they had call rates <95%. Samples were excluded if they had a call rate <95%, if they were of non-European ancestry or if they demonstrated extreme heterozygosity. After quality control, we had 200,720 SNPs available for analysis in 15,252 *BRCA1* samples and 200,908 SNPs available for analysis in 8,211 *BRCA2* samples.

### Statistical methods

We evaluated the associations of each genotype with risks of developing breast cancer or breast cancer subtypes defined by the tumor characteristics or morphology. The analyses were carried out within a survival analysis framework. Individuals were censored at the first of the following events: breast cancer diagnosis, ovarian cancer diagnosis, bilateral mastectomy or age at last follow-up. In order to account for non-random ascertainment of mutation carriers with respect to their disease phenotype, we used a retrospective likelihood approach that models the probability of observing the genotypes conditional on the disease phenotype [19],[20]. It was assumed that the cancer incidence depends on the underlying SNP genotype through a Cox proportional hazards model:

$$\lambda_{i}\left(t_{i}\right)=\lambda_{0}\left(t_{i}\right)\exp\left(\beta z_{i}\right)\text{,}$$

where λ_0_(*t*_i_) is the baseline incidence and β is the logarithm of the per-allele hazard ratio (HR, under a multiplicative model). The association with overall breast cancer risk was evaluated by testing the hypothesis that β = 0 [20].

We evaluated the associations with the groups of each subtype class (for example, ER-positive and ER-negative), using an extension of the retrospective likelihood approach to model the simultaneous effect of each SNP on more than one tumor subtype [15]. Briefly, this involves modeling the conditional likelihood of the observed SNP genotypes and tumor subtypes, given the disease phenotypes. Within this framework, it is possible to estimate simultaneously the HRs for each tumor subtype and test for heterogeneity in the associations [15]. To maximize the available information, genotyped mutation carriers that were missing information on tumor characteristics were included in the analysis, and their disease subtype was assumed to be missing at random. In order to account for non-independence among relatives, a robust variance estimation approach was used [20]. Further details of the methods for evaluating the associations with overall breast cancer [20] and tumor subtypes have been described elsewhere [15]. We carried out association analyses by subtype for the following breast cancer characteristics: ER-positive and ER-negative, PR-positive and PR-negative, HER2-positive and HER2-negative, TN breast cancer (that is, negative for ER, PR and HER2) and non-TN (that is, tumor positive for at least one of the three receptors), ductal morphologic subtype, lobular morphologic subtype, nodal involvement (no involved lymph nodes and at least one involved lymph node) and histological grade (high grade (grade 3) and non-high grade (grades 1 and 2)). Only samples with complete information on ER, PR and HER2 expression were included in the analysis for TN as well as non-TN breast cancer. The SNP associations by tumor morphologic subtype were evaluated by comparing ductal tumors to all others and, in a separate analysis, lobular tumors to all others. We are not reporting association analyses for risk of medullary morphologic subtype, owing to sparse data as well as the difficulties in diagnosing medullary breast tumors reliably [21],[22]. All analyses were stratified by country of residence. The United States and Canada strata were further subdivided by reported Ashkenazi Jewish ancestry. For subtypes with small groups, strata of geographically close countries were combined to provide sufficiently large groups for estimation. All analyses used calendar year– and cohort-specific cancer incidences for *BRCA1* and *BRCA2*. SNPs with minor allele frequencies <3% were excluded. The retrospective likelihood was modeled using custom-written functions implemented in the pedigree analysis software MENDEL [23].

When evaluating whether the known breast cancer susceptibility loci identified through population based studies also modify breast cancer risk in mutation carriers, a significance threshold of *P* <0.05 was used because of the strong prior evidence of association for these loci with disease risk. For the association analyses of all the approximately 200,000 variants on the iCOGS array with the breast cancer subtypes in mutation carriers, only associations with *P* < 5 × 10^−8^ were considered significant. The discussion of findings and the tables were extended to associations at *P* <10^−6^.

For variants associated with ER-positive or ER-negative breast cancer with *P* <0.01, we evaluated whether the associations may have been affected by a possible survival bias due to inclusion of prevalent breast cancer cases in the analysis. For the sensitivity analysis, the association analysis by ER status was repeated after excluding mutation carriers diagnosed with breast cancer ≥5 years prior to study recruitment.

We evaluated the consistency between the breast cancer association estimates of previously reported breast cancer susceptibility variants in the general population (using published data) and the association estimates in *BRCA1* and *BRCA2* carriers using the intraclass correlation (ICC). We estimated ICC as outlined by Shrout and Fleiss [24] based on a one-way random-effects model and tested for agreement in absolute values of log HR. The same approach was used to evaluate the agreement between associations with ER-positive and/or -negative breast cancer in the general population and associations with ER-positive and/or -negative breast cancer in *BRCA1* and in *BRCA2* carriers. Furthermore, we carried out the same comparisons between associations for *BRCA1* and associations for *BRCA2* carriers.

## Results

### Subtype patterns

The analyses included data from 15,252 *BRCA1* carriers and 8,211 *BRCA2* carriers. Among the breast cancer–affected *BRCA1* carriers, we had data on at least one disease characteristic of interest for 4,619 (59%) of the 7,797 affected women (Table 1). Data were available on tumor characteristics for 2,570 (59%) of the 4,330 affected *BRCA2* carriers. Of the individuals with pathology information, 74% of the *BRCA1* carriers and 75% of the *BRCA2* carriers had data on ER status.

**Table 1:** **Breast tumor characteristics of 7,797 affected** ***BRCA1*** **mutation carriers and 4,330 affected** ***BRCA2*** **mutation carriers** ^**a**^

	*BRCA1* mutation carriers			*BRCA2* mutation carriers		
	Yes, *n* (%)	No, *n* (%)	Unknown status	Yes, *n* (%)	No, *n* (%)	Unknown status
Predictive markers						
ER-positive	819 (24)	2,639 (76)	4,339	1,490 (77)	434 (23)	2,406
PR-positive	662 (21)	2,485 (79)	4,650	1,099 (65)	591 (35)	2,640
HER2-positive	182 (9)	1,816 (91)	5799	121 (13)	847 (87)	3,362
Non-TN	580 (31)	1,310 (69)	5907	760 (85)	136 (15)	3,434
Morphology			3,789			2,087
Ductal	3,159 (82)			1,770 (79)		
Lobular	89 (2)			188 (8)		
Medullary	290 (6)			39 (2)		
Other	470 (10)			246 (11)		
Grade			4,645			3,840
Grade 1	81 (3)			113 (7)		
Grade 2	574 (18)			700 (42)		
Grade 3	2,497 (79)			839 (51)		
Nodal involvement	1,103 (33)	2,274 (67)	4,420	804 (43)	1,068 (57)	2,458
Stage			5,991			3,382
Stage 0^b^	65 (4)			121 (13)		
Stage 1	825 (46)			327 (35)		
Stage 2	772 (43)			390 (41)		
Stage 3	127 (7)			96 (10)		
Stage 4	17 (1)			14 (1)		

^a^ER, Estrogen receptor positive; HER2, Human epidermal growth factor receptor 2; PR, Progesterone receptor; TN, Triple-negative. ^b^Carcinoma *in situ*.

### Single-nucleotide polymorphism associations

After quality control, genotype data were available for analysis for 200,720 SNPs for *BRCA1* carriers and for 200,908 SNPs for *BRCA2* carriers. After adjusting for sample size and excluding SNPs chosen for inclusion on the genotyping array based on reported associations in subsets of the current sample, the inflation coefficient λ_1000_ values were 1.01 for ER-positive disease in *BRCA1* carriers, 1.02 for ER-negative in *BRCA1*, 1.01 for ER-positive in *BRCA2* and 1.02 for ER-negative in *BRCA2* carriers (Additional file 1: Figure S1 and Figure S2). Similar patterns were observed for other tumor characteristics (results not shown). After excluding variants located at known breast cancer susceptibility loci, there was no evidence for an excess in associations by ER status beyond the number expected.

### Associations of previously reported breast cancer susceptibility loci

#### Associations with overall breast cancer and by tumor estrogen receptor status

First, we considered the associations with risk for overall breast cancer and for tumor subtypes for the 74 breast cancer susceptibility variants that have been reported up to April 2013. In light of the strong prior evidence of association, we considered associations at *P* <0.05 as evidence that a previously reported breast cancer susceptibility allele also modifies overall or ER-specific breast cancer risk in mutation carriers. The associations with overall breast cancer risk and risk of breast cancer subtypes for all 74 variants are given in Table 2 and Additional file 1: Tables S4 to S10. Of the breast cancer susceptibility loci that had not previously been evaluated for an association in mutation carriers, SNPs at 5q33.3, 8q24.21, 11q24.3, 12q22, 16q12.1, 22q13.1 were associated with overall breast cancer risk for *BRCA1* carriers, and SNPs at 6p23, 11q24.3 and 16q12.1 were associated with breast cancer risk for *BRCA2* carriers at *P* <0.05 (Table 2). Overall, 15 breast cancer susceptibility variants were associated with ER-negative breast cancer in *BRCA1* carriers and 8 variants in *BRCA2* carriers at *P* <0.05 (Table 2). Ten significant associations with ER-positive breast cancer in *BRCA1* carriers and fourteen in *BRCA2* carriers were found. The strongest association with ER-positive breast cancer was observed for rs2981579 in *FGFR2* at 10q26.12 for both *BRCA1* and *BRCA2* carriers. SNP rs10069690 in *TERT* at 5p15.33 displayed the strongest association with ER-negative breast cancer for *BRCA1* carriers and rs9348512 at 6p24.3 for *BRCA2* carriers. We found significant differences in the associations by ER status for rs3803662 in *TOX3* at 16q12.1 (*P* = 2 × 10^−4^) and rs13387042 at 2q35 (*P* =0.002) for *BRCA1* carriers, which were not previously seen. Both SNPs showed evidence of association with ER-positive breast cancer only. Similarly, six of the loci that did not show evidence of association with overall breast cancer were associated with ER-positive and two with ER-negative breast cancer in *BRCA1* carriers. This included two of the loci not previously evaluated in mutation carriers: 3q26.1 and 6p25.3. In *BRCA2* carriers, four of the variants lacking evidence of association with overall breast cancer were associated with ER-negative and three with ER-positive breast cancer. This included four loci not previously evaluated in mutation carriers: 2q24, 14q13.3, 19q13.31 and 22q12.2. Of the breast cancer susceptibility loci that had not yet been evaluated for an association with breast cancer in mutation carriers, rs1011970 at *CDKN2A/B* and rs1292011 at 12q24.21 had significantly different associations with ER-positive and ER-negative cancer for *BRCA1* carriers (*P*_het_ = 0.009 and *P*_het_ = 0.004, respectively, for the difference between ER-positive and ER-negative). SNP rs2236007 at 14q13.3 displayed differences by ER status for *BRCA2* carriers (*P*_het_ = 0.008). These three SNPs had associations in different directions for ER-positive and ER-negative tumors.

**Table 2:** **Associations of susceptibility loci with overall and estrogen receptor-positive and -negative breast cancer** ^**a**^

							*BRCA1* carriers								*BRCA2* carriers							
								Overall		ER-negative		ER-positive				Overall		ER-negative		ER-positive		
Locus	SNP	Position ^b^	Nearby gene	Ref ^c^	Eff ^d^	K ^e^	MAF	HR (95% CI)	*P* -value	HR (95% CI)	*P* -value	HR (95% CI)	*P* -value	*P*_het_-value ^f^	MAF	HR (95% CI)	*P* -value	HR (95% CI)	*P* -value	HR (95% CI)	*P* -value	*P*_het_-value ^f^
1p36.22	rs616488	10566215	*PEX14*	A	G	No	0.32	0.96 (0.92 to 1.01)	0.10	0.95 (0.90 to 1.00)	0.07	1.00 (0.90 to 1.10)	0.92	0.47	0.33	0.98 (0.92 to 1.04)	0.52	1.01 (0.88 to 1.16)	0.94	0.97 (0.9 to 1.05)	0.46	0.69
1p13.2	rs12022378	114448389	*SYT6*	G	A	No	0.16	1.03 (0.98 to 1.09)	0.25	1.03 (0.97 to 1.10)	0.31	1.03 (0.90 to 1.17)	0.68	0.94	0.17	1.03 (0.95 to 1.12)	0.42	1.16 (0.97 to 1.37)	0.10	1.00 (0.91 to 1.1)	1.00	0.15
1p11.2	rs11249433	121280613	*FCGR1B*	A	G	Yes	0.41	0.99 (0.95 to 1.04)	0.78	0.99 (0.94 to 1.03)	0.55	1.02 (0.93 to 1.13)	0.63	0.50	0.41	1.05 (0.99 to 1.12)	0.09	1.04 (0.91 to 1.19)	0.57	1.06 (0.99 to 1.13)	0.12	0.84
1q32.1a	rs6678914	202187176	*LGR6*	G	A	No	0.4	0.98 (0.94 to 1.02)	0.39	0.96 (0.91 to 1.01)	0.08	1.06 (0.97 to 1.17)	0.21	0.07	0.41	1.04 (0.98 to 1.10)	0.19	0.94 (0.82 to 1.08)	0.39	1.07 (1.00 to 1.15)	0.05	0.11
1q32.1b	rs4245739	204518842	*MDM4*	A	C	Yes	0.28	1.10 (1.05 to 1.15)	**4.6 × 10** ^**−5**^	1.12 (1.07 to 1.19)	**1.4 × 10** ^**−5**^	1.02 (0.91 to 1.13)	0.76	0.11	0.28	0.97 (0.91 to 1.04)	0.38	1.09 (0.95 to 1.26)	0.21	0.94 (0.87 to 1.01)	0.10	0.07
2p24.1	rs12710696	19320803	*OSR1*	G	A	No	0.39	1.01 (0.97 to 1.06)	0.51	1.01 (0.97 to 1.07)	0.57	1.01 (0.92 to 1.12)	0.77	1.00	0.38	1.00 (0.94 to 1.06)	0.99	1.14 (1.00 to 1.30)	**0.05**	0.96 (0.9 to 1.03)	0.28	**0.02**
2q14.2	rs4849887	121245122		G	A	No	0.11	1.02 (0.96 to 1.09)	0.53	1.01 (0.94 to 1.09)	0.78	1.05(0.90 to 1.23)	0.49	0.64	0.11	0.98 (0.89 to 1.08)	0.72	1.08 (0.87 to 1.33)	0.49	0.96 (0.85 to 1.07)	0.43	0.34
2q31.1	rs2016394	172972971	*DLX2*	G	A	No	0.47	1.01 (0.97 to 1.06)	0.54	1.03 (0.98 to 1.08)	0.23	0.96 (0.87 to 1.06)	0.39	0.21	0.46	0.99 (0.93 to 1.05)	0.74	1.07 (0.94 to 1.22)	0.32	0.97 (0.9 to 1.04)	0.36	0.21
2q31.1	rs1550623	174212894	*CDCA7*	A	G	No	0.15	1.01 (0.95 to 1.07)	0.72	1.02 (0.95 to 1.09)	0.54	0.97 (0.85 to 1.12)	0.72	0.57	0.15	0.97 (0.89 to 1.06)	0.49	1.03 (0.87 to 1.23)	0.70	0.95 (0.87 to 1.05)	0.31	0.41
2q35	rs13387042	217905832	*TNP1*	A	G	Yes	0.47	0.98 (0.94 to 1.02)	0.41	1.02 (0.97 to 1.07)	0.37	0.86 (0.78 to 0.95)	**1.9 × 10** ^**−3**^	**2.3 × 10** ^**−3**^	0.48	0.99 (0.93 to 1.05)	0.68	0.98 (0.87 to 1.12)	0.82	0.99 (0.93 to 1.06)	0.74	0.96
2q35	rs16857609	218296508	*DIRC3*	G	A	No	0.26	1.04 (1.00 to 1.09)	0.06	1.03 (0.98 to 1.09)	0.23	1.08 (0.97 to 1.21)	0.14	0.47	0.27	0.96 (0.90 to 1.03)	0.26	0.87 (0.74 to 1.01)	0.08	0.99 (0.92 to 1.07)	0.82	0.14
3p26.1	rs6762644	4742276	*ITPR1*	A	G	No	0.36	1.04 (0.99 to 1.08)	0.09	1.00 (0.95 to 1.05)	0.92	1.16 (1.05 to 1.28)	**2.9 × 10** ^**−3**^	**0.01**	0.37	0.98 (0.93 to 1.05)	0.61	1.08 (0.94 to 1.24)	0.27	0.96 (0.89 to 1.03)	0.24	0.13
3p24.1	rs4973768	27416013	*SLC4A7*	G	A	Yes	0.49	1.01 (0.97 to 1.06)	0.47	0.99 (0.95 to 1.04)	0.81	1.09 (0.99 to 1.19)	0.07	0.10	0.5	1.08 (1.02 to 1.15)	**7.8 × 10** ^**−3**^	1.05 (0.92 to 1.20)	0.46	1.10 (1.02 to 1.18)	**0.01**	0.58
3p24.1	rs12493607	30682939	*TGFBR2*	C	G	No	0.35	0.99 (0.95 to 1.04)	0.73	0.99 (0.94 to 1.04)	0.63	1.01 (0.92 to 1.11)	0.85	0.70	0.34	0.98 (0.92 to 1.04)	0.54	1.07 (0.93 to 1.22)	0.35	0.96 (0.89 to 1.03)	0.23	0.17
4q24	rs9790517	106084778	*TET2*	G	A	No	0.23	0.98 (0.94 to 1.03)	0.51	0.97 (0.92 to 1.03)	0.30	1.03 (0.92 to 1.15)	0.61	0.37	0.22	0.97 (0.91 to 1.05)	0.48	0.91 (0.77 to 1.07)	0.25	0.99 (0.91 to 1.08)	0.88	0.35
4q34.1	rs6828523	175846426	*EBF1*	C	A	No	0.11	1.03 (0.96 to 1.1)	0.41	1.03 (0.95 to 1.11)	0.47	1.02 (0.88 to 1.19)	0.78	0.94	0.1	0.98 (0.89 to 1.08)	0.72	1.05 (0.85 to 1.30)	0.64	0.96 (0.86 to 1.08)	0.51	0.49
5p15.33	rs10069690	1279790	*TERT*	G	A	Yes	0.28	1.21 (1.15 to 1.26)	**1.1 × 10** ^**−15**^	1.24 (1.18 to 1.31)	**2.7 × 10** ^**−15**^	1.09 (0.98 to 1.22)	0.09	**0.04**	0.27	1.11 (1.04 to 1.19)	**1.6 × 10** ^**−3**^	1.25 (1.08 to 1.44)	**3.2 × 10** ^**−3**^	1.08 (1.00 to 1.17)	0.06	0.09
5p15.33	rs7725218	1282414	*TERT*	G	A	Yes	0.36	1.08 (1.04 to 1.13)	**3.2 × 10** ^**−4**^	1.09 (1.04 to 1.15)	**6.4 × 10** ^**−4**^	1.05 (0.95 to 1.15)	0.37	0.47	0.36	1.06 (0.99 to 1.12)	0.08	1.07 (0.93 to 1.23)	0.35	1.05 (0.98 to 1.13)	0.17	0.85
5p15.33	rs2736108	1297488	*TERT*	G	A	Yes	0.29	0.89 (0.85 to 0.93)	**4.2 × 10** ^**−7**^	0.86 (0.82 to 0.91)	**1.1 × 10** ^**−7**^	0.98 (0.88 to 1.08)	0.68	**0.04**	0.3	0.93(0.88 to 1.00)	**0.04**	0.91 (0.78 to 1.05)	0.20	0.94 (0.87 to 1.02)	0.13	0.67
5p12	rs10941679	44706498	*MRPS30*	A	G	Yes	0.25	0.99 (0.94 to 1.04)	0.61	0.99 (0.94 to 1.05)	0.84	0.97 (0.86 to 1.08)	0.54	0.66	0.24	1.07 (1.00 to 1.15)	**0.03**	1.09 (0.94 to 1.27)	0.24	1.07 (0.99 to 1.15)	0.09	0.78
5q11.2	rs889312	56031884	*MAP3K1*	A	C	Yes	0.29	1.01 (0.97 to 1.06)	0.52	0.99 (0.94 to 1.05)	0.82	1.09 (0.98 to 1.21)	0.12	0.15	0.3	1.04 (0.98 to 1.11)	0.21	0.99 (0.86 to 1.14)	0.90	1.06 (0.98 to 1.14)	0.14	0.44
5q11.3	rs10472076	58184061	*RAB3C*	A	G	No	0.37	1.00 (0.96 to 1.05)	0.83	0.98 (0.94 to 1.03)	0.52	1.08 (0.98 to 1.18)	0.13	0.11	0.38	0.99 (0.93 to 1.05)	0.78	1.02 (0.89 to 1.17)	0.78	0.98 (0.92 to 1.05)	0.64	0.66
5q11.3	rs1353747	58337481	*PDE4D*	A	C	No	0.09	0.98 (0.91 to 1.05)	0.53	0.95 (0.88 to 1.04)	0.28	1.06 (0.90 to 1.24)	0.50	0.29	0.09	0.95 (0.86 to 1.05)	0.35	1.02 (0.81 to 1.27)	0.88	0.94 (0.83 to 1.05)	0.26	0.52
5q33.3	rs1432679	158244083		A	G	No	0.44	1.05 (1.00 to 1.09)	**0.03**	1.03 (0.98 to 1.08)	0.25	1.10 (1.01 to 1.21)	**0.04**	0.21	0.45	1.01 (0.95 to 1.07)	0.79	0.92 (0.81 to 1.04)	0.16	1.04 (0.97 to 1.11)	0.30	0.09
6p25.3	rs11242675	1318878	*FOXQ1*	A	G	No	0.35	0.96 (0.92 to 1)	0.06	0.94 (0.90 to 0.99)	**0.03**	1.01 (0.91 to 1.11)	0.90	0.28	0.36	1.01 (0.95 to 1.07)	0.79	1.02 (0.89 to 1.18)	0.75	1.00 (0.93 to 1.08)	0.90	0.83
6p24.3	rs9348512	10456706	*TFAP2A*	C	A	Yes	0.34	1.00 (0.95 to 1.04)	0.87	1.00 (0.95 to 1.05)	0.88	1.00 (0.90 to 1.10)	0.95	1.00	0.34	0.85 (0.8 to 0.9)	**9.2 × 10** ^**−8**^	0.79 (0.69 to 0.91)	**1.1 × 10** ^**−3**^	0.86 (0.8 to 0.92)	**3.4 × 10** ^**−5**^	0.32
6p23	rs204247	13722523	*RANBP9*	A	G	No	0.44	1.00 (0.96 to 1.04)	0.98	1.00 (0.95 to 1.04)	0.84	1.01 (0.92 to 1.12)	0.75	0.72	0.44	1.09 (1.03 to 1.15)	**3.4 × 10** ^**−3**^	1.08 (0.94 to 1.24)	0.25	1.09 (1.02 to 1.17)	**9.7 × 10** ^**−3**^	0.93
6q14	rs17530068	82193109	*FAM46A*	A	G	Yes	0.25	1.03 (0.98 to 1.08)	0.23	1.03 (0.97 to 1.09)	0.29	1.03 (0.92 to 1.14)	0.63	0.95	0.25	1.10 (1.03 to 1.18)	**7.2 × 10** ^**−3**^	1.07 (0.92 to 1.25)	0.40	1.11 (1.02 to 1.2)	**0.01**	0.70
6q25.1	rs3757318	151914113	*ESR1*	G	A	Yes	0.08	1.20 (1.11 to 1.29)	**1.1 × 10** ^**−6**^	1.24 (1.14 to 1.35)	**5.6 × 10** ^**−7**^	1.06 (0.89 to 1.26)	0.51	0.12	0.09	1.15 (1.03 to 1.28)	**0.01**	1.33 (1.07 to 1.65)	**9.1 × 10** ^**−3**^	1.09 (0.96 to 1.24)	0.17	0.12
6q25.1	rs2046210	151948366	*ESR1*	G	A	Yes	0.37	1.16 (1.12 to 1.21)	**2.4 × 10** ^**−12**^	1.20 (1.15 to 1.26)	**2.8 × 10** ^**−13**^	1.04 (0.94 to 1.15)	0.42	**0.01**	0.37	1.06 (0.99 to 1.12)	0.07	1.15 (1.00 to 1.33)	**0.04**	1.03 (0.96 to 1.1)	0.43	0.17
7q35	rs720475	144074929	*ARHGEF5*	G	A	Yes	0.26	0.98 (0.93 to 1.03)	0.36	0.98 (0.93 to 1.04)	0.54	0.96 (0.86 to 1.08)	0.53	0.79	0.26	0.99 (0.92 to 1.06)	0.71	0.94 (0.80 to 1.09)	0.41	1.00 (0.93 to 1.08)	0.96	0.45
8p12	rs9693444	29509616	*DUSP4*	C	A	Yes	0.33	1.01 (0.96 to 1.05)	0.80	1.01 (0.96 to 1.06)	0.69	0.99 (0.90 to 1.09)	0.83	0.72	0.33	0.98 (0.93 to 1.05)	0.61	0.92 (0.80 to 1.07)	0.28	1.00 (0.94 to 1.07)	0.94	0.32
8q21.11	rs6472903	76230301		A	C	Yes	0.17	1.01 (0.96 to 1.07)	0.74	1.01 (0.95 to 1.08)	0.77	1.01 (0.89 to 1.14)	0.89	0.99	0.16	0.97 (0.90 to 1.05)	0.48	0.95 (0.79 to 1.14)	0.56	0.98 (0.89 to 1.07)	0.66	0.75
8q21.11	rs2943559	76417937	*HNF4G*	A	G	Yes	0.08	1.06 (0.98 to 1.14)	0.12	1.07 (0.98 to 1.17)	0.12	1.02 (0.86 to 1.22)	0.78	0.68	0.09	1.10 (1.00 to 1.22)	0.05	1.14 (0.91 to 1.43)	0.27	1.09 (0.97 to 1.23)	0.13	0.77
8q24.21	rs11780156	129194641	*MYC*	G	A	No	0.19	0.95 (0.9 to 1)	**0.05**	0.96 (0.90 to 1.02)	0.15	0.93 (0.82 to 1.05)	0.23	0.70	0.19	0.97 (0.90 to 1.04)	0.44	0.95 (0.81 to 1.12)	0.56	0.98 (0.9 to 1.06)	0.60	0.79
8q24.21	rs13281615	128355618		A	G	Yes	0.43	1.02 (0.98 to 1.06)	0.44	1.01 (0.96 to 1.06)	0.73	1.04 (0.95 to 1.15)	0.38	0.55	0.43	1.03 (0.97 to 1.09)	0.33	1.05 (0.92 to 1.20)	0.44	1.02 (0.96 to 1.1)	0.51	0.71
9p21.3	rs1011970	22062134	*CDKN2B*	C	A	Yes	0.17	1.02 (0.97 to 1.08)	0.47	1.07 (1.00 to 1.14)	0.05	0.87 (0.76 to 0.99)	**0.04**	**9.3 × 10** ^**−3**^	0.17	1.03 (0.95 to 1.11)	0.50	1.11 (0.94 to 1.32)	0.21	1.00 (0.92 to 1.1)	0.95	0.29
9q31.2	rs10759243	110306115	*KLF4*	C	A	No	0.31	0.98 (0.94 to 1.02)	0.39	0.98 (0.93 to 1.03)	0.49	0.98 (0.88 to 1.08)	0.64	0.93	0.29	1.00 (0.94 to 1.06)	0.94	0.94 (0.81 to 1.09)	0.40	1.01 (0.94 to 1.09)	0.69	0.37
9q31.2	rs865686	110888478	*KLF4*	A	C	Yes	0.36	0.99 (0.95 to 1.04)	0.77	1.02 (0.97 to 1.07)	0.52	0.92 (0.83 to 1.02)	0.11	0.10	0.36	0.99 (0.93 to 1.05)	0.75	1.16 (1.01 to 1.34)	**0.04**	0.94 (0.88 to 1.01)	0.12	**0.01**
10p12.31	rs7072776	22032942	*MLLT10*	G	A	No	0.31	0.99 (0.94 to 1.03)	0.52	0.96 (0.91 to 1.01)	0.11	1.08 (0.98 to 1.20)	0.12	**0.04**	0.3	0.99 (0.92 to 1.05)	0.67	0.88 (0.77 to 1.02)	0.08	1.02 (0.94 to 1.09)	0.66	0.09
10p12.31	rs11814448	22315843	*DNAJC1*	A	C	No	0.02			1.10 (0.94 to 1.29)	0.22	1.25 (0.93 to 1.69)	0.15	0.50								
10q21.2	rs10995190	64278682	*ZNF365*	G	A	Yes	0.15	0.99 (0.93 to 1.05)	0.70	1.02 (0.96 to 1.09)	0.49	0.88 (0.76 to 1.00)	0.06	0.05	0.15	0.94 (0.86 to 1.02)	0.16	0.92 (0.76 to 1.12)	0.43	0.95 (0.86 to 1.04)	0.26	0.84
10q22.3	rs704010	80841148	*ZMIZ1*	G	A	Yes	0.37	1.01 (0.97 to 1.06)	0.48	0.99(0.94 to 1.04)	0.61	1.12 (1.01 to 1.23)	**0.03**	**0.03**	0.38	1.01 (0.95 to 1.07)	0.86	1.02 (0.90 to 1.16)	0.78	1.00 (0.93 to 1.07)	0.96	0.83
10q25.2	rs7904519	114773927	*TCF7L2*	A	G	Yes	0.47	1.09 (1.05 to 1.14)	**1.6 × 10** ^**−5**^	1.09 (1.04 to 1.14)	**6.4 × 10** ^**−4**^	1.12 (1.02 to 1.23)	**0.02**	0.59	0.47	1.02 (0.96 to 1.08)	0.56	0.89 (0.78 to 1.01)	0.07	1.06 (0.99 to 1.13)	0.10	**0.02**
10q26.12	rs2981579	123337335	*FGFR2*	G	A	Yes	0.42	0.99 (0.95 to 1.04)	0.81	0.92 (0.87 to 0.96)	**6.9 × 10** ^**−4**^	1.29 (1.17 to 1.43)	**3.1 × 10** ^**−7**^	**7.5 × 10** ^**−9**^	0.44	1.24 (1.16 to 1.31)	**5.4 × 10** ^**−12**^	1.05 (0.92 to 1.20)	0.44	1.29 (1.21 to 1.38)	**2.2 × 10** ^**−13**^	**7.3 × 10** ^**−3**^
10q26.12	rs11199914	123093901	*FGFR2*	G	A	No	0.33	1.02 (0.98 to 1.07)	0.27	1.04 (0.99 to 1.10)	0.09	0.96 (0.86 to 1.06)	0.43	0.17	0.33	0.95 (0.90 to 1.02)	0.15	0.90 (0.78 to 1.03)	0.13	0.97 (0.9 to 1.04)	0.44	0.33
11p15.5	rs3817198	1909006	*LSP1*	A	G	Yes	0.33	1.08 (1.03 to 1.13)	**6.5 × 10** ^**−4**^	1.08 (1.03 to 1.14)	**2.9 × 10** ^**−3**^	1.07 (0.97 to 1.18)	0.17	0.89	0.34	1.11 (1.04 to 1.18)	**9.3 × 10** ^**−4**^	0.98 (0.85 to 1.13)	0.80	1.15 (1.07 to 1.24)	**1.1 × 10** ^**−4**^	0.06
11q13.1	rs3903072	65583066	*SNX32*	C	A	No	0.47	0.99 (0.95 to 1.03)	0.69	1.00 (0.95 to 1.05)	0.94	0.96 (0.87 to 1.05)	0.39	0.44	0.47	0.97 (0.91 to 1.03)	0.34	0.89 (0.78 to 1.01)	0.07	1.00 (0.93 to 1.07)	0.91	0.12
11q13.3	rs554219	69331642	*CCND1*	C	G	Yes	0.12	1.03 (0.97 to 1.09)	0.37	1.00 (0.93 to 1.08)	0.96	1.12 (0.97 to 1.29)	0.12	0.20	0.13	1.09 (1.00 to 1.19)	**0.05**	0.90 (0.73 to 1.10)	0.31	1.15 (1.04 to 1.26)	**5.0 × 10** ^**−3**^	**0.04**
11q13.3	c11_pos69088342	69379161		C	A	Yes	0.06	1.03 (0.95 to 1.13)	0.45	1.00 (0.90 to 1.12)	0.95	1.14 (0.93 to 1.39)	0.20	0.30	0.06	1.07 (0.95 to 1.21)	0.28	0.85 (0.64 to 1.14)	0.29	1.14 (0.99 to 1.3)	0.08	0.09
11q13.3	rs494406	69344241		G	A	Yes	0.25	1.02 (0.97 to 1.07)	0.46	1.02 (0.97 to 1.08)	0.42	1.00 (0.90 to 1.12)	0.97	0.74	0.26	1.05 (0.98 to 1.12)	0.16	1.03 (0.89 to 1.19)	0.66	1.05 (0.98 to 1.14)	0.18	0.80
11q24.3	rs11820646	129461171	*BARX2*	G	A	No	0.39	0.93 (0.89 to 0.97)	**5.9 × 10** ^**−4**^	0.94 (0.90 to 0.99)	**0.03**	0.88 (0.79 to 0.97)	**8.9 × 10** ^**−3**^	0.20	0.39	0.92 (0.86 to 0.97)	**4.2 × 10** ^**−3**^	0.95 (0.83 to 1.09)	0.45	0.91 (0.84 to 0.97)	**5.9 × 10** ^**−3**^	0.56
12p13.1	rs12422552	14413931	*ATF7IP*	G	C	No	0.27	1.01 (0.97 to 1.06)	0.63	1.01 (0.96 to 1.07)	0.68	1.01 (0.91 to 1.13)	0.85	0.99	0.28	1.00 (0.93 to 1.06)	0.92	1.02 (0.88 to 1.19)	0.79	0.99 (0.92 to 1.07)	0.79	0.73
12p11.22	rs10771399	28155080	*PTHLH*	A	G	Yes	0.10	0.85 (0.80 to 0.91)	**1.9 × 10** ^**−6**^	0.83 (0.77 to 0.9)	**1.2 × 10** ^**−5**^	0.91 (0.78 to 1.06)	0.24	0.36	0.1	0.89 (0.81 to 0.98)	**0.02**	0.72 (0.56 to 0.91)	**6.6 × 10** ^**−3**^	0.94 (0.84 to 1.05)	0.25	0.05
12q22	rs17356907	96027759	*NTN4*	A	G	No	0.29	0.95 (0.91 to 1.00)	**0.03**	0.94 (0.90 to 1.00)	**0.03**	0.98 (0.88 to 1.09)	0.71	0.56	0.3	0.99 (0.93 to 1.06)	0.78	0.95 (0.83 to 1.10)	0.51	1.00 (0.93 to 1.08)	0.96	0.55
12q24.21	rs1292011	115836522	*TBX3*	A	G	Yes	0.41	1.00 (0.96 to 1.05)	0.82	1.04 (0.99 to 1.10)	0.08	0.88 (0.80 to 0.97)	**0.01**	**4.39 × 10** ^**−3**^	0.41	0.92 (0.87 to 0.98)	**0.01**	1.07 (0.94 to 1.23)	0.29	0.88 (0.82 to 0.95)	**5.7 × 10** ^**−4**^	**0.01**
13q13.1	rs11571833	32972626	*BRCA2*	T	A	No	0.01	1.02 (0.83 to 1.26)	0.83	1.05 (0.83 to 1.34)	0.66	0.92 (0.53 to 1.59)	0.75	0.66	0.03	0.95 (0.78 to 1.16)	0.62	0.91 (0.58 to 1.41)	0.66	0.97 (0.78 to 1.2)	0.76	0.80
14q13.3	rs2236007	37132769	*PAX9*	G	A	No	0.21	0.97 (0.92 to 1.02)	0.19	0.95 (0.90 to 1.01)	0.13	1.01 (0.90 to 1.14)	0.87	0.42	0.21	0.99 (0.92 to 1.07)	0.83	1.20 (1.03 to 1.40)	**0.02**	0.94 (0.86 to 1.02)	0.13	**8.5 × 10** ^**−3**^
14q24.1	rs2588809	68660428		G	A	No	0.19	0.96 (0.91 to 1.01)	0.09	0.95 (0.89 to 1.01)	0.13	0.97 (0.86 to 1.09)	0.56	0.85	0.2	1.01 (0.94 to 1.09)	0.82	0.95 (0.80 to 1.13)	0.55	1.03 (0.94 to 1.12)	0.55	0.44
14q24.1	rs999737	69034682	*RAD51L1*	G	A	Yes	0.21	0.96 (0.91 to 1.01)	0.09	0.98 (0.92 to 1.04)	0.43	0.90 (0.80 to 1.01)	0.07	0.22	0.22	0.97 (0.91 to 1.05)	0.48	0.95 (0.81 to 1.11)	0.53	0.98 (0.91 to 1.06)	0.65	0.72
14q32.11	rs941764	91841069	*CCDC88C*	A	G	No	0.34	1.03 (0.98 to 1.07)	0.23	1.02 (0.97 to 1.07)	0.47	1.05 (0.95 to 1.17)	0.34	0.62	0.34	1.03 (0.97 to 1.09)	0.39	1.00 (0.88 to 1.14)	0.97	1.04 (0.97 to 1.11)	0.32	0.61
16q12.1a	rs3803662	52586341	*TOX3*	G	A	Yes	0.29	1.06 (1.01 to 1.11)	**0.02**	1.01 (0.96 to 1.07)	0.65	1.22 (1.10 to 1.35)	**1.5 × 10** ^**−4**^	**2.39 × 10** ^**−3**^	0.29	1.24 (1.16 to 1.32)	**6.2 × 10** ^**−11**^	1.12 (0.97 to 1.30)	0.11	1.27 (1.18 to 1.36)	**1.5 × 10** ^**−10**^	0.15
16q12.1b	rs11075995	53855291	*FTO*	A	T	No	0.24	1.01 (0.96 to 1.06)	0.61	0.98 (0.93 to 1.04)	0.60	1.11 (0.99 to 1.24)	0.07	0.07	0.24	1.02(0.95 to 1.09)	0.59	1.01 (0.86 to 1.18)	0.94	1.02 (0.94 to 1.11)	0.57	0.85
16q12.1b	rs17817449	53813367	*FTO*	A	C	No	0.41	0.95 (0.91 to 0.99)	**0.02**	0.94 (0.89 to 0.98)	**9.8 × 10** ^**−3**^	1.00 (0.91 to 1.10)	0.99	0.26	0.41	0.94 (0.88 to 1.00)	**0.03**	1.05 (0.91 to 1.21)	0.49	0.91 (0.85 to 0.97)	**6.6 × 10** ^**−3**^	0.08
16q23.2	rs13329835	80650805	*CDYL2*	A	G	No	0.23	1.04 (0.99 to 1.09)	0.09	1.03 (0.97 to 1.09)	0.29	1.08 (0.97 to 1.21)	0.17	0.48	0.24	1.03 (0.96 to 1.11)	0.35	0.94 (0.81 to 1.10)	0.43	1.06 (0.98 to 1.15)	0.13	0.17
17q22	rs6504950	53056471	*COX11*	G	A	Yes	0.27	0.98 (0.94 to 1.03)	0.49	0.99 (0.94 to 1.04)	0.70	0.96 (0.87 to 1.07)	0.49	0.68	0.27	1.04 (0.97 to 1.11)	0.24	1.08 (0.94 to 1.24)	0.27	1.03 (0.95 to 1.11)	0.46	0.54
18q11.2	rs527616	24337424	*AQP4*	C	G	No	0.37	0.99 (0.95 to 1.03)	0.61	0.97 (0.93 to 1.02)	0.30	1.04 (0.94 to 1.15)	0.42	0.26	0.37	0.96 (0.9 to 1.02)	0.19	0.95 (0.83 to 1.08)	0.41	0.96 (0.9 to 1.03)	0.30	0.82
18q11.2	rs1436904	22824665		A	C	No	0.39	0.99 (0.95 to 1.03)	0.68	1.00 (0.95 to 1.05)	0.86	0.98 (0.89 to 1.08)	0.63	0.74	0.39	0.96 (0.9 to 1.02)	0.14	1.02 (0.89 to 1.17)	0.79	0.94 (0.87 to 1.01)	0.07	0.30
19p13.11	rs8170	17389704	*BABAM1*	G	A	Yes	0.19	1.19 (1.12 to 1.25)	**2.9 × 10** ^**−10**^	1.22 (1.15 to 1.30)	**1.7 × 10** ^**−10**^	1.06 (0.95 to 1.20)	0.30	0.05	0.19	0.98 (0.91 to 1.06)	0.62	1.06 (0.90 to 1.25)	0.51	0.96 (0.88 to 1.05)	0.37	0.33
19p13.11	rs4808801	18571141	*ELL*	A	G	No	0.32	0.98 (0.94 to 1.02)	0.40	0.99 (0.94 to 1.05)	0.81	0.94 (0.85 to 1.04)	0.23	0.35	0.33	0.97 (0.91 to 1.03)	0.33	1.07 (0.93 to 1.23)	0.33	0.94 (0.87 to 1.01)	0.10	0.11
19q13.31	rs3760982	44286513	*KCNN4*	G	A	No	0.46	1.03 (0.99 to 1.08)	0.10	1.03 (0.98 to 1.08)	0.19	1.04 (0.95 to 1.14)	0.41	0.90	0.46	1.05 (0.99 to 1.12)	0.09	0.97 (0.86 to 1.11)	0.69	1.08 (1.01 to 1.15)	**0.03**	0.19
21q21.1	rs2823093	16520832	*NRIP1*	G	A	Yes	0.27	0.95 (0.91 to 1)	**0.04**	0.96 (0.91 to 1.02)	0.17	0.92 (0.82 to 1.02)	0.11	0.44	0.27	0.94 (0.88 to 1.01)	0.09	1.07 (0.92 to 1.25)	0.36	0.91 (0.84 to 0.98)	**0.02**	0.06
22q12.2	rs132390	29621477	*EMID1*	A	G	No	0.03	0.98 (0.87 to 1.1)	0.75	0.93 (0.81 to 1.07)	0.30	1.16 (0.91 to 1.46)	0.23	0.13	0.04	1.17 (1.00 to 1.37)	0.05	0.92 (0.61 to 1.39)	0.70	1.24 (1.04 to 1.49)	**0.02**	0.22
22q13.1	rs6001930	40876234	*SGSM3*	A	G	No	0.11	1.07 (1.00 to 1.14)	**0.03**	1.06 (0.98 to 1.15)	0.14	1.11 (0.96 to 1.30)	0.17	0.60	0.10	1.02 (0.92 to 1.12)	0.71	0.94 (0.76 to 1.18)	0.61	1.04 (0.93 to 1.16)	0.48	0.44

^a^CI, Confidence interval; ER, Estrogen receptor; HR, Hazard ratio; MAF, Mean allele frequency; SNP, Single-nucleotide polymorphism. Results represent *BRCA1* and *BRCA2* mutation carriers for 74 previously reported breast cancer (BC) susceptibility variants from population-based studies. *P*-values <0.05 are shown in bold. ^b^Position in build 37. ^c^Reference allele. ^c^Effect allele. ^e^Association in *BRCA1* and *BRCA2* carriers has been reported before. ^f^*P*-value for the difference between the association with ER-positive BC and the association with ER-negative BC.

When association analyses for ER-positive and -negative disease were repeated after excluding prevalent breast cancer cases (Additional file 1: Table S3), the HR estimates were consistent with the estimates from the complete sample but were associated with larger confidence intervals. Therefore, it is unlikely that our results are influenced by survival bias.

#### Associations with other subtypes and clinical features

The pattern of associations of previously reported breast cancer susceptibility variants by PR and TN status were very similar to that by ER status (Additional file 1: Tables S4 and S6), but fewer associations were observed at *P* <0.01. SNP rs720475 at 7q35 was the only variant that was associated with HER2-positive disease (HR = 1.45 and *P* = 0.003 for HER2-positive, *P*_het_ = 9 × 10^−4^ in *BRCA2* carriers) (Additional file 1: Table S5).

For *BRCA1* carriers, there were significant differences (*P*_het_ <0.01) in the HR for high grade (grade 3) and grades 1 and 2 breast cancer for SNPs at 10q26.12 (*FGFR2*) and at 12q24.21 (Additional file 1: Table S9). SNP rs3803662 in *TOX3* at 16q12.1 was associated exclusively with node-positive breast cancer (*P* = 2 × 10^−4^, *P*_het_ = 0.005) (Additional file 1: Table S10). This was also the only variant associated with lobular cancer, as shown in Additional file 1: Table S8 (*P* = 8 × 10^−6^ for *BRCA2* carriers). The HR for lobular cancer was larger than that for non-lobular cancer (lobular HR = 1.57, 95% CI: 1.29 to 1.92; non-lobular HR = 1.20, 95% CI: 1.13 to 1.28 for *BRCA2* carriers; *P*_het_ = 9 × 10^−4^). There was no evidence for differences in associations by histological grade and nodal involvement for *BRCA2* carriers.

#### Comparison of patterns of associations by breast cancer estrogen receptor status between BRCA1 and BRCA2 carriers and the general population

**Figure 1:**
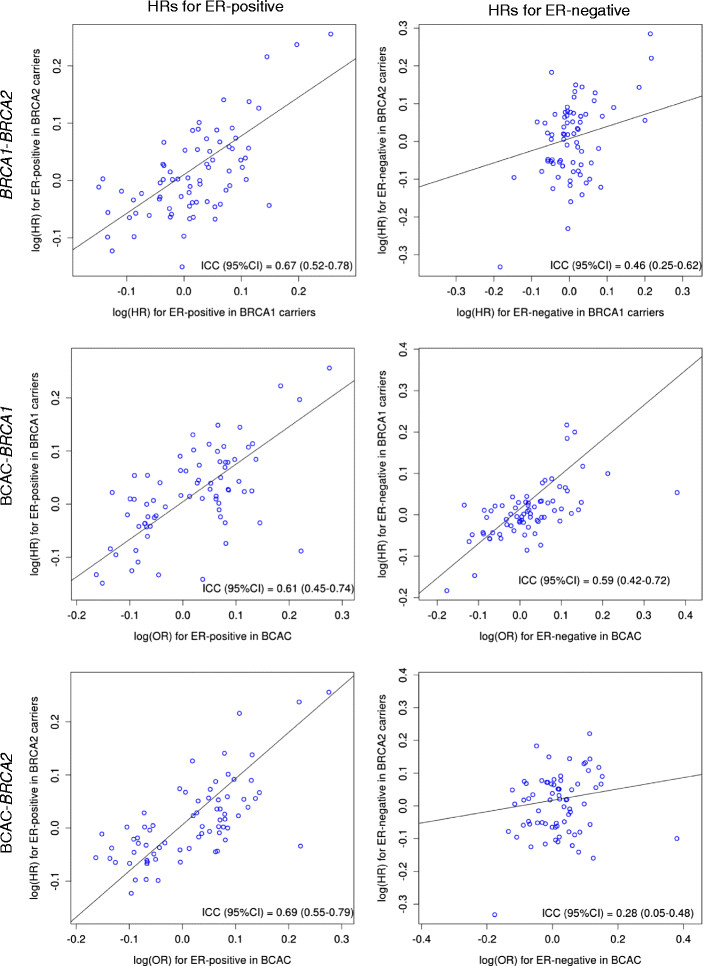
**Estrogen receptor-positive and -negative log hazard ratio estimates in the general population and in** ***BRCA1*** **and** ***BRCA2*** **carriers.** Dots represent the association of 74 previously reported breast cancer susceptibility single-nucleotide polymorphisms with estrogen receptor (ER)-positive breast cancer **(A–C)** and ER-negative breast cancer **(D–F)**. **(A)** and **(D)** compare associations between *BRCA1* and *BRCA2* carriers, **(B)** and **(E)** between the general population and *BRCA1* carriers and **(C)** and **(F)** between the general population and *BRCA2* carriers. Association results for the general population were taken from reports published by the Breast Cancer Association Consortium (BCAC) [8]-[10],[16].

We compared the log HR estimates for the breast cancer association of known breast cancer susceptibility variants for *BRCA1* carriers, *BRCA2* carriers, and for the general population using published data from the Breast Cancer Association Consortium (BCAC) [8]. The resulting ICC coefficients for log HR/OR estimates for all comparisons are shown in Table 3. Log HR estimates for overall breast cancer risk in *BRCA2* carriers were very similar to the log odds ratios (ORs) from the general population (ICC = 0.63, 95% CI: 0.47 to 0.75) (Additional file 1: Figure S3B), whereas there was only moderate correlation between the log HR estimates for *BRCA1* carriers and the log HR estimates from both other groups (ICC: *BRCA1*-BCAC estimates = 0.43, *BRCA1*-*BRCA2* = 0.46) (Additional file 1: Figure S3A,C). When comparing ER-positive specific associations, we found stronger agreement between the log HR/OR estimates than for overall breast cancer. The ICC estimates ranged from 0.61 (95% CI: 0.45 to 0.74) (Figure 1B) for BCAC-*BRCA1* to 0.69 (95% CI: 0.55 to 0.79) (Figure 1C) for BCAC-*BRCA2*. The ER-negative breast cancer log HR estimates in *BRCA1* carriers and the corresponding BCAC estimates were strongly correlated (ICC = 0.59, 95% CI: 0.42 to 0.72) (Figure 1E). However, the ER-negative breast cancer log HR estimates in *BRCA2* carriers were less strongly correlated with the corresponding estimates in *BRCA1* carriers (ICC = 0.46, 95% CI: 0.25 to 0.62) (Figure 1D) and in BCAC (ICC = 0.28, 95% CI: 0.05 to 0.48) (Figure 1F). There was no evidence that the ICC was different from 0 for the comparison between ER-positive associations in *BRCA1* carriers with ER-negative associations in *BRCA2* carriers and vice versa (Additional file 1: Figure S4B,C). Similarly, there was no significant correlation between log OR estimates for ER-positive breast cancer in BCAC with log HR estimates for ER-negative breast cancer in *BRCA1* and *BRCA2* carriers (Additional file 1: Figures S5B and S6B). There was only moderate correlation between log OR estimates for ER-negative breast cancer in the general population and log HR estimates for ER-positive breast cancer in *BRCA1* and *BRCA2* carriers (ICC = 0.39 and ICC = 0.34, respectively) (Additional file 1: Figures S5C and S6C).

**Table 3:** **Comparisons between the associations of 74 breast cancer susceptibility loci in** ***BRCA1*** **carriers, in** ***BRCA2*** **carriers, and in population-based studies** ^**a**^

		***BRCA1*** carriers			***BRCA2*** carriers		
		Overall	ER-positive	ER-negative	Overall	ER-positive	ER-negative
*BRCA2* carriers	Overall	0.46 (0.25 to 0.62)					
	ER-positive		0.67 (0.52 to 0.78)	0.13 (−0.10 to 0.35)			
	ER-negative		0.10 (−0.13 to 0.33)	0.46 (0.25 to 0.62)			
BCAC	Overall	0.43 (0.22 to 0.60)			0.63 (0.47 to 0.75)		
	ER-positive		0.61 (0.45 to 0.74)	0.16 (−0.07 to 0.38)		0.69 (0.55 to 0.79)	0.13 (−0.11 to 0.35)
	ER-negative		0.34 (0.12 to 0.53)	0.59 (0.42 to 0.72)		0.39 (0.17 to 0.57)	0.28 (0.05 to 0.48)

^a^ER, Estrogen receptor. Data are intraclass correlation coefficients and 95% confidence intervals, which describe associations with overall breast cancer as well as by ER status. Data are derived from published studies by the Breast Cancer Association Consortium (BCAC).

### Associations by subtype with all single-nucleotide polymorphisms on iCOGS

Variants in or near the known breast cancer susceptibility loci *TERT*, *ESR1* and 19p13.11 showed strong associations (*P* <10^−6^) with at least one of the categories in all subtype analyses in *BRCA1* carriers. The same was true for SNPs in *FGFR2* and *TOX3* in *BRCA2* carriers.

Variants on the iCOGS array that exhibited associations at *P* <10^−6^ with any of the breast cancer subtypes of ER-, PR-, HER2-positive or -negative and TN breast tumors are shown in Table 4. All the variants associated with ER-positive or ER-negative breast cancer at *P* <10^−6^ were located within known breast cancer susceptibility loci. Similar associations were observed with PR-positive and PR-negative breast cancer for *BRCA1* carriers. A previously unreported SNP at 2p13.2 was associated with HER2-positive breast cancer in *BRCA1* carriers. In *BRCA2* carriers, two previously unreported variants showed evidence of association in the analysis by PR status. Furthermore, SNPs at 8q12.1 near *TOX* were associated with HER2-positive cancer in *BRCA2* carriers. Only SNPs in the known breast cancer susceptibility loci *FGFR2* and *TOX3* were associated with non-TN breast cancer in *BRCA2* carriers, and none were associated with TN breast cancer at *P* <10^−6^.

**Table 4:** **Associations with tumor subtypes** ^**a**^

									Affected by subtype, ***n*** (MAF)				Positive		Negative		
Subtype	Sample	SNP	Locus	Position ^b^	Nearby gene	Ref ^c^	Eff ^d^	Reported ^e^	Positive	Negative	Unknown	Number unaffected (MAF)	HR (95% CI)	***P*** -value	HR (95% CI)	***P*** -value	***P***_het_-value ^f^
ER status	*BRCA1* carriers	rs10069690	5p15.33	1332790	*TERT*	G	A	Yes	819 (0.27)	2,635 (0.3)	4,331 (0.3)	7,449 (0.26)	1.09 (0.98 to 1.22)	0.09	1.24 (1.18 to 1.31)	2.7 × 10^−15^	0.04
		rs2046210	6q25.1	151990059	*C6orf97*	G	A	Yes	819 (0.36)	2,639 (0.39)	4,338 (0.38)	7,453 (0.35)	1.04 (0.94 to 1.15)	0.42	1.2 (1.15 to 1.26)	2.8 × 10^−13^	0.01
		rs2811708	9p21	21963422	*CDKN2A/B*	C	A	Yes	816 (0.24)	2,634 (0.31)	4,336 (0.28)	7,445 (0.28)	0.82 (0.73 to 0.91)	2.9 × 10^−4^	1.13 (1.07 to 1.19)	7.3 × 10^−6^	3.9 × 10^−7^
		rs45631579	10q26	123328965	*FGFR2*	A	G	Yes	819 (0.48)	2,639 (0.4)	4,337 (0.42)	7,455 (0.41)	1.29 (1.17 to 1.43)	2.6 × 10^−7^	0.92 (0.87 to 0.96)	4.7 × 10^−4^	5.1 × 10^−9^
		rs2590275	12p12	28057334	*PTHLH*	G	C	Yes	819 (0.26)	2,639 (0.25)	4,338 (0.26)	7,454 (0.28)	0.92 (0.83 to 1.03)	0.14	0.87 (0.82 to 0.92)	5.3 × 10^−7^	0.34
		rs2363956	19p13.11	17255124	*ANKLE1*	C	A	Yes	819 (0.47)	2,635 (0.53)	4,334 (0.52)	7,447 (0.48)	0.98 (0.89 to 1.08)	0.71	1.25 (1.19 to 1.31)	6.4 × 10^−20^	1.6 × 10^−5^
	*BRCA2* carriers	rs2162540	10q26	123342126	*FGFR2*	A	G	Yes	1450 (0.47)	426 (0.41)	2,348 (0.44)	3,783 (0.38)	1.34 (1.25 to 1.43)	3.6 × 10^−16^	1.08 (0.94 to 1.23)	0.27	6.1 × 10^−3^
		rs17271951	16q12.1	51095541	*TOX3*	A	G	Yes	1490 (0.32)	434 (0.28)	2,397 (0.3)	3,879 (0.25)	1.29 (1.2 to 1.39)	8.4 × 10^−12^	1.14 (0.99 to 1.31)	0.08	0.14
PR status	*BRCA1* carriers	rs10069690	5p15.33	1332790	*TERT*	G	A	Yes	662 (0.27)	2,481 (0.29)	4,642 (0.3)	7,449 (0.26)	1.10 (0.98 to 1.24)	0.09	1.24 (1.17 to 1.3)	1.3 × 10^−14^	0.11
		rs2046210	6q25.1	151990059	*C6orf97*	G	A	Yes	662 (0.37)	2,485 (0.39)	4,649 (0.38)	7,453 (0.35)	1.09 (0.98 to 1.21)	0.12	1.18 (1.13 to 1.24)	2.0 × 10^−11^	0.19
		rs45631626	10q26	123327325	*FGFR2*	G	A	Yes	662 (0.49)	2,485 (0.4)	4,647 (0.42)	7,455 (0.42)	1.33 (1.2 to 1.48)	1.3 × 10^−7^	0.92 (0.88 to 0.97)	9.6 × 10^−4^	5.3 × 10^−9^
		rs2590275	12p12	28057334	*PTHLH*	G	C	Yes	662 (0.26)	2,485 (0.25)	4,649 (0.26)	7,454 (0.28)	0.92 (0.82 to 1.03)	0.16	0.87 (0.82 to 0.92)	8.2 × 10^−7^	0.45
		rs8100241	19p13.11	17253894	*ANKLE1*	G	A	Yes	657 (0.51)	2,473 (0.47)	4,628 (0.48)	7,420 (0.52)	0.98 (0.88 to 1.09)	0.71	0.81 (0.78 to 0.85)	1.8 × 10^−17^	2.4 × 10^−3^
	*BRCA2* carriers	rs10017576	4q28.3	139799629		G	A	No	1099 (0.38)	591 (0.43)	2,639 (0.41)	3,880 (0.43)	0.83 (0.77 to 0.89)	8.9 × 10^−7^	1.02 (0.92 to 1.14)	0.67	2.7 × 10^−3^
		rs4376461	8p12	32822117	*NRG1*	C	A	No	1099 (0.15)	591 (0.10)	2,640 (0.14)	3,881 (0.15)	1.00 (0.9 to 1.12)	0.93	0.6 (0.5 to 0.73)	2.4 × 10^−7^	1.4 × 10^−5^
		rs45631588	10q26	123341292	*FGFR2*	A	G	Yes	1099 (0.47)	591 (0.43)	2,640 (0.45)	3,881 (0.39)	1.33 (1.23 to 1.44)	2.7 × 10^−13^	1.15 (1.03 to 1.28)	0.01	0.04
		rs17271951	16q12.1	51095541	*TOX3*	A	G	Yes	1099 (0.32)	591 (0.28)	2,631 (0.3)	3,879 (0.25)	1.32 (1.21 to 1.43)	4.3 × 10^−11^	1.14 (1.01 to 1.29)	0.03	0.07
HER2 status	*BRCA1* carriers	rs17008885	2p13.2	73303647	*SMYD5*	T	A	No	182 (0.19)	1,816 (0.3)	5,799 (0.3)	7,455 (0.31)	0.54 (0.43 to 0.69)	7.8 × 10^−7^	0.99 (0.95 to 1.04)	0.78	3.8 × 10^−6^
		rs10069690	5p15.33	1332790	*TERT*	G	A	Yes	181 (0.23)	1,813 (0.29)	5,791 (0.3)	7,449 (0.26)	0.9 (0.71 to 1.13)	0.36	1.24 (1.18 to 1.3)	3.4 × 10^−17^	0.01
		rs2046210	6q25.1	151990059	*C6orf97*	G	A	Yes	182 (0.39)	1,816 (0.4)	5,798 (0.38)	7,453 (0.35)	1.12 (0.91 to 1.38)	0.29	1.17 (1.11 to 1.22)	1.5 × 10^−10^	0.71
		rs10843055	12p12	28063088	*PTHLH*	A	C	Yes	171 (0.06)	1,743 (0.06)	5,615 (0.06)	7,234 (0.07)	0.79 (0.52 to 1.2)	0.26	0.79 (0.72 to 0.87)	6.2 × 10^−7^	0.99
		rs4808616	19p13.11	17264033	*ANKLE1*	C	A	Yes	176 (0.35)	1,782 (0.32)	5,706 (0.32)	7,339 (0.28)	1.37 (1.11 to 1.69)	3.8 × 10^−3^	1.2 (1.14 to 1.26)	6.8 × 10^−13^	0.25
	*BRCA2* carriers	rs4305889	8q12.1	60352785	*TOX*	A	G	No	121 (0.18)	845 (0.09)	3,353 (0.11)	3,874 (0.1)	2.04 (1.53 to 2.71)	8.7 × 10^−7^	0.96 (0.85 to 1.08)	0.47	1.0 × 10^−5^
		rs45631588	10q26	123341292	*FGFR2*	A	G	Yes	121 (0.45)	847 (0.46)	3,362 (0.45)	3,881 (0.39)	1.27 (0.98 to 1.63)	0.07	1.28 (1.19 to 1.37)	2.1 × 10^−12^	0.95
		rs3817197	11p15.5	1862750	*LSP1*	G	A	Yes	121 (0.55)	847 (0.44)	3,353 (0.45)	3,877 (0.47)	1.30 (1.02 to 1.64)	0.03	0.84 (0.79 to 0.9)	9.1 × 10^−7^	1.3 × 10^−3^
		rs17271951	16q12.1	51095541	*TOX3*	A	G	Yes	121 (0.29)	847 (0.31)	3,353 (0.3)	3,879 (0.25)	1.10 (0.85 to 1.41)	0.47	1.27 (1.18 to 1.37)	6.9 × 10^−11^	0.3
Triple-negative^g^	*BRCA1* carriers	rs10069690	5p15.33	1332790	*TERT*	G	A	Yes	579 (0.27)	1,307 (0.3)	5,899 (0.3)	7,449 (0.26)	1.06 (0.94 to 1.19)	0.34	1.27 (1.2 to 1.36)	5.2 × 10^−14^	0.02
		rs2046210	6q25.1	151990059	*C6orf97*	G	A	Yes	580 (0.37)	1,310 (0.41)	5,906 (0.38)	7,453 (0.35)	1.01 (0.91 to 1.13)	0.79	1.23 (1.16 to 1.31)	5.5 × 10^−12^	6.8 × 10^−3^
		rs9512729	13q12.2	26974865	*LNX2*	G	A	No	580 (0.34)	1,309 (0.43)	5,902 (0.41)	7,454 (0.41)	0.76 (0.69 to 0.85)	9.1 × 10^−7^	1.08 (1.02 to 1.15)	0.01	1.2 × 10^−6^
		rs8100241	19p13.11	17253894	*ANKLE1*	G	A	Yes	577 (0.5)	1,304 (0.46)	5,877 (0.48)	7,420 (0.52)	0.94 (0.85 to 1.04)	0.22	0.81 (0.76 to 0.86)	2.4 × 10^−13^	0.03
	*BRCA2* carriers	rs2162540	10q26	123342126	*FGFR2*	A	G	Yes	735 (0.46)	133 (0.36)	3,356 (0.44)	3,783 (0.38)	1.39 (1.29 to 1.49)	9.8 × 10^−20^	0.83 (0.67 to 1.03)	0.10	4.0 × 10^−5^
		rs1362548	16q12.1	51121452	*TOX3*	C	G	Yes	760 (0.32)	136 (0.29)	3,434 (0.31)	3,881 (0.26)	1.26 (1.17 to 1.36)	1.0 × 10^−9^	1.18 (0.94 to 1.49)	0.16	0.63

^a^CI, Confidence interval; HR, Hazard ratio; MAF, Mean allele frequency. Single-nucleotide polymorphisms (SNPs) associated at *P* <10^−6^ with breast cancer by tumor estrogen receptor (ER) status, progesterone receptor (PR) status, human epidermal growth factor receptor 2 (HER2) status or triple-negative (negative for ER, PR and HER2) are shown. The most strongly associated SNP from each locus is reported. ^b^Position in build 36. ^c^Reference allele. ^d^Effect allele. ^e^Variant located in previously reported breast cancer susceptibility locus. ^f^*P*-value for the difference in association between subtype positive and subtype negative breast cancer (for example, ER-positive vs ER-negative). ^g^Non–triple negative was considered as “positive” and triple-negative as “negative.”

A SNP at 7q36 was associated with ductal subtype for *BRCA1* carriers (Table 5). Two loci were associated with lobular breast cancer for *BRCA2* carriers: 11q23.3 and Xp11.23.

**Table 5:** **Associations with ductal and lobular breast cancer** ^**a**^

									N tumors with morphology (MAF)				Other morphology		Tumors with morphology		
Subtype	Sample	SNP	Locus	Position ^b^	Nearby gene	Ref ^c^	Eff ^d^	Reported ^e^	Present	Other	Unknown	Number unaffected (MAF)	HR (95% CI)	***P*** -value	HR (95% CI)	***P*** -value	***P***_het_-value ^f^
Ductal	*BRCA1* carriers	rs10069690	5p15.33	1332790	*TERT*	G	A	Yes	3,155 (0.29)	847 (0.30)	3,783 (0.30)	7,449 (0.26)	1.23 (1.11 to 1.36)	7.3 × 10^−5^	1.20 (1.14 to 1.26)	2.0 × 10^−11^	0.67
		rs11155803	6q25.1	151987362	*C6orf97*	A	G	Yes	3,159 (0.36)	848 (0.35)	3,786 (0.36)	7,452 (0.32)	1.13 (1.03 to 1.25)	0.01	1.17 (1.11 to 1.23)	4.6 × 10^−10^	0.58
		rs10252939	7q36	155587448		A	G	No	3,159 (0.28)	849 (0.33)	3,789 (0.29)	7,455 (0.32)	1.08 (0.98 to 1.19)	0.14	0.87 (0.82 to 0.91)	1.0 × 10^−7^	2.7 × 10^−4^
		rs8100241	19p13.11	17253894	*ANKLE1*	G	A	Yes	3,137 (0.48)	844 (0.49)	3,777 (0.47)	7,420 (0.52)	0.87 (0.79 to 0.95)	2.8 × 10^−3^	0.84 (0.8 to 0.88)	1.5 × 10^−13^	0.51
	*BRCA2* carriers	rs2162540	10q26	123342126	*FGFR2*	A	G	Yes	1,728 (0.45)	458 (0.44)	2,038 (0.44)	3,783 (0.38)	1.24 (1.08 to 1.42)	1.8 × 10^−3^	1.30 (1.21 to 1.39)	3.1 × 10^−14^	0.55
		rs1362548	16q12.1	51121452	*TOX3*	C	G	Yes	1,770 (0.3)	473 (0.32)	2,087 (0.31)	3,881 (0.26)	1.32 (1.15 to 1.51)	4.8 × 10^−5^	1.23 (1.15 to 1.32)	7.3 × 10^−9^	0.36
Lobular	*BRCA2* carriers	rs2186703	11q23.3	115265717	*RPL15P15*	A	C	No	188 (0.07)	2,055 (0.03)	2,087 (0.04)	3,881 (0.03)	1.09 (0.93 to 1.27)	0.29	2.54 (1.78 to 3.62)	2.8 × 10^−7^	1.5 × 10^−5^
		rs55998524	Xp11.23	51082075	*NUDT10*	C	G	No	188 (0.11)	2,054 (0.05)	2,086 (0.06)	3,880 (0.05)	0.96 (0.83 to 1.1)	0.54	2.42 (1.77 to 3.32)	3.6 × 10^−8^	7.0 × 10^−8^

^a^CI, Confidence interval; HR, Hazard ratio; MAF, Mean allele frequency. Single-nucleotide polymorphisms (SNPs) associated at *P* <10^−6^ with ductal carcinomas and, for *BRCA2* mutation carriers, lobular carcinomas are shown. The most strongly associated SNP from each locus is reported. ^b^Position in build 36. ^c^Reference allele. ^d^Effect allele. ^e^Variant located in previously reported breast cancer susceptibility locus. ^f^*P*-value for the difference in SNP association between ductal breast tumors and non-ductal breast tumors and for lobular breast tumors and non-lobular breast tumors.

There was one novel association with high-grade tumors in *BRCA1* carriers (Table 6). Three previously unreported variants were associated with breast cancer nodal status in *BRCA1* carriers: SNPs at 4q24 in the *TET2* gene, at 5q32 in the *SH3RF2* gene and at 7p22 in an intron of *NXPH1*. For *BRCA2* carriers, only SNPs in *FGFR2* and *TOX3* exhibited associations at *P* <10^−6^ with breast cancer nodal status or histological grade.

**Table 6:** **Associations with grade and lymph node status** ^**a**^

									Affected by subtype, ***n*** (MAF)				Low-grade/no nodal involvement		High grade/nodal involvement		
Subtype	Sample	SNP	Locus	Position ^b^	Nearby gene	Ref ^c^	Eff ^d^	Reported ^e^	High-grade/node-positive	Low-grade/node-negative	Unknown	Number unaffected (MAF)	HR (95% CI)	***P*** -value	HR (95% CI)	***P*** -value	***P***_het_-value ^f^
Grade 3	*BRCA1* carriers	rs17651413	2q33.1	202677054	*KIAA2012*	A	G	No	2,495 (0.12)	655 (0.11)	4,644 (0.13)	7,454 (0.11)	1.01 (0.86 to 1.2)	0.86	1.20 (1.12 to 1.29)	8.4 × 10^−7^	0.08
		rs10069690	5p15.33	1332790	*TERT*	G	A	Yes	2,493 (0.3)	654 (0.29)	4,638 (0.3)	7,449 (0.26)	1.15 (1.02 to 1.29)	0.02	1.22 (1.16 to 1.29)	2.4 × 10^−13^	0.39
		c6_pos151989450	6q25.1	151989450	*C6orf97*	G	A	Yes	2,497 (0.11)	655 (0.08)	4,645 (0.1)	7,455 (0.08)	1.01 (0.83 to 1.24)	0.91	1.31 (1.21 to 1.42)	2.2 × 10^−11^	0.02
		rs8100241	19p13.11	17253894	*ANKLE1*	G	A	Yes	2,483 (0.48)	653 (0.52)	4,622 (0.48)	7,420 (0.52)	0.95 (0.86 to 1.05)	0.36	0.82 (0.78 to 0.86)	2.2 × 10^−16^	0.01
	*BRCA2* carriers	rs45631588	10q26	123341292	*FGFR2*	A	G	Yes	839 (0.44)	813 (0.48)	2,678 (0.44)	3,881 (0.39)	1.36 (1.24 to 1.49)	7.4 × 10^−11^	1.19 (1.09 to 1.3)	1.7 × 10^−4^	0.06
		rs35850695	16q12.1	51131844	*TOX3*	G	A	Yes	839 (0.32)	813 (0.3)	2,678 (0.3)	3,881 (0.26)	1.2 (1.09 to 1.33)	3.0 × 10^−4^	1.31 (1.19 to 1.44)	4.0 × 10^−8^	0.26
Nodes	*BRCA1* carriers	rs1498125	4q24	106412012	*TET2*	A	G	No	1,103 (0.2)	2,274 (0.23)	4,419 (0.22)	7,455 (0.2)	1.18 (1.1 to 1.25)	4.4 × 10^−7^	0.98 (0.89 to 1.09)	0.74	5.7 × 10^−3^
		rs10069690	5p15.33	1332790	*TERT*	G	A	Yes	1,100 (0.3)	2,271 (0.29)	4,414 (0.3)	7,449 (0.26)	1.19 (1.12 to 1.26)	6.2 × 10^−9^	1.24 (1.13 to 1.35)	2.0 × 10^−6^	0.5
		rs11743632	5q32	145389263	*SH3RF2*	G	A	No	1,103 (0.31)	2,274 (0.37)	4,418 (0.36)	7,453 (0.37)	1.05 (0.99 to 1.11)	0.1	0.8 (0.74 to 0.87)	2.3 × 10^−7^	6.9 × 10^−7^
		rs9383936	6q25.1	151986307	*C6orf97*	G	A	Yes	1,103 (0.11)	2,274 (0.09)	4,420 (0.1)	7,455 (0.08)	1.16 (1.06 to 1.28)	1.3 × 10^−3^	1.41 (1.24 to 1.6)	1.3 × 10^−7^	0.03
		rs2349485	7p22	8517481	*NXPH1*	A	C	No	1,091 (0.37)	2,249 (0.34)	4,374 (0.34)	7,398 (0.37)	0.87 (0.82 to 0.92)	8.5 × 10^−7^	0.97 (0.90 to 1.06)	0.53	0.03
		rs11669059	19p13.11	17261453	*ANKLE1*	A	G	Yes	1,103 (0.41)	2,273 (0.4)	4,418 (0.39)	7,452 (0.43)	0.85 (0.81 to 0.9)	2.9 × 10^−9^	0.86 (0.80 to 0.93)	2.6 × 10^−4^	0.81
	*BRCA2* carriers	rs2981578	10q26	123330301	*FGFR2*	A	G	Yes	795 (0.44)	1,047 (0.48)	2,428 (0.47)	3,819 (0.52)	0.87 (0.8 to 0.94)	7.4 × 10^−4^	0.75 (0.68 to 0.82)	1.0 × 10^−9^	0.02
		rs35850695	16q12.1	51131844	*TOX3*	G	A	Yes	804 (0.31)	1,068 (0.3)	2,458 (0.3)	3,881 (0.26)	1.22 (1.12 to 1.33)	8.5 × 10^−6^	1.30 (1.18 to 1.44)	2.6 × 10^−7^	0.36

^a^CI, Confidence interval; HR, Hazard ratio; MAF, Mean allele frequency. Single-nucleotide polymorphisms (SNPs) associated at *P* <10^−6^ with high- or low-grade breast tumors and lymph node–positive or lymph node–negative breast cancer are shown. The most strongly associated SNPs from each locus are reported. ^b^Position in build 36. ^c^Reference allele. ^d^Effect allele. ^e^Variant located in previously reported breast cancer susceptibility locus. ^f^*P*-value for the difference in association between high-grade breast cancer and low-grade breast cancer for grade 3 and for the association with lymph node–positive breast cancer and lymph node–negative breast cancer.

## Discussion

This is the first comprehensive report, to our knowledge, of the associations of genetic variants with risk of developing breast cancer by tumor subtypes in *BRCA1* and *BRCA2* carriers. We evaluated the associations with ER, PR and HER2 status; morphologic subtype (ductal or lobular); histological grade; and lymph node status.

Prior to this study, variants at five loci (5p15.33, 6q25.1, 11p15.5, 12p11.22 and 16q12.1) had been shown to be associated with breast cancer risk for both *BRCA1* and *BRCA2* carriers; variants at four additional loci were known to be associated with breast cancer risk for *BRCA1* carriers only (1q32.1, 10q25.2, 14q24.1 and 19p13.11); and variants at six additional loci were known to be associated with risk for *BRCA2* carriers (3p24.1, 5p12, 6p24.3, 10q26.12, 11q13 and 12q24.21) [6],[7],[10]. Among the 43 breast cancer susceptibility variants that had not previously been evaluated in mutation carriers, we observed six associations with breast cancer at *P* <0.05 in *BRCA1* carriers (5p33.3, 8q24.21, 11q24.3, 12q22, 16q12.1b and 22q13.1) and three in *BRCA2* carriers (6p23, 11q24.3 and 16q12.1b).

After stratifying by ER status, we observed additional associations that were not seen for overall breast cancer. Among the 43 susceptibility variants that were evaluated in mutation carriers for the first time, we identified two additional associations in *BRCA1* carriers when stratifying by ER status (3q26.1, 6p25.3) and four in *BRCA2* carriers (2q24, 14q13.3, 19q13.3, 22q12.2). Population-based studies have shown that seven of the 74 breast cancer susceptibility variants display stronger associations with ER-negative disease in the general population [9]. Consistent with these findings, SNPs at 1q32.1 (*MDM4*), 5p15.33, 6q25.1 and 19p13 were associated with ER-negative breast cancer in *BRCA1* carriers and SNPs at 2p24.1, 5p15.33 and 6q25.1 in *BRCA2* carriers. No data were available for the SNP at 20q11.

We were able to confirm most of the associations with ER and PR subtypes of the 12 SNPs reported in the previous smaller CIMBA study [15]. In addition, two variants from that analysis now displayed evidence at *P* <0.05: rs13387042 at 2q35 with ER-positive breast cancer in *BRCA1* carriers and rs2046210 at 6q25.1 with ER-negative breast cancer in *BRCA2* carriers.

We also evaluated the associations of the 74 previously reported breast cancer susceptibility loci with other breast cancer subtypes. Variants at 5p15.33 (*TERT*), 6q25.1 (*ESR1*) and 19p13.11 showed associations in all subtype analyses for *BRCA1* carriers. These are the most strongly associated loci for overall breast cancer in *BRCA1* carriers, but they had not previously been investigated for their roles in subtypes other than ER. Variants at these three loci were associated with ER-, PR- and HER2-negative and TN subtypes. These variants were also associated with risk of high-grade tumors, with some suggestive evidence that this association was different from the association with grades 1 and 2 tumors for SNPs at *ESR1* and 19p13. The three loci were associated with ductal as well as non-ductal subtypes and node-positive as well as node-negative breast cancer. For *BRCA2* carriers, SNPs at loci 10q26 (*FGFR2*) and 16q12.1 (*TOX3*) were associated with all subtypes of breast cancer. SNPs at *FGFR2* and *TOX3* have consistently been associated with overall and with ER-positive breast cancer in population-based cases [25] as well as in *BRCA1/2* carriers [15]. Furthermore, SNPs at these two loci were associated with PR-positive and HER2-negative disease. There was no evidence for a difference in HR estimates by tumor grade, nodal involvement or morphologic subtype (ductal).

It is important to note that for each of the 74 known loci considered, we evaluated only the associations for the specific SNPs that have been reported by the BCAC. We have not considered all genetic variants within a given region. Therefore, we cannot rule out the possibility that more strongly associated variants exist at these loci than the SNPs reported here. Future fine-mapping efforts in conjunction with BCAC analyses should clarify this. Such studies may also identify the causal variant and together with subsequent functional studies gather insights about the functional mechanisms causing these association signals. This in turn may yield insights about the etiology of breast cancer in *BRCA1* and *BRCA2* carriers.

We compared HRs for the association with overall breast cancer and ER-positive and ER-negative breast cancer for all the 74 known breast cancer susceptibility variants between *BRCA1* carriers, *BRCA2* carriers and population-based studies using published data from BCAC. Although only some of these variants were associated at *P* <0.05 with breast cancer in mutation carriers as outlined above, there was nevertheless strong correlation between the HRs for overall breast cancer from the general population and those from *BRCA2* carriers, and moderate correlation between the HRs from *BRCA1* carriers and from BCAC. These results suggest that many of these variants may also be associated with breast cancer risk for mutation carriers, but the power to detect statistically significant associations in mutation carriers is low. These variants could be employed in risk prediction models for mutation carriers. Future studies should be aimed at assessing the associations of the combined effects of the SNPs in mutation carriers in terms of polygenic risk scores.

We used these comparisons to assess the hypothesis that observed differences in the associations between *BRCA1* and *BRCA2* carriers and the general population are mediated by ER status. The smaller correlation between the association estimates for *BRCA1* carriers and both BCAC and *BRCA2* carriers compared with those between BCAC and *BRCA2* carriers is consistent with this hypothesis. Moreover, we found stronger correlations between the HRs for ER-positive disease in all three two-way comparisons (ICC = 0.61 to 0.69). The correlation between ORs for ER-negative disease from BCAC and HRs for ER-negative disease from *BRCA1* carriers was also strong (ICC = 0.59). Correlations diminished when comparing HR estimates for ER-positive with estimates for ER-negative breast cancer; most of them were not significantly different from zero. This finding suggests that, to a large extent, the difference in SNP association patterns is due to mediating effects of tumor ER status. Under such a model, the effects of common breast cancer susceptibility variants and of *BRCA1* and *BRCA2* mutations on breast cancer risk would be multiplicative, after taking into account tumor ER status. As *BRCA1* carriers are more likely to develop ER-negative disease, SNPs associated with this subtype will be more informative in models to predict overall breast cancer risk in these women, whereas SNPs associated with ER-positive disease will be more useful in *BRCA2* carriers. Furthermore, ER-specific SNP associations could be used to provide separate estimates of ER-negative and ER-positive breast cancer risk for mutation carriers. This understanding allows the development of more refined models and is critical to the provision of accurate information to women considering more targeted preventive options.

However, the fact that the correlations between the HR estimates matched for ER status were smaller than 1 implies that there were still some differences in the associations after accounting for ER status. This could be due to sampling error or to real differences in genetic associations between *BRCA1* and *BRCA2* carriers and the general population. There are examples for such differences: *BRCA1* and *BRCA2* carrier-specific modifiers, such as the recently identified variant at 6p24, which was associated with breast cancer risk only in *BRCA2* carriers [6], and the ovarian cancer susceptibility locus 4q32, which appeared to modify ovarian cancer risk only for *BRCA1* carriers [7]. In addition, an ovarian cancer susceptibility locus 17q11.2 identified through population-based data has been shown to display a consistent association in *BRCA2* carriers, whereas an association of similar magnitude has been ruled out in *BRCA1* carriers [26]. The extent to which genetic susceptibility to breast cancer in mutation carriers and in the general population is shared, as well as the extent to which it is mediated by ER status, need to be quantified systematically by future studies.

We also assessed the associations of over 200,000 SNPs on the iCOGS array. We identified several variants not previously reported that were associated with breast cancer at *P* <10^−6^ in the analyses by PR status, HER status, TN phenotype, histological grade, nodal involvement, ductal and lobular morphologic subtypes. In the absence of *P*-values at genome-wide significance levels for these associations to account for multiple testing, these associations require confirmation through by gathering additional data.

Although this is the largest study of its kind, the statistical power to detect associations of variants conferring small effects with specific tumor characteristics may be low, owing to the limited data available. Future studies of additional *BRCA1* and *BRCA2* carriers with detailed tumor pathology information on new and previously recruited mutation carriers are needed. In this study, tumor pathology information was retrieved primarily from medical records. Despite extensive efforts, it is difficult to control the quality of these data. If there is low reproducibility in the classification of tumor characteristics for some samples, this could potentially add to the sampling error and make it more difficult to detect subtype-specific associations.

## Conclusions

We have identified additional genetic modifiers of breast cancer risk for mutation carriers among reported breast cancer susceptibility loci. Large differences in absolute risk are expected between mutation carriers who carry many and mutation carriers who carry few risk alleles of modifying variants [6],[7]. Therefore, in combination with previously identified modifiers, these variants may be of value for cancer risk prediction. Moreover, our results show that, to a large extent, the differences in breast cancer associations of known breast cancer susceptibility loci between *BRCA1* and *BRCA2* carriers and the general population are due to differences in the prevalence of tumor subtypes in *BRCA1* and *BRCA2* carriers. Estimates of the risks associated with these genetic variants based on large population-based association studies are likely to be applicable also to mutation carriers after taking ER status into account. Our results thus have implications for developing risk prediction models for breast cancer subtype-specific risks in mutation carriers that incorporate the effects of these SNPs.

## Additional file

## Electronic supplementary material

Additional file 1: Table S1.: Ethics committees that granted approval for the access and use of the data for this study. **Table S2.** Hormone receptor definitions used by the studies. **Table S3.** Associations of breast cancer susceptibility variants with breast cancer by tumor ER status after excluding prevalent cases. **Table S4.** Associations of breast cancer susceptibility variants with breast cancer by tumor PR status. **Table S5.** Associations of breast cancer susceptibility variants with breast cancer by tumor HER2 status. **Table S6.** Associations of breast cancer susceptibility variants with breast cancer by tumor triple-negative status. **Table S7.** Associations of breast cancer susceptibility variants with ductal breast cancer. **Table S8.** Associations of breast cancer susceptibility variants with lobular breast cancer. **Table S9.** Associations of breast cancer susceptibility variants with breast cancer by tumor grade. **Table S10.** Associations of breast cancer susceptibility variants with breast cancer by nodal involvement. **Figure S1.** QQ plots for SNP associations with ER-positive and -negative breast cancer in *BRCA1* carriers. **Figure S2.** QQ plots for SNP associations with ER-positive and -negative breast cancer in *BRCA2* carriers. **Figure S3.** Comparison of the breast cancer associations of breast cancer susceptibility loci in BCAC and in *BRCA1* and *BRCA2* carriers. **Figure S4.** Comparison of the associations of breast cancer susceptibility loci by ER status in *BRCA1* and in *BRCA2* carriers. **Figure S5.** Comparison of the associations of breast cancer susceptibility loci by ER status in population-based studies (BCAC) and in *BRCA1* carriers. **Figure S6.** Comparison of the associations of breast cancer susceptibility loci by ER status in population-based studies (BCAC) and in *BRCA2* carriers. (PDF 750 KB)

Below are the links to the authors’ original submitted files for images.

Authors’ original file for figure 1

## Abbreviations

BC: Breast cancer
BCAC: Breast Cancer Association Consortium
CIMBA: Consortium of Investigators of Modifiers of BRCA1/2
ER: Estrogen receptor
GWAS: Genome-wide association study
HER2: Human epidermal growth factor receptor 2
HR: Hazard ratio
ICC: Intraclass correlation
OR: Odds ratio
PR: Progesterone receptor
SNP: Single-nucleotide polymorphism
TN: Triple-negative
