# Supplementary material for: Associations of common breast cancer susceptibility alleles with risk of breast cancer subtypes in BRCA1 and BRCA2 mutation carriers
Source: Breast Cancer Res. 2014 Dec 31;16:3416. doi: 10.1186/s13058-014-0492-9 (PMC4406179; doi:10.1186/s13058-014-0492-9)
Supplement: Supplementary file 1 — Additional file 1: Table S1.: Ethics committees that granted approval for the access and use of the data for this study. Table S2. Hormone receptor definitions used by the studies. Table S3. Associations of breast cancer susceptibility variants with breast cancer by tumor ER status after excluding prevalent cases. Table S4. Associations of breast cancer susceptibility variants with breast cancer by tumor PR status. Table S5. Associations of breast cancer susceptibility variants with breast cancer by tumor HER2 status. Table S6. Associations of breast cancer susceptibility variants with breast cancer by tumor triple-negative status. Table S7. Associations of breast cancer susceptibility variants with ductal breast cancer. Table S8. Associations of breast cancer susceptibility variants with lobular breast cancer. Table S9. Associations of breast cancer susceptibility variants with breast cancer by tumor grade. Table S10. Associations of breast cancer susceptibility variants with breast cancer by nodal involvement. Figure S1. QQ plots for SNP associations with ER-positive and -negative breast cancer in BRCA1 carriers. Figure S2. QQ plots for SNP associations with ER-positive and -negative breast cancer in BRCA2 carriers. Figure S3. Comparison of the breast cancer associations of breast cancer susceptibility loci in BCAC and in BRCA1 and BRCA2 carriers. Figure S4. Comparison of the associations of breast cancer susceptibility loci by ER status in BRCA1 and in BRCA2 carriers. Figure S5. Comparison of the associations of breast cancer susceptibility loci by ER status in population-based studies (BCAC) and in BRCA1 carriers. Figure S6. Comparison of the associations of breast cancer susceptibility loci by ER status in population-based studies (BCAC) and in BRCA2 carriers. (PDF 750 KB) [file 13058_2014_492_MOESM1_ESM.pdf]

Additional file 1

contains supplementary material for

Associations of common breast cancer susceptibility alleles with risk of breast cancer subtypes in *BRCA1* and *BRCA2* mutation carriers

Karoline B Kuchenbaecker, Susan L Neuhausen, Mark Robson, Daniel Barrowdale, Lesley McGuffog, Anna Marie Mulligan, Irene L Andrulis, Amanda B Spurdle, Marjanka K Schmidt, Rita Katharina Schmutzler, Christoph Engel, Barbara Wappenschmidt, Heli Nevanlinna, Mads Thomassen, Melissa Southey, Paolo Radice, Susan J Ramus, Susan M Domchek, Katherine L Nathanson, Andrew Lee, Sue Healey, Robert L Nussbaum, Timothy R Rebbeck, Banu K Arun, Paul James, Beth Y Karlan, Jenny Lester, Ilana Cass, Breast Cancer Family Registry, Mary Beth Terry, Mary B Daly, David E Goldgar, Saundra S Buys, Ramunas Janavicius, Laima Tihomirova, Nadine Tung, Cecilia M Dorfling, Elizabeth J van Rensburg, Linda Steele, Thomas v O Hansen, Bent Ejlertsen, Anne-Marie Gerdes, Finn C Nielsen, Joe Dennis, Julie Cunningham, Steven Hart, Susan Slager, Ana Osorio, Javier Benitez, Mercedes Duran, Jeffrey N Weitzel, Isaac Tafur, Mary Hander, Paolo Peterlongo, Siranoush Manoukian, Bernard Peissel, Gaia Roversi, Giulietta Scuvera, Bernardo Bonanni, Paolo Mariani, Sara Volorio, Riccardo Dolcetti, Liliana Varesco, Laura Papi, Maria Grazia Tibiletti, Giuseppe Giannini, Florentia Fostira, Irene Konstantopoulou, Judy Garber, Ute Hamann, Alan Donaldson, Carole Brewer, Claire Foo, D Gareth Evans, Debra Frost, Diana Eccles, EMBRACE, Fiona Douglas, Angela Brady, Jackie Cook, Marc Tischkowitz, Julian Adlard, Julian Barwell, Kai-ren Ong, Lisa Walker, Louise Izatt, Lucy E Side, M John Kennedy, Mark T Rogers, Mary E Porteous, Patrick J Morrison, Radka Platte, Ros Eeles, Rosemarie Davidson, Shirley Hodgson, Steve Ellis, Andrew K Godwin, Kerstin Rhiem, Alfons Meindl, Nina Ditsch, Norbert Arnold, Hansjoerg Plendl, Dieter Niederacher, Christian Sutter, Doris Steinemann, Nadja Bogdanova-Markov, Karin Kast, Raymonda Varon-Mateeva, Shan Wang-Gohrke, Andrea Gehrig, Birgid Markiefka, Bruno Buecher, Cédrick Lefol, Dominique Stoppa-Lyonnet, Etienne Rouleau, Fabienne Prieur, Francesca Damiola, GEMO Study Collaborators, Laure Barjhoux, Laurence Faivre, Michel Longy, Nicolas Sevenet, Olga M Sinilnikova, Sylvie Mazoyer, Valérie Bonadona, Virginie Caux-Moncoutier, Claudine Isaacs, Tom Van Maerken, Kathleen Claes, Marion Piedmonte, Lesley Andrews, John Hays, Gustavo C Rodriguez, Trinidad Caldes, Miguel de la Hoya, Sofia Khan, Agnes Jager, Annemarie H van der Hout, Ans MW van den Ouweland, Antoinette Hollestelle, Frans BL Hogervorst, Cora M Aalfs, JL de Lange, Hanne EJ Meijers-Heijboer, Annemarie H van der Hout, Juul T Wijnen, KEP van Roozendaal, Arjen R Mensenkamp, Ans MW van den Ouweland, Carolien HM van Deurzen, Rob B van der Luijt, HEBON, Edith Olah, Orland Diez, Conxi Lazaro, Ignacio Blanco, Alex Teulé, Mireia Menendez, Anna Jakubowska, Jan Lubinski, Cezary Cybulski, Jacek Gronwald, Katarzyna Jaworska-Bieniek, Katarzyna Durda, Adalgeir Arason, Christine Maugard, Penny Soucy, Marco Montagna, Simona Agata, Manuel R Teixeira, KConFab Investigators, Curtis Olswold, Noralane Lindor, Vernon S Pankratz, Emily Hallberg, Xianshu Wang, Csilla I Szabo, Joseph Vijai, Lauren Jacobs, Marina Corines, Anne Lincoln, Andreas Berger, Anneliese Fink-Retter, Christian F Singer, Christine Rappaport, Daphne Geschwantler Kaulich, Georg Pfeiler, Muy-Kheng Tea, Catherine M Phelan, Phuong L Mai, Mark H Greene, Gad Rennert, Evgeny N Imyanitov, Gord Glendon, Amanda Ewart Toland, Anders Bojesen, Inge Sokilde Pedersen, Uffe Birk Jensen, Maria A Caligo, Eitan Friedman, Raanan Berger, Yael Laitman, Johanna Rantala, Brita Arver, Niklas Loman, Ake Borg, Hans Ehrencrona, Olufunmilayo I Olopade, Jacques Simard, Douglas F Easton, Georgia Chenevix-Trench, Kenneth Offit, Fergus J Couch, Antonis C Antoniou, On behalf of CIMBA

This PDF file includes:

**Table S1** Ethics committees that granted approval for the access and use of the data for this study	page 3
**Table S2** Hormone receptor definitions used by the studies	page 6
**Table S3** Associations of breast cancer susceptibility variants with breast cancer by tumor ER-status after excluding prevalent cases	page 8
**Table S4** Associations of breast cancer susceptibility variants with breast cancer by tumor PR-status	page 9
**Table S5** Associations of breast cancer susceptibility variants with breast cancer by tumor HER2-status	page 12
**Table S6** Associations of breast cancer susceptibility variants with breast cancer by tumor triple negative status	page 15
**Table S7** Associations of breast cancer susceptibility variants with ductal breast	page 18
**Table S8** Associations of breast cancer susceptibility variants with lobular breast cancer	page 21
**Table S9** Associations of breast cancer susceptibility variants with breast cancer by tumor grade	page 24
**Table S10** Associations of breast cancer susceptibility variants with breast cancer by nodal involvement	page 27
**Figure legends**	page 30
**Figure S1** QQ-plots for SNP associations with ER-positive and –negative breast cancer in *BRCA1* carriers	page 31
**Figure S2** QQ-plots for SNP associations with ER-positive and –negative breast cancer in *BRCA2* carriers	page 32
**Figure S3** Comparison of the breast cancer associations of breast cancer susceptibility loci in BCAC, *BRCA1* and *BRCA2* carriers	page 33
**Figure S4** Comparison of the associations of breast cancer susceptibility loci by ER-status in *BRCA1* and in *BRCA2* carriers	page 34
**Figure S5** Comparison of the associations of breast cancer susceptibility loci by ER-status in population-based studies (BCAC) and in *BRCA1* carriers	page 35
**Figure S6** Comparison of the associations of breast cancer susceptibility loci by ER-status in population-based studies (BCAC) and in *BRCA2* carriers	page 36

Table S1 Ethics committees that granted approval for the access and use of the data for this study

**Study**	**Country**	**Committee approval**
Breast Cancer Family Registry (BCFR)	USA	Institutional Review Board University of Utah
(BCFR - addtional)	Australia	The University of Melbnourne Health Sciences Human Ethics Sub-Committee
(BCFR - addtional)	USA	Columbia University Medical Center Institutional Review Board
(BCFR - addtional)	USA	Northern Californa Cancer Center Institutional Review Board
(BCFR - addtional)	Canada	University Health Network Research Ethics Board
(BCFR - addtional)	Canada	Mount Sinai Hospital Research Ethics Board
Baltic Familial Breast and Ovarian Cancer Consortium (BFBOCC)	Latvia,Lithuania	Centrālā medicīnas ētikas Komiteja
BRCA-gene mutations and breast cancer in South African women (BMBSA)	South Africa	Univ. of Pretoria and Pretoria Academic Hospitals Ethics Committee
Beckman Research Institute of the City of Hope (BRICOH)	USA	UC Irvine: Office of Research Administration Institutional Review Board
Copenhagen Breast Cancer Study (CBCS)	Denmark	De Videnskabsetiske Komiteer I Region Hovedsladen
Spanish National Cancer Centre (CNIO)	Spain	Instituto de Salud Carlos III Comité de Bioética y Bienestar Animal
CNIO- additional	Greece	Bioethics committee of NCSR ''Demokritos''
CONsorzio Studi ITaliani sui Tumori Ereditari Alla Mammella (CONSIT TEAM)	Italy	Comitato Etico Indipendente della Fondazione IRCCS "Istituto Nazionale dei Tumori"
Deutsches Krebsforschungszentrum (DKFZ)	Germany	Ethik-Kommission des Klinikums der Universität
(DKFZ - addtional)	Columbia	Hospital Universitario de San Ignacio Comité de Investigaciones y Etica
(DKFZ - addtional)	Pakistan	Shaukat Khanum Memorial Cancer Hospital and Research Centre Institutional Review Board
HEreditary Breast and Ovarian study Netherlands (HEBON)	The Netherlands	Protocol Toetsingscommissie van het Nederlands Kanker Instituut/Antoni van Leeuwenhoek Ziekenhuis
Epidemiological study of BRCA1 and BRCA2 mutation carriers (EMBRACE)	UK and EIRE	Anglia & Oxford MREC
Fox Chase Cancer Center (FCCC)	USA	Institutional Review Board Fox Chase Cancer Center
German Consortium of Hereditary Breast and Ovarian Cancer (GC-HBOC)	Germany	Ethik-Kommission der Medizinischen Fakultät der Universät zu Köln
Georgetown University (GEORGETOWN)	USA	MedStar Research Institute - Georgetown University Oncology Institutional Review Board
Genetic Modifiers of cancer risk in *BRCA1*/2 mutation carriers (GEMO)	France	Comité consultatif sur le traitement de l'information en matière de recherche dans le domaine de la santé
Gynecologic Oncology Group (GOG)	USA	National Cancer Institute - Cancer Prevention and Control Concept Review Committee
Hospital Clinico San Carlos (HCSC)	Spain	Comité Ético de Investigación Clínia Hospital Clínico San Carlos

Helsinki Breast Cancer Study (HEBCS)	Finland	Helsingin ja uudenmaan sairaanhoitopiiri (Helsinki University Central Hospital ethics committee)
Hungarian Breast and Ovarian Cancer Study (HUNBOCS)	Hungary	Institutional Review Board of the Hungarian National Institute of Oncology
Univeristy Hospital Vall d'Hebron (HVH)	Spain	The Hospital Universitario Vall d'Hebron Clinical Research Ethics Committee
Institut Català d'Oncologia (ICO)	Spain	Catalan Institute of Oncology Institutional Review Board
Iceland Landspitali - University Hospital (ILUH)	Iceland	Vísindasiđanefnd National Boethics Committee
Interdisciplinary Health Research International Team Breast Cancer Susceptibility (INHERIT)	Quebec - Canada	Comité d'éthique de la recherche du Centre Hospitalier Universitaire de Québec
Istituto Oncologico Veneto Hereditary Breast and Ovarian Cancer Study (IOVHBOCS)	Italy	Centro Oncologico Regionale Azienda Ospedale Di Padova Comitato Etico
Kathleen Cuningham Foundation Consortium for Research into Familial Breast Cancer (KCONFAB)	Australia	Peter MacCallum Cancer Centre Ethics Committee
(KCONFAB - additional)	Australia	Queensland Institute of Medical Research - Human Research Ethics Committee
Modifiers and Genetics in Cancer (MAGIC)	USA	University of Pennsylvania Institutional Review Board
Mayo Clinic (MAYO)	USA	Mayo Clinic Institutional Review Boards
McGill University (MCGILL)	Canada	McGill Faculty of Medicine Institutional Review Board
Memorial Sloane Kettering Cancer Center (MSKCC)	USA	Memorial Sloan-Kettering Cancer Center IRB
(MSKCC - additional)	USA	Human Biospecimen Utilization Committee
Modifier Study of Quantitative Effects on Disease (MOD-SQUAD)	USA	Mayo Clinic Institutional Review Boards
General Hospital Vienna (MUV)	Austria	Ethikkommission der Medizinischen Universität Wien
National Cancer Institute (NCI)	USA	NIH Ethics Office
National Israeli Cancer Control Center (NICCC)	Israel	Carmel Medical Center Institutional Review Board (Helsinki Committee)
N.N. Petrov Institute of Oncology (NNPIO)	Russia	N.N. Petrov Institional Ethical Committee
Ontario Cancer Genetics Network (OCGN)	Canada	Mount Sinai Hospital Research Ethics Board
The Ohio State University Comprehensive Cancer Centre (OSU-CCG)	USA	Cancer Institutional Review Board
Odense University Hospital (OUH)	Denmark	Den Videnskabsetiske Komité for Region Syddanmark
Pisa Breast Cancer Study (PBCS)	Italy	Comitato Etico per lo studio del farmaco sull'uomo
Swedish Breast Cancer Study (SWE-BRCA)	Sweden	Regionala Etikprövningsnämnden Stockholm
University of California Irvine (UCI)	USA	UC Irvine: Office of Research Administration Institutional Review Board
University of California Los Angeles (UCLA)	USA	UCLA Institutional Review Board
University of California San Francisco	USA	Committee on Human Research

(UCSF)		
UK and Gilda Radner Familial Ovarian Cancer Registries (UKGRFOCR)	UK	Cambridge Local Research Ethics Committee
(UKGRFOCR - additional)	USA	Roswell Park Cancer Institute IRB
University of Pennsylvania (UPENN)	USA	University of Pennsylvania Institutional Review Board
Women's Cancer Research Institute (WCRI)	USA	Cedars-Sinai Institutional Review Board

Table S2 Hormone receptor definitions used by the studies

Study	ER/PR definition	HER2 definition
BCFR-AU	As given by medical records	As given by medical records
BCFR-NC	0: <1%, 1: ≥1% stained nuclei	0: 0/1+/2+ (non-amplified), 1: 3+ or 2+ with FISH amplification
BCFR-NY	As given by medical records	As given by medical records
BCFR-ON	As given by medical records	As given by medical records
BCFR-PA	As given by medical records	As given by medical records
BCFR-UT	As given by medical records	As given by medical records
BFBOCC	0: <10%, 1: ≥10% stained nuclei	0: 0/1+/2+ (non-amplified), 1: 3+ or 2+ with FISH amplification
BIDMC	As given by medical records	As given by medical records
BRICOH	As given by medical records	As given by medical records
CBCS	0: <10%, 1: ≥10% stained nuclei	0: 0/1+/2+ (non-amplified), 1: 3+ or 2+ with FISH amplification
CNIO	0: <10%, 1: ≥10% stained nuclei	As given by medical records
COH	As given by medical records	As given by medical records
CONSIT TEAM	0: <10%, 1: ≥10% stained nuclei<br>0: Allred≤2; 1: Allred>2<br>0: <=10fmol/mg, 1: >10fmol/mg	0: 0/1+, 1: 2+/3+<br>0: <10%, 1: ≥10% stained nuclei
DEMOKRITOS	0: <1%, 1: ≥1% stained nuclei<br>0: <10%, 1: ≥10% stained nuclei	As given by medical records
DFCI	As given by medical records	0: 0/1+/2+ (non-amplified), 1: 3+ or 2+ with FISH amplification
DKFZ	0: <10%, 1: ≥10% stained nuclei	0: 0/1+/2+ (non-amplified), 1: 3+ or 2+ with FISH amplification
DKFZ	0: Immunoreactive Score 0, 1: IRS≥1	
EMBRACE	As given by medical records	As given by medical records
FCCC	As given by medical records	As given by medical records
GC-HBOC	0: Remmele-Score=0, 1: Remmele≥1	0: 0/1+/2+ (non-amplified), 1: 3+ or 2+ with FISH amplification
GEMO	0: <10%, 1: ≥10% stained nuclei	0: 0/1+/2+ (non-amplified), 1: 3+ or 2+ with FISH amplification
GEORGETOWN	0: <10%, 1: ≥10% stained nuclei	0: 0/1+/2+ (non-amplified), 1: 3+ or 2+ with FISH amplification
G-FAST	As given by medical records	As given by medical records
HCSC	0: Allred≤2; 1: Allred>2	0: 0/1+/2+ (non-amplified), 1: 3+ or 2+ with FISH amplification
HEBCS	0: <10%, 1: ≥10% stained nuclei	0: <6 CISH HER2 copy numbers, 1: ≥6 CISH replications
HEBON	As given by medical records	As given by medical records
HRBCP	As given by medical records	As given by medical records
ICO	As given by medical records	As given by medical records
IHCC	0: <1%, 1: ≥1% stained nuclei	0: 0/1+/2+ (non-amplified), 1: 3+ or 2+ with FISH amplification
ILUH	0: Immunoreactive Score 0, 1: IRS≥1	0: 0/1+/2+ (non-amplified), 1: 3+ or 2+ with FISH amplification

INHERIT	As given by medical records	As given by medical records
IOVHBOCS	As given by medical records	As given by medical records
IPOBCS	0: <1%, 1: ≥1% stained nuclei	0: 0/1+/2+ (non-amplified), 1: 3+ or 2+ with FISH amplification
KCONFAB	0: Allred≤2; 1: Allred>2	0: 0/1+/2+, 1: 3+
KOHBRA	As given by medical records	As given by medical records
MAGIC	As given by medical records	0: 0/1+/2+ (non-amplified), 1: 3+ or 2+ with FISH amplification
MAYO	As given by medical records	0: 0/1+/2+ (non-amplified), 1: 3+ or 2+ with FISH amplification
MODSQUAD	As given by medical records	As given by medical records
MSKCC	As given by medical records	As given by medical records
MUV	0: <10%, 1: ≥10% stained nuclei	As given by medical records
MUV	0: Immunoreactive Score 0, 1: IRS≥1	
NCI	As given by medical records	As given by medical records
NNPIO	As given by medical records	As given by medical records
OCGN	As given by medical records	As given by medical records
OSU CCG	As given by medical records	As given by medical records
OUH	0: <10%, 1: ≥10% stained nuclei	0: 0/1+/2+ (non-amplified), 1: 3+ or amplified by FISH
PBCS	0: <1%, 1: ≥1% stained nuclei	0: 0/1+, 1: 2+/3+
SEABASS	0: <10%, 1: ≥10% stained nuclei	0: 0/1+, 1: 2+/3+
SWE-BRCA	0: <10%, 1: ≥10% stained nuclei	0: 0/1+/2+ (non-amplified), 1: 3+ or 2+ with FISH amplification
UCHICAGO	0: <10%, 1: ≥10% stained nuclei	As given by medical records
UCSF	As given by medical records	As given by medical records
UKGRFOCR	As given by medical records	As given by medical records
UPENN	As given by medical records	As given by medical records
WCP	0: <5%, 1: ≥5% stained nuclei	As given by medical records

Notes: Allred: cell proportion score (0-5) + staining intensity score (0-3); Remmele & Immunoreactive: cell proportion score (0-4) x staining intensity score (0-3); FISH = fluorescence in-situ hybridization

Table S3 Associations of previously reported breast cancer susceptibility variants with breast cancer by tumor ER-status in *BRCA1* and *BRCA2* carriers after excluding prevalent breast cancer cases. Restricted to variants with p<0.01 with ER-positive or ER-negative disease in the analyses using the complete data. Estimates based on the entire sample are also given for comparison.

SNP (locus)	Sample	HR for ER-positive (95%CI)	HR for ER-negative (95%CI)
***BRCA1* carriers**			
rs4245739 (1q32.1)	all	1.02 (0.91-1.13)	1.12 (1.07-1.19)
	no prevalent cases	1.07 (0.94-1.22)	1.08 (1.02-1.16)
rs13387042 (2q35)	all	0.86 (0.78-0.95)	1.02 (0.97-1.07)
	no prevalent cases	0.85 (0.76-0.96)	1.00 (0.95-1.07)
rs10069690 (5p15.33)	all	1.09 (0.98-1.22)	1.24 (1.18-1.31)
	no prevalent cases	1.05 (0.92-1.19)	1.21 (1.14-1.30)
rs3757318 (6q25.1)	all	1.06 (0.89-1.26)	1.24 (1.14-1.35)
	no prevalent cases	1.17 (0.95-1.43)	1.19 (1.07-1.32)
rs7904519 (10q25.2)	all	1.12 (1.02-1.23)	1.09 (1.04-1.14)
	no prevalent cases	1.10 (0.99-1.24)	1.08 (1.02-1.15)
rs2981579 (10q26.12)	all	1.29 (1.17-1.43)	0.92 (0.87-0.96)
	no prevalent cases	1.25 (1.11-1.41)	0.94 (0.88-0.99)
rs3817198 (11p15.5)	all	1.07 (0.97-1.18)	1.08 (1.03-1.14)
	no prevalent cases	1.07 (0.95-1.20)	1.06 (1.00-1.13)
rs10771399 (12p11.22)	all	0.91 (0.78-1.06)	0.83 (0.77-0.90)
	no prevalent cases	0.92 (0.76-1.11)	0.83 (0.75-0.92)
rs3803662 (16q12.1)	all	1.22 (1.10-1.35)	1.01 (0.96-1.07)
	no prevalent cases	1.27 (1.12-1.44)	1.01 (0.94-1.08)
rs8170 (19p13.11)	all	1.06 (0.95-1.20)	1.22 (1.15-1.30)
	no prevalent cases	1.14 (0.98-1.31)	1.21 (1.13-1.31)
***BRCA2* carriers**			
rs10069690 (5p15.33)	all	1.08 (1.00-1.17)	1.25 (1.08-1.44)
	no prevalent cases	1.04 (0.94-1.14)	1.30 (1.08-1.56)
rs9348512 (6q24.3)	all	0.86 (0.80-0.92)	0.79 (0.69-0.91)
	no prevalent cases	0.85 (0.78-0.93)	0.78 (0.65-0.93)
rs3757318 (6q25.1)	all	1.09 (0.96-1.24)	1.33 (1.07-1.65)
	no prevalent cases	0.98 (0.83-1.14)	1.45 (1.11-1.91)
rs2981579 (10q26.12)	all	1.29 (1.21-1.38)	1.05 (0.92-1.20)
	no prevalent cases	1.29 (1.19-1.40)	1.09 (0.92-1.28)
rs3817198 (11p15.5)	all	1.15 (1.07-1.24)	0.98 (0.85-1.13)
	no prevalent cases	1.10 (1.01-1.20)	1.04 (0.87-1.24)
rs554219 (11q13.3)	all	1.15 (1.04-1.26)	0.90 (0.73-1.10)
	no prevalent cases	1.14 (1.02-1.28)	0.86 (0.66-1.13)
rs10771399 (12p11.22)	all	0.94 (0.84-1.05)	0.72 (0.56-0.91)
	no prevalent cases	0.92 (0.80-1.05)	0.67 (0.49-0.90)
rs1292011 (12q24.21)	all	0.88 (0.82-0.95)	1.07 (0.94-1.23)
	no prevalent cases	0.93 (0.85-1.01)	1.14 (0.96-1.36)
rs3803662 (16q12.1)	all	1.27 (1.18-1.36)	1.12 (0.97-1.30)
	no prevalent cases	1.26 (1.15-1.37)	1.14 (0.95-1.36)

Table S4 Associations of 74 previously reported breast cancer susceptibility variants with breast cancer by tumor PR-status in *BRCA1* and *BRCA2* carriers. Associations with p<0.01 in bold.

					***BRCA1* carriers**					***BRCA2* carriers**				
					**PR-negative**		**PR-positive**			**PR-negative**		**PR-positive**		
**Locus**	**SNP**	**Position**	**Ref#**	**Eff#**	**HR (95%CI)**	**p**	**HR (95%CI)**	**p**	**p_{het}**	**HR (95%CI)**	**p**	**HR (95%CI)**	**p**	**p_{het}**
1p36.22	rs616488	10566215	A	G	0.95 (0.91-1.00)	0.08	1.00 (0.89-1.11)	0.95	0.51	0.96 (0.86-1.08)	0.54	0.99 (0.91-1.07)	0.78	0.75
1p13.2	rs12022378	114448389	G	A	1.04 (0.97-1.11)	0.24	1.01 (0.87-1.16)	0.93	0.71	1.02 (0.87-1.18)	0.82	1.04 (0.94-1.15)	0.43	0.80
1p11.2	rs11249433	121280613	A	G	0.99 (0.94-1.04)	0.59	1.02 (0.92-1.14)	0.68	0.58	1.14 (1.02-1.27)	0.02	1.01 (0.93-1.09)	0.80	0.11
1q32.1_1	rs6678914	202187176	G	A	0.96 (0.91-1.00)	0.08	1.08 (0.97-1.20)	0.14	0.05	0.95 (0.84-1.06)	0.35	1.09 (1.01-1.18)	0.02	0.06
1q32.1_2	rs4245739	204518842	A	C	1.10 (1.04-1.16)	**$3.2x10^{-4}$**	1.09 (0.97-1.22)	0.16	0.82	1.07 (0.95-1.20)	0.28	0.92 (0.85-1.01)	0.07	0.07
2p24.1	rs12710696	19320803	G	A	1.00 (0.95-1.05)	0.91	1.06 (0.95-1.18)	0.30	0.40	1.02 (0.92-1.14)	0.66	0.99 (0.91-1.07)	0.76	0.61
2q14.2	rs4849887	121245122	G	A	1.02 (0.94-1.10)	0.67	1.04 (0.88-1.23)	0.66	0.83	0.83 (0.68-1.01)	0.06	1.06 (0.94-1.20)	0.32	0.05
2q31.1	rs2016394	172972971	G	A	1.02 (0.97-1.07)	0.53	1.00 (0.90-1.12)	0.96	0.84	1.04 (0.93-1.16)	0.46	0.96 (0.89-1.04)	0.34	0.28
2q31.1	rs1550623	174212894	A	G	1.02 (0.95-1.09)	0.58	0.98 (0.84-1.14)	0.78	0.65	0.99 (0.86-1.15)	0.89	0.96 (0.87-1.07)	0.45	0.74
2q35	rs13387042	217905832	A	G	1.03 (0.98-1.08)	0.23	0.82 (0.74-0.91)	**$2.3x10^{-4}$**	**$2.5x10^{-4}$**	1.03 (0.93-1.15)	0.54	0.96 (0.90-1.04)	0.34	0.31
2q35	rs16857609	218296508	G	A	1.03 (0.97-1.09)	0.31	1.11 (0.99-1.25)	0.09	0.29	0.96 (0.84-1.09)	0.53	0.96 (0.89-1.05)	0.41	0.95
3p26.1	rs6762644	4742276	A	G	1.02 (0.97-1.07)	0.44	1.11 (0.99-1.23)	0.07	0.22	1.04 (0.93-1.17)	0.48	0.95 (0.88-1.03)	0.26	0.25
3p24.1	rs4973768	27416013	G	A	1.01 (0.96-1.06)	0.76	1.04 (0.94-1.16)	0.39	0.54	1.07 (0.95-1.19)	0.26	1.10 (1.01-1.18)	0.02	0.71
3p24.1	rs12493607	30682939	C	G	0.98 (0.93-1.03)	0.51	1.03 (0.92-1.15)	0.61	0.48	0.98 (0.87-1.1)	0.73	0.98 (0.90-1.06)	0.64	0.99
4q24	rs9790517	106084778	G	A	0.99 (0.94-1.05)	0.73	0.96 (0.85-1.08)	0.51	0.67	1.02 (0.9-1.17)	0.71	0.95 (0.86-1.04)	0.27	0.37
4q34.1	rs6828523	175846426	C	A	1.03 (0.96-1.12)	0.39	1.00 (0.85-1.19)	0.97	0.76	1.05 (0.88-1.25)	0.58	0.95 (0.83-1.08)	0.41	0.38
5p15.33	rs10069690	1279790	G	A	1.24 (1.17-1.30)	**$1.3x10^{-14}$**	1.10 (0.98-1.24)	0.09	0.11	1.16 (1.02-1.31)	0.02	1.09 (1.00-1.19)	0.05	0.46
5p15.33	rs7725218	1282414	G	A	1.09 (1.04-1.15)	**$5.0x10^{-4}$**	1.03 (0.93-1.15)	0.53	0.41	1.04 (0.93-1.17)	0.49	1.06 (0.98-1.15)	0.14	0.79
5p15.33	rs2736108	1297488	G	A	0.87 (0.82-0.92)	**$3.9x10^{-7}$**	0.97 (0.86-1.08)	0.55	0.12	0.90 (0.79-1.02)	0.09	0.95 (0.88-1.04)	0.28	0.46
5p12	rs10941679	44706498	A	G	0.96 (0.91-1.02)	0.21	1.08 (0.95-1.22)	0.23	0.13	0.99 (0.87-1.13)	0.91	1.12 (1.03-1.22)	**$9.3x10^{-3}$**	0.14
5q11.2	rs889312	56031884	A	C	0.99 (0.94-1.05)	0.79	1.10 (0.98-1.24)	0.10	0.13	0.98 (0.87-1.1)	0.73	1.08 (0.99-1.17)	0.09	0.23
5q11.3	rs10472076	58184061	A	G	0.99 (0.94-1.04)	0.58	1.08 (0.97-1.20)	0.15	0.15	0.99 (0.88-1.11)	0.86	0.99 (0.92-1.08)	0.85	0.97
5q11.3	rs1353747	58337481	A	C	0.95 (0.88-1.04)	0.27	1.07 (0.9-1.27)	0.45	0.28	0.94 (0.77-1.14)	0.52	0.96 (0.84-1.10)	0.56	0.85
5q33.3	rs1432679	158244083	A	G	1.04 (0.99-1.09)	0.14	1.08 (0.98-1.20)	0.13	0.48	1.00 (0.9-1.11)	0.98	1.01 (0.94-1.09)	0.73	0.84
6p25.3	rs11242675	1318878	A	G	0.94 (0.89-0.99)	0.01	1.05 (0.94-1.17)	0.43	0.10	0.97 (0.86-1.09)	0.59	1.03 (0.95-1.12)	0.47	0.43

6p24.3	rs9348512	10456706	C	A	1.00 (0.95-1.05)	0.92	0.99 (0.89-1.11)	0.88	0.93	0.84 (0.75-0.94)	**2.8x10^{-3}**	0.85 (0.78-0.92)	**3.8x10^{-5}**	0.96
6p23	rs204247	13722523	A	G	0.99 (0.94-1.04)	0.69	1.04 (0.94-1.15)	0.50	0.45	1.07 (0.96-1.2)	0.23	1.10 (1.02-1.19)	0.01	0.71
6q14	rs17530068	82193109	A	G	1.03 (0.97-1.09)	0.30	1.03 (0.91-1.16)	0.66	0.97	0.98 (0.86-1.12)	0.79	1.16 (1.07-1.27)	**6.6x10^{-4}**	0.05
6q25.1	rs3757318	151914113	G	A	1.22 (1.12-1.33)	**2.5x10^{-6}**	1.10 (0.91-1.33)	0.31	0.34	1.19 (0.98-1.45)	0.08	1.12 (0.97-1.29)	0.11	0.63
6q25.1	rs2046210	151948366	G	A	1.18 (1.13-1.24)	**2.0x10^{-11}**	1.09 (0.98-1.21)	0.12	0.19	1.14 (1.01-1.28)	0.03	1.01 (0.94-1.10)	0.73	0.14
7q35	rs720475	144074929	G	A	0.98 (0.92-1.03)	0.42	0.98 (0.87-1.11)	0.78	0.95	0.93 (0.82-1.06)	0.28	1.02 (0.93-1.11)	0.71	0.29
8p12	rs9693444	29509616	C	A	0.99 (0.94-1.04)	0.63	1.08 (0.97-1.20)	0.18	0.18	1.00 (0.89-1.12)	0.95	0.98 (0.90-1.06)	0.58	0.81
8q21.11	rs6472903	76230301	A	C	1.02 (0.96-1.09)	0.51	0.96 (0.84-1.11)	0.59	0.47	0.97 (0.84-1.13)	0.73	0.97 (0.87-1.08)	0.58	0.98
8q21.11	rs2943559	76417937	A	G	1.07 (0.99-1.17)	0.10	1.01 (0.83-1.22)	0.95	0.57	1.11 (0.92-1.34)	0.29	1.10 (0.97-1.25)	0.15	0.95
8q24.21	rs11780156	129194641	G	A	0.98 (0.92-1.04)	0.44	0.85 (0.74-0.97)	0.02	0.08	0.99 (0.86-1.13)	0.89	0.96 (0.87-1.06)	0.43	0.76
8q24.21	rs13281615	128355618	A	G	1.00 (0.96-1.06)	0.83	1.06 (0.96-1.18)	0.27	0.39	1.01 (0.91-1.13)	0.80	1.04 (0.96-1.12)	0.35	0.76
9p21.3	rs1011970	22062134	C	A	1.05 (0.99-1.12)	0.11	0.90 (0.77-1.04)	0.16	0.07	1.06 (0.92-1.22)	0.44	1.01 (0.92-1.12)	0.82	0.64
9q31.2	rs10759243	110306115	C	A	0.97 (0.92-1.02)	0.27	1.02 (0.91-1.14)	0.74	0.47	0.98 (0.86-1.11)	0.70	1.01 (0.93-1.10)	0.83	0.69
9q31.2	rs865686	110888478	A	C	1.01 (0.96-1.07)	0.56	0.92 (0.82-1.03)	0.13	0.13	1.04 (0.92-1.17)	0.53	0.97 (0.89-1.05)	0.39	0.34
10p12.31	rs7072776	22032942	G	A	0.96 (0.91-1.01)	0.16	1.07 (0.96-1.20)	0.20	0.10	0.92 (0.81-1.03)	0.14	1.02 (0.94-1.11)	0.57	0.13
10p12.31	rs11814448	22315843	A	C	1.17 (1.00-1.36)	0.05	1.02 (0.71-1.46)	0.91	0.52					
10q21.2	rs10995190	64278682	G	A	1.02 (0.96-1.09)	0.53	0.87 (0.75-1.01)	0.06	0.06	0.89 (0.76-1.04)	0.16	0.97 (0.87-1.08)	0.56	0.42
10q22.3	rs704010	80841148	G	A	0.99 (0.94-1.04)	0.56	1.14 (1.02-1.26)	0.02	0.02	0.96 (0.86-1.07)	0.46	1.03 (0.95-1.11)	0.44	0.31
10q25.2	rs7904519	114773927	A	G	1.09 (1.04-1.15)	**3.0x10^{-4}**	1.10 (1.00-1.22)	0.06	0.88	0.92 (0.82-1.02)	0.13	1.07 (1.00-1.16)	0.06	0.03
10q26.12	rs2981579	123337335	G	A	0.92 (0.88-0.97)	**8.8x10^{-4}**	1.33 (1.20-1.48)	**1.3x10^{-7}**	**5.2x10^{-9}**	1.13 (1.01-1.26)	0.03	1.29 (1.20-1.40)	**5.7x10^{-11}**	0.06
10q26.12	rs11199914	123093901	G	A	1.03 (0.98-1.09)	0.21	0.99 (0.89-1.11)	0.90	0.55	0.91 (0.81-1.03)	0.14	0.98 (0.90-1.06)	0.55	0.40
11p15.5	rs3817198	1909006	A	G	1.07 (1.02-1.13)	**8.6x10^{-3}**	1.11 (0.99-1.24)	0.07	0.59	1.07 (0.95-1.21)	0.23	1.13 (1.04-1.23)	**2.7x10^{-3}**	0.49
11q13.1	rs3903072	65583066	C	A	1.00 (0.95-1.05)	0.95	0.96 (0.86-1.06)	0.39	0.45	0.92 (0.82-1.02)	0.12	1.00 (0.93-1.08)	0.98	0.21
11q13.3	rs554219	69331642	C	G	0.99 (0.92-1.07)	0.89	1.16 (1.00-1.35)	0.05	0.09	1.01 (0.85-1.19)	0.94	1.14 (1.02-1.27)	0.02	0.25
11q13.3	c11_pos69088342	69379161	C	A	0.98 (0.88-1.09)	0.70	1.25 (1.01-1.54)	0.04	0.06	0.97 (0.77-1.23)	0.81	1.12 (0.96-1.31)	0.15	0.34
11q13.3	rs494406	69344241	G	A	1.00 (0.95-1.06)	0.89	1.07 (0.95-1.21)	0.24	0.34	1.06 (0.94-1.20)	0.31	1.04 (0.96-1.14)	0.34	0.79
11q24.3	rs11820646	129461171	G	A	0.93 (0.89-0.98)	**6.0x10^{-3}**	0.91 (0.82-1.02)	0.10	0.76	0.87 (0.77-0.97)	0.01	0.94 (0.87-1.02)	0.14	0.27
12p13.1	rs12422552	14413931	G	C	1.04 (0.98-1.09)	0.19	0.92 (0.81-1.04)	0.17	0.10	1.02 (0.90-1.16)	0.72	0.98 (0.90-1.07)	0.69	0.62
12p11.22	rs10771399	28155080	A	G	0.86 (0.79-0.93)	**1.6x10^{-4}**	0.82 (0.69-0.99)	0.04	0.72	0.86 (0.72-1.03)	0.10	0.90 (0.79-1.03)	0.12	0.68

12q22	rs17356907	96027759	A	G	0.96 (0.91-1.01)	0.12	0.93 (0.82-1.05)	0.22	0.65	0.93 (0.82-1.05)	0.23	1.02 (0.94-1.11)	0.56	0.21
12q24.21	rs1292011	115836522	A	G	1.04 (0.99-1.09)	0.12	0.88 (0.79-0.98)	0.02	0.01	0.95 (0.85-1.06)	0.34	0.91 (0.84-0.99)	0.02	0.59
13q13.1	rs11571833	32972626	T	A	1.01 (0.77-1.31)	0.96	1.09 (0.64-1.84)	0.75	0.81	0.99 (0.70-1.39)	0.93	0.94 (0.72-1.21)	0.62	0.82
14q13.3	rs2236007	37132769	G	A	0.96 (0.91-1.02)	0.22	0.98 (0.86-1.12)	0.78	0.81	1.17 (1.03-1.33)	0.02	0.90 (0.82-1.00)	0.04	**3.9x10^{-3}**
14q24.1	rs2588809	68660428	G	A	0.93 (0.87-0.99)	0.02	1.05 (0.93-1.20)	0.41	0.09	1.01 (0.87-1.17)	0.89	1.01 (0.91-1.11)	0.87	0.98
14q24.1	rs999737	69034682	G	A	0.99 (0.94-1.05)	0.77	0.83 (0.73-0.96)	**9.3x10^{-3}**	0.03	0.95 (0.83-1.08)	0.42	0.99 (0.90-1.08)	0.82	0.61
14q32.11	rs941764	91841069	A	G	1.02 (0.97-1.07)	0.48	1.06 (0.94-1.19)	0.35	0.59	1.03 (0.92-1.15)	0.65	1.03 (0.95-1.11)	0.49	0.97
16q12.1_1	rs3803662	52586341	G	A	1.03 (0.97-1.08)	0.33	1.18 (1.06-1.32)	**3.7x10^{-3}**	0.03	1.15 (1.02-1.29)	0.02	1.28 (1.18-1.39)	**2.3x10^{-9}**	0.15
16q12.1_2	rs11075995	53855291	A	T	1.00 (0.95-1.06)	0.98	1.06 (0.94-1.19)	0.35	0.42	1.01 (0.89-1.16)	0.84	1.02 (0.93-1.12)	0.63	0.93
16q12.1_2	rs17817449	53813367	A	C	0.93 (0.89-0.98)	**4.1x10^{-3}**	1.03 (0.93-1.15)	0.53	0.09	1.00 (0.89-1.12)	0.97	0.91 (0.84-0.98)	0.02	0.21
16q23.2	rs13329835	80650805	A	G	1.06 (1.00-1.12)	0.06	0.99 (0.87-1.12)	0.90	0.40	1.03 (0.91-1.16)	0.64	1.03 (0.95-1.13)	0.43	0.94
17q22	rs6504950	53056471	G	A	1.00 (0.94-1.05)	0.86	0.94 (0.83-1.06)	0.30	0.40	1.03 (0.91-1.16)	0.64	1.05 (0.96-1.14)	0.29	0.82
18q11.2	rs527616	24337424	C	G	0.97 (0.92-1.02)	0.27	1.05 (0.95-1.18)	0.33	0.20	0.97 (0.87-1.08)	0.58	0.96 (0.88-1.03)	0.25	0.84
18q11.2	rs1436904	22824665	A	C	1.00 (0.95-1.05)	1.00	0.96 (0.86-1.07)	0.43	0.49	0.97 (0.87-1.08)	0.55	0.95 (0.88-1.03)	0.19	0.79
19p13.11	rs8170	17389704	G	A	1.20 (1.13-1.28)	**4.6x10^{-9}**	1.12 (0.99-1.28)	0.08	0.38	0.90 (0.78-1.04)	0.17	1.02 (0.93-1.13)	0.66	0.19
19p13.11	rs4808801	18571141	A	G	0.98 (0.93-1.03)	0.44	0.99 (0.89-1.10)	0.81	0.91	0.96 (0.86-1.08)	0.50	0.97 (0.9-1.06)	0.51	0.87
19q13.31	rs3760982	44286513	G	A	1.04 (0.99-1.09)	0.13	1.02 (0.92-1.13)	0.69	0.80	1.02 (0.92-1.13)	0.71	1.07 (0.99-1.15)	0.08	0.50
21q21.1	rs2823093	16520832	G	A	0.96 (0.91-1.01)	0.12	0.93 (0.83-1.04)	0.22	0.66	1.00 (0.88-1.14)	0.96	0.92 (0.84-1.00)	0.05	0.32
22q12.2	rs132390	29621477	A	G	0.94 (0.82-1.08)	0.38	1.14 (0.88-1.48)	0.33	0.23	1.13 (0.82-1.54)	0.45	1.19 (0.97-1.46)	0.09	0.78
22q13.1	rs6001930	40876234	A	G	1.06 (0.98-1.15)	0.12	1.10 (0.94-1.30)	0.24	0.70	0.95 (0.79-1.14)	0.58	1.05 (0.93-1.19)	0.40	0.37

[#] Reference and effect allele

Table S5 Associations of 74 previously reported breast cancer susceptibility variants with breast cancer by tumor HER2-status in *BRCA1* and *BRCA2* carriers. Associations with p<0.01 in bold.

					***BRCA1* carriers**					***BRCA2* carriers**				
					**HER2-negative**		**HER2-positive**			**HER2-negative**		**HER2-positive**		
**Locus**	**SNP**	**Position**	**Ref#**	**Eff#**	**HR (95%CI)**	**p**	**HR (95%CI)**	**p**	**Phet**	**HR (95%CI)**	**p**	**HR (95%CI)**	**p**	**Phet**
1p36.22	rs616488	10566215	A	G	0.96 (0.92-1.01)	0.13	0.95 (0.77-1.16)	0.61	0.90	1.00 (0.93-1.07)	0.95	0.86 (0.63-1.16)	0.32	0.38
1p13.2	rs12022378	114448389	G	A	1.03 (0.97-1.1)	0.32	1.13 (0.86-1.48)	0.37	0.54	1.08 (0.98-1.18)	0.11	0.87 (0.61-1.24)	0.45	0.29
1p11.2	rs11249433	121280613	A	G	1.00 (0.96-1.05)	0.95	0.87 (0.71-1.07)	0.19	0.22	1.05 (0.98-1.13)	0.16	1.09 (0.84-1.42)	0.51	0.80
1q32.1_1	rs6678914	202187176	G	A	0.95 (0.91-1.00)	0.05	1.20 (0.99-1.45)	0.06	0.03	1.04 (0.97-1.12)	0.23	1.15 (0.9-1.47)	0.26	0.47
1q32.1_2	rs4245739	204518842	A	C	1.10 (1.05-1.16)	**1.6x10^{-4}**	1.02 (0.83-1.27)	0.82	0.54	0.97 (0.9-1.04)	0.39	0.97 (0.72-1.30)	0.83	0.99
2p24.1	rs12710696	19320803	G	A	1.00 (0.96-1.05)	0.91	1.16 (0.94-1.42)	0.18	0.22	0.98 (0.92-1.05)	0.57	1.05 (0.83-1.33)	0.68	0.60
2q14.2	rs4849887	121245122	G	A	1.01 (0.93-1.08)	0.86	1.18 (0.87-1.62)	0.29	0.35	0.99 (0.88-1.1)	0.83	0.9 (0.58-1.39)	0.63	0.70
2q31.1	rs2016394	172972971	G	A	1.02 (0.97-1.07)	0.38	0.95 (0.77-1.17)	0.63	0.53	0.98 (0.92-1.05)	0.60	1.01 (0.78-1.31)	0.92	0.83
2q31.1	rs1550623	174212894	A	G	0.98 (0.91-1.04)	0.47	1.28 (0.97-1.67)	0.08	0.07	0.95 (0.87-1.04)	0.29	1.13 (0.86-1.5)	0.38	0.26
2q35	rs13387042	217905832	A	G	0.99 (0.95-1.04)	0.79	0.83 (0.68-1.01)	0.06	0.09	1.00 (0.94-1.07)	0.97	0.79 (0.63-1.01)	0.06	0.09
2q35	rs16857609	218296508	G	A	1.06 (1.01-1.12)	0.02	0.92 (0.72-1.16)	0.48	0.26	1.00 (0.92-1.08)	0.95	0.85 (0.62-1.16)	0.31	0.36
3p26.1	rs6762644	4742276	A	G	1.02 (0.97-1.07)	0.51	1.16 (0.93-1.44)	0.18	0.27	0.99 (0.92-1.06)	0.74	0.98 (0.74-1.3)	0.89	0.96
3p24.1	rs4973768	27416013	G	A	1.02 (0.97-1.06)	0.49	1.00 (0.84-1.20)	1.00	0.88	1.08 (1.01-1.15)	0.03	1.21 (0.93-1.56)	0.16	0.44
3p24.1	rs12493607	30682939	C	G	0.98 (0.94-1.03)	0.49	1.15 (0.95-1.4)	0.15	0.14	0.98 (0.91-1.06)	0.64	1.02 (0.78-1.34)	0.86	0.78
4q24	rs9790517	106084778	G	A	0.99 (0.94-1.04)	0.71	0.95 (0.75-1.2)	0.65	0.74	0.96 (0.88-1.04)	0.33	0.95 (0.7-1.29)	0.76	0.97
4q34.1	rs6828523	175846426	C	A	1.04 (0.97-1.12)	0.28	0.93 (0.67-1.29)	0.67	0.53	0.99 (0.89-1.1)	0.84	0.91 (0.59-1.41)	0.67	0.74
5p15.33	rs10069690	1279790	G	A	1.24 (1.18-1.3)	**3.4x10^{-17}**	0.9 (0.71-1.13)	0.36	0.01	1.12 (1.04-1.21)	**2.9x10^{-3}**	1.03 (0.79-1.36)	0.82	0.58
5p15.33	rs7725218	1282414	G	A	1.09 (1.04-1.14)	**3.5x10^{-4}**	1.01 (0.8-1.26)	0.96	0.51	1.05 (0.98-1.13)	0.18	1.04 (0.79-1.36)	0.80	0.93
5p15.33	rs2736108	1297488	G	A	0.88 (0.83-0.92)	**5.1x10^{-7}**	0.98 (0.79-1.22)	0.85	0.37	0.93 (0.86-1.00)	0.05	0.88 (0.66-1.16)	0.35	0.72
5p12	rs10941679	44706498	A	G	1.00 (0.94-1.05)	0.87	0.89 (0.70-1.14)	0.36	0.41	1.07 (0.99-1.15)	0.10	1.08 (0.82-1.44)	0.57	0.92
5q11.2	rs889312	56031884	A	C	1.02 (0.97-1.07)	0.43	0.94 (0.74-1.19)	0.60	0.52	1.00 (0.93-1.08)	0.91	1.35 (1.06-1.73)	0.01	0.03
5q11.3	rs10472076	58184061	A	G	1.00 (0.95-1.05)	0.94	1.03 (0.85-1.26)	0.73	0.74	0.97 (0.90-1.04)	0.35	1.05 (0.81-1.37)	0.71	0.58
5q11.3	rs1353747	58337481	A	C	0.97 (0.9-1.04)	0.39	1.02 (0.74-1.40)	0.92	0.77	0.99 (0.89-1.11)	0.93	0.68 (0.42-1.1)	0.12	0.15
5q33.3	rs1432679	158244083	A	G	1.05 (1.01-1.1)	0.02	0.92 (0.75-1.12)	0.40	0.20	0.97 (0.91-1.04)	0.44	1.09 (0.85-1.4)	0.50	0.43
6p25.3	rs11242675	1318878	A	G	0.97 (0.92-1.02)	0.21	0.88 (0.71-1.08)	0.22	0.38	0.97 (0.90-1.04)	0.35	1.17 (0.89-1.55)	0.26	0.22

6p24.3	rs9348512	10456706	C	A	0.99 (0.94-1.03)	0.57	1.05 (0.84-1.31)	0.68	0.62	0.84 (0.78-0.90)	**1.5x10^{-6}**	0.95 (0.75-1.2)	0.64	0.39
6p23	rs204247	13722523	A	G	1.02 (0.97-1.06)	0.49	0.86 (0.71-1.04)	0.11	0.10	1.11 (1.04-1.19)	**2.1x10^{-3}**	0.97 (0.75-1.26)	0.83	0.37
6q14	rs17530068	82193109	A	G	1.01 (0.96-1.07)	0.57	1.16 (0.92-1.46)	0.20	0.29	1.12 (1.04-1.21)	**3.4x10^{-3}**	0.92 (0.68-1.25)	0.58	0.24
6q25.1	rs3757318	151914113	G	A	1.20 (1.11-1.3)	**7.9x10^{-6}**	1.21 (0.87-1.68)	0.26	0.96	1.19 (1.06-1.34)	**3.7x10^{-3}**	0.91 (0.56-1.47)	0.70	0.31
6q25.1	rs2046210	151948366	G	A	1.17 (1.11-1.22)	**1.5x10^{-10}**	1.12 (0.91-1.38)	0.29	0.71	1.09 (1.02-1.17)	0.02	0.85 (0.63-1.15)	0.30	0.15
7q35	rs720475	144074929	G	A	0.96 (0.91-1.01)	0.12	1.18 (0.93-1.50)	0.17	0.11	0.91 (0.84-0.98)	0.02	1.45 (1.14-1.86)	**3.0x10^{-3}**	**9.3x10^{-4}**
8p12	rs9693444	29509616	C	A	1.00 (0.95-1.05)	0.98	1.04 (0.84-1.28)	0.72	0.73	0.96 (0.90-1.03)	0.31	1.19 (0.93-1.54)	0.17	0.14
8q21.11	rs6472903	76230301	A	C	1.03 (0.97-1.1)	0.27	0.82 (0.62-1.08)	0.16	0.13	0.95 (0.86-1.04)	0.28	1.13 (0.8-1.59)	0.50	0.38
8q21.11	rs2943559	76417937	A	G	1.08 (1-1.17)	0.05	0.86 (0.57-1.29)	0.46	0.29	1.06 (0.94-1.19)	0.33	1.28 (0.83-1.96)	0.26	0.45
8q24.21	rs11780156	129194641	G	A	0.95 (0.9-1.01)	0.09	0.97 (0.75-1.26)	0.83	0.88	0.93 (0.86-1.01)	0.10	1.21 (0.9-1.62)	0.21	0.13
8q24.21	rs13281615	128355618	A	G	1.01 (0.97-1.06)	0.51	1.05 (0.87-1.26)	0.62	0.76	1.04 (0.97-1.12)	0.23	1.08 (0.84-1.39)	0.55	0.80
9p21.3	rs1011970	22062134	C	A	1.04 (0.98-1.11)	0.17	0.82 (0.61-1.10)	0.18	0.13	1.00 (0.91-1.09)	0.94	1.28 (0.97-1.71)	0.09	0.12
9q31.2	rs10759243	110306115	C	A	0.98 (0.93-1.03)	0.34	1.05 (0.86-1.29)	0.63	0.51	1.02 (0.94-1.1)	0.67	0.89 (0.66-1.19)	0.42	0.41
9q31.2	rs865686	110888478	A	C	1.00 (0.95-1.04)	0.85	0.91 (0.72-1.15)	0.44	0.50	0.98 (0.91-1.05)	0.59	1.06 (0.8-1.41)	0.68	0.62
10p12.31	rs7072776	22032942	G	A	0.97 (0.92-1.02)	0.20	1.15 (0.93-1.41)	0.19	0.14	1.04 (0.97-1.12)	0.22	0.76 (0.59-0.98)	0.03	0.02
10p12.31	rs11814448	22315843	A	C										
10q21.2	rs10995190	64278682	G	A	0.99 (0.93-1.06)	0.80	0.99 (0.76-1.31)	0.97	0.99	0.99 (0.9-1.08)	0.78	0.66 (0.44-0.98)	0.04	0.07
10q22.3	rs704010	80841148	G	A	1.01 (0.97-1.06)	0.53	1.01 (0.81-1.25)	0.95	0.95	0.99 (0.92-1.06)	0.71	1.08 (0.86-1.37)	0.51	0.49
10q25.2	rs7904519	114773927	A	G	1.09 (1.04-1.14)	**2.2x10^{-4}**	1.21 (1.00-1.46)	0.05	0.33	1.05 (0.98-1.12)	0.15	0.91 (0.72-1.15)	0.43	0.28
10q26.12	rs2981579	123337335	G	A	0.99 (0.95-1.04)	0.77	1.05 (0.86-1.30)	0.62	0.60	1.25 (1.17-1.34)	**1.9x10^{-10}**	1.23 (0.94-1.59)	0.13	0.88
10q26.12	rs11199914	123093901	G	A	1.02 (0.97-1.07)	0.53	1.1 (0.88-1.38)	0.38	0.50	0.94 (0.87-1.01)	0.08	1.06 (0.8-1.41)	0.67	0.42
11p15.5	rs3817198	1909006	A	G	1.09 (1.04-1.14)	**2.7x10^{-4}**	0.97 (0.80-1.19)	0.79	0.30	1.16 (1.07-1.24)	**7.4x10^{-5}**	0.91 (0.69-1.19)	0.49	0.12
11q13.1	rs3903072	65583066	C	A	1.00 (0.96-1.05)	0.86	0.92 (0.76-1.11)	0.38	0.39	1.00 (0.93-1.07)	0.96	0.95 (0.75-1.2)	0.67	0.71
11q13.3	rs554219	69331642	C	G	1.06 (0.99-1.13)	0.10	0.87 (0.62-1.23)	0.43	0.30	1.11 (1.01-1.22)	0.04	0.96 (0.68-1.38)	0.84	0.48
11q13.3	c11_pos69088342	69379161	C	A	1.05 (0.95-1.16)	0.31	0.98 (0.64-1.52)	0.94	0.78	1.11 (0.97-1.27)	0.14	0.93 (0.56-1.54)	0.77	0.52
11q13.3	rs494406	69344241	G	A	1.02 (0.97-1.07)	0.51	1.10 (0.88-1.38)	0.38	0.50	1.08 (1.00-1.16)	0.04	0.87 (0.66-1.14)	0.31	0.15
11q24.3	rs11820646	129461171	G	A	0.93 (0.89-0.98)	**4.7x10^{-3}**	0.85 (0.69-1.05)	0.13	0.42	0.91 (0.85-0.98)	**7.7x10^{-3}**	0.95 (0.74-1.23)	0.71	0.75
12p13.1	rs12422552	14413931	G	C	1.02 (0.97-1.08)	0.38	0.90 (0.70-1.16)	0.42	0.36	0.98 (0.91-1.06)	0.65	1.04 (0.76-1.42)	0.81	0.75
12p11.22	rs10771399	28155080	A	G	0.85 (0.79-0.91)	**1.5x10^{-5}**	0.81 (0.57-1.15)	0.24	0.81	0.94 (0.84-1.04)	0.23	0.58 (0.36-0.93)	0.02	0.06

12q22	rs17356907	96027759	A	G	0.95 (0.9-0.99)	0.03	0.95 (0.75-1.21)	0.68	0.98	1.04 (0.97-1.12)	0.30	0.78 (0.59-1.03)	0.08	0.07
12q24.21	rs1292011	115836522	A	G	0.99 (0.94-1.04)	0.66	1.04 (0.85-1.28)	0.68	0.64	0.91 (0.85-0.97)	**6.7x10^{-3}**	0.98 (0.75-1.27)	0.88	0.61
13q13.1	rs11571833	32972626	T	A										
14q13.3	rs2236007	37132769	G	A	0.99 (0.93-1.04)	0.63	0.82 (0.64-1.06)	0.14	0.20	1.03 (0.95-1.12)	0.50	0.81 (0.57-1.13)	0.21	0.20
14q24.1	rs2588809	68660428	G	A	0.95 (0.89-1.01)	0.08	1.04 (0.81-1.34)	0.75	0.50	0.96 (0.88-1.06)	0.45	1.27 (0.93-1.75)	0.14	0.14
14q24.1	rs999737	69034682	G	A	0.95 (0.9-1.01)	0.08	1.00 (0.77-1.30)	0.98	0.71	0.98 (0.91-1.07)	0.71	0.83 (0.6-1.14)	0.25	0.33
14q32.11	rs941764	91841069	A	G	1.02 (0.97-1.07)	0.44	1.04 (0.82-1.31)	0.76	0.89	1.00 (0.94-1.08)	0.89	1.12 (0.89-1.42)	0.34	0.40
16q12.1_1	rs3803662	52586341	G	A	1.05 (1-1.1)	0.07	1.20 (0.98-1.49)	0.08	0.23	1.25 (1.17-1.35)	**9.5x10^{-10}**	1.08 (0.84-1.4)	0.53	0.32
16q12.1_2	rs11075995	53855291	A	T	0.99 (0.94-1.05)	0.84	1.20 (0.98-1.47)	0.08	0.10	0.98 (0.90-1.06)	0.62	1.27 (0.99-1.64)	0.06	0.07
16q12.1_2	rs17817449	53813367	A	C	0.96 (0.92-1.01)	0.10	0.82 (0.67-1.00)	0.06	0.15	0.91 (0.84-0.97)	**6.0x10^{-3}**	1.23 (0.95-1.60)	0.12	0.04
16q23.2	rs13329835	80650805	A	G	1.04 (0.99-1.1)	0.13	1.10 (0.87-1.4)	0.42	0.67	1.02 (0.95-1.10)	0.60	1.10 (0.85-1.43)	0.45	0.59
17q22	rs6504950	53056471	G	A	0.98 (0.93-1.04)	0.55	0.98 (0.78-1.24)	0.89	1.00	1.02 (0.95-1.11)	0.55	1.19 (0.91-1.55)	0.21	0.34
18q11.2	rs527616	24337424	C	G	1 (0.96-1.05)	0.91	0.87 (0.70-1.08)	0.21	0.24	1.00 (0.93-1.07)	0.97	0.77 (0.59-0.99)	0.05	0.07
18q11.2	rs1436904	22824665	A	C	0.98 (0.94-1.03)	0.44	1.04 (0.85-1.28)	0.69	0.60	0.97 (0.91-1.04)	0.46	0.89 (0.69-1.15)	0.37	0.52
19p13.11	rs8170	17389704	G	A	1.18 (1.11-1.25)	**3.7x10^{-8}**	1.33 (1.05-1.68)	0.02	0.34	0.99 (0.90-1.08)	0.75	0.81 (0.56-1.16)	0.25	0.33
19p13.11	rs4808801	18571141	A	G	1.00 (0.95-1.04)	0.85	0.82 (0.67-1.00)	0.05	0.08	0.96 (0.89-1.03)	0.27	1.07 (0.83-1.38)	0.61	0.46
19q13.31	rs3760982	44286513	G	A	1.02 (0.97-1.07)	0.44	1.16 (0.93-1.43)	0.19	0.29	1.05 (0.98-1.12)	0.16	1.07 (0.85-1.36)	0.55	0.85
21q21.1	rs2823093	16520832	G	A	0.97 (0.92-1.02)	0.21	0.79 (0.63-0.99)	0.04	0.10	0.95 (0.88-1.03)	0.24	0.82 (0.6-1.13)	0.23	0.41
22q12.2	rs132390	29621477	A	G	1.00 (0.88-1.13)	0.97	0.84 (0.47-1.50)	0.55	0.58	1.08 (0.88-1.32)	0.45	1.71 (0.96-3.06)	0.07	0.19
22q13.1	rs6001930	40876234	A	G	1.08 (1.01-1.16)	0.03	0.95 (0.66-1.37)	0.80	0.52	0.99 (0.88-1.12)	0.91	1.10 (0.72-1.67)	0.65	0.67

[#] Reference and effect allele

Table S6 Associations of 74 previously reported breast cancer susceptibility variants with breast cancer by tumor triple negative status in *BRCA1* and *BRCA2* mutation carriers. Associations with p<0.01 in bold.

					***BRCA1* carriers**					***BRCA2* carriers**				
					**triple negative**		**not triple negative**			**triple negative**		**not triple negative**		
**Locus**	**SNP**	**Position**	**Ref#**	**Eff#**	**HR (95%CI)**	**p**	**HR (95%CI)**	**p**	$\mathbf{p_{het}}$	**HR (95%CI)**	**p**	**HR (95%CI)**	**p**	$\mathbf{p_{het}}$
1p36.22	rs616488	10566215	A	G	0.97 (0.91-1.04)	0.38	0.94 (0.84-1.05)	0.26	0.64	1.02 (0.79-1.31)	0.90	0.97 (0.9-1.05)	0.49	0.77
1p13.2	rs12022378	114448389	G	A	1.01 (0.93-1.09)	0.89	1.1 (0.96-1.27)	0.18	0.35	1.47 (1.1-1.97)	**8.6×10^{-3}**	0.98 (0.89-1.08)	0.72	0.02
1p11.2	rs11249433	121280613	A	G	1.00 (0.94-1.06)	0.9	0.99 (0.89-1.1)	0.82	0.91	1.08 (0.87-1.35)	0.47	1.05 (0.98-1.13)	0.16	0.80
1q32.1_1	rs6678914	202187176	G	A	0.97 (0.91-1.03)	0.29	1.01 (0.92-1.12)	0.77	0.50	0.94 (0.75-1.18)	0.59	1.08 (1.00-1.16)	0.04	0.30
1q32.1_2	rs4245739	204518842	A	C	1.12 (1.06-1.20)	**2.2×10^{-4}**	1.04 (0.93-1.16)	0.49	0.29	1.35 (1.06-1.71)	0.01	0.91 (0.84-0.98)	0.02	**4.9×10^{-3}**
2p24.1	rs12710696	19320803	G	A	1.00 (0.95-1.07)	0.87	1.04 (0.93-1.16)	0.46	0.63	1.1 (0.9-1.35)	0.35	0.97 (0.90-1.04)	0.39	0.28
2q14.2	rs4849887	121245122	G	A	1.03 (0.94-1.14)	0.50	1 (0.84-1.19)	1.00	0.78	0.81 (0.52-1.26)	0.35	1.01 (0.9-1.13)	0.92	0.39
2q31.1	rs2016394	172972971	G	A	1.03 (0.97-1.09)	0.37	0.98 (0.88-1.09)	0.70	0.51	1.05 (0.83-1.34)	0.68	0.97 (0.91-1.05)	0.48	0.59
2q31.1	rs1550623	174212894	A	G	1.03 (0.95-1.12)	0.42	0.95 (0.81-1.11)	0.50	0.40	0.81 (0.6-1.08)	0.16	1.00 (0.91-1.10)	0.94	0.19
2q35	rs13387042	217905832	A	G	1.04 (0.99-1.11)	0.13	0.84 (0.76-0.93)	**1.0×10^{-3}**	**1.6×10^{-3}**	0.99 (0.81-1.21)	0.92	0.97 (0.90-1.03)	0.34	0.85
2q35	rs16857609	218296508	G	A	1.03 (0.97-1.11)	0.33	1.07 (0.95-1.21)	0.27	0.68	1.03 (0.79-1.35)	0.80	0.97 (0.90-1.05)	0.43	0.67
3p26.1	rs6762644	4742276	A	G	0.98 (0.92-1.05)	0.61	1.16 (1.04-1.29)	**7.8×10^{-3}**	0.03	0.93 (0.7-1.24)	0.62	1.00 (0.92-1.08)	0.95	0.68
3p24.1	rs4973768	27416013	G	A	1.00 (0.94-1.06)	0.94	1.05 (0.95-1.15)	0.36	0.46	1.07 (0.86-1.34)	0.54	1.10 (1.02-1.18)	**0.01**	0.87
3p24.1	rs12493607	30682939	C	G	0.99 (0.93-1.05)	0.73	1.01 (0.9-1.12)	0.92	0.82	1.2 (0.95-1.52)	0.12	0.95 (0.88-1.03)	0.21	0.09
4q24	rs9790517	106084778	G	A	0.96 (0.89-1.03)	0.22	1.05 (0.93-1.19)	0.41	0.25	0.91 (0.69-1.2)	0.49	0.97 (0.89-1.05)	0.46	0.68
4q34.1	rs6828523	175846426	C	A	1.08 (0.98-1.18)	0.12	0.92 (0.77-1.11)	0.38	0.20	1.02 (0.7-1.48)	0.93	0.97 (0.86-1.09)	0.64	0.84
5p15.33	rs10069690	1279790	G	A	1.27 (1.20-1.36)	**5.2×10^{-14}**	1.06 (0.94-1.19)	0.34	0.02	1.58 (1.26-1.99)	**9.8×10^{-5}**	1.04 (0.96-1.13)	0.37	**2.0×10^{-3}**
5p15.33	rs7725218	1282414	G	A	1.10 (1.03-1.17)	**2.3×10^{-3}**	1.04 (0.93-1.16)	0.50	0.45	1.33 (1.03-1.7)	0.03	1.00 (0.93-1.08)	0.90	0.06
5p15.33	rs2736108	1297488	G	A	0.85 (0.80-0.91)	**5.4×10^{-6}**	0.97 (0.87-1.1)	0.66	0.10	1.08 (0.84-1.39)	0.55	0.89 (0.82-0.97)	**5.2×10^{-3}**	0.20
5p12	rs10941679	44706498	A	G	0.98 (0.92-1.05)	0.63	1 (0.88-1.13)	0.99	0.85	0.98 (0.75-1.28)	0.89	1.09 (1.00-1.18)	0.04	0.51
5q11.2	rs889312	56031884	A	C	0.99 (0.92-1.06)	0.72	1.08 (0.96-1.22)	0.21	0.28	0.84 (0.64-1.1)	0.21	1.08 (1.00-1.17)	0.04	0.10
5q11.3	rs10472076	58184061	A	G	1.00 (0.95-1.06)	0.92	1.01 (0.91-1.12)	0.83	0.90	1.05 (0.82-1.35)	0.68	0.96 (0.89-1.04)	0.33	0.54
5q11.3	rs1353747	58337481	A	C	0.92 (0.83-1.02)	0.12	1.1 (0.93-1.29)	0.28	0.13	1.15 (0.79-1.66)	0.48	0.92 (0.81-1.04)	0.20	0.33
5q33.3	rs1432679	158244083	A	G	1.05 (0.99-1.12)	0.09	1.03 (0.93-1.15)	0.53	0.79	0.82 (0.67-1.01)	0.07	1.02 (0.95-1.09)	0.57	0.07
6p25.3	rs11242675	1318878	A	G	0.93 (0.87-0.99)	0.02	1.04 (0.93-1.16)	0.50	0.13	1.04 (0.81-1.34)	0.73	0.98 (0.91-1.06)	0.62	0.67

6p24.3	rs9348512	10456706	C	A	0.99 (0.92-1.05)	0.65	1.01 (0.9-1.13)	0.84	0.73	0.79 (0.62-1)	0.05	0.87 (0.81-0.93)	**$1.0x10^{-4}$**	0.48
6p23	rs204247	13722523	A	G	0.99 (0.94-1.05)	0.78	1.02 (0.92-1.13)	0.70	0.67	1.06 (0.82-1.36)	0.67	1.1 (1.02-1.18)	**$7.8x10^{-3}$**	0.79
6q14	rs17530068	82193109	A	G	1.02 (0.96-1.09)	0.48	1.04 (0.92-1.17)	0.56	0.89	1.19 (0.9-1.57)	0.23	1.08 (0.99-1.17)	0.07	0.56
6q25.1	rs3757318	151914113	G	A	1.23 (1.11-1.36)	**$6.7x10^{-5}$**	1.14 (0.94-1.37)	0.18	0.53	1.51 (1.07-2.14)	0.02	1.09 (0.96-1.25)	0.17	0.12
6q25.1	rs2046210	151948366	G	A	1.23 (1.16-1.31)	**$5.5x10^{-12}$**	1.01 (0.91-1.13)	0.79	**$6.8x10^{-3}$**	1.32 (1.03-1.7)	0.03	1.02 (0.94-1.10)	0.69	0.08
7q35	rs720475	144074929	G	A	0.97 (0.90-1.04)	0.35	1.01 (0.88-1.15)	0.92	0.64	0.87 (0.66-1.14)	0.30	0.99 (0.91-1.07)	0.74	0.40
8p12	rs9693444	29509616	C	A	1.04 (0.98-1.11)	0.20	0.93 (0.83-1.03)	0.18	0.12	0.87 (0.68-1.12)	0.29	1.01 (0.94-1.09)	0.73	0.31
8q21.11	rs6472903	76230301	A	C	1.06 (0.98-1.15)	0.12	0.88 (0.76-1.02)	0.10	0.06	1.01 (0.72-1.41)	0.95	0.96 (0.87-1.06)	0.45	0.80
8q21.11	rs2943559	76417937	A	G	1.07 (0.97-1.19)	0.18	1.02 (0.84-1.24)	0.85	0.69	1.23 (0.82-1.82)	0.31	1.06 (0.94-1.20)	0.32	0.54
8q24.21	rs11780156	129194641	G	A	0.93 (0.86-.001)	0.06	0.99 (0.87-1.12)	0.86	0.49	1.07 (0.8-1.42)	0.66	0.95 (0.87-1.04)	0.24	0.49
8q24.21	rs13281615	128355618	A	G	0.99 (0.93-1.05)	0.70	1.08 (0.97-1.2)	0.14	0.20	1.08 (0.86-1.35)	0.53	1.04 (0.97-1.12)	0.26	0.80
9p21.3	rs1011970	22062134	C	A	1.09 (1.01-1.18)	0.02	0.87 (0.74-1.01)	0.06	0.02	0.95 (0.71-1.28)	0.76	1.04 (0.95-1.15)	0.35	0.59
9q31.2	rs10759243	110306115	C	A	0.98 (0.92-1.05)	0.58	0.99 (0.88-1.1)	0.82	0.95	0.88 (0.67-1.16)	0.35	1.02 (0.95-1.10)	0.58	0.34
9q31.2	rs865686	110888478	A	C	1.00 (0.94-1.07)	0.99	0.98 (0.87-1.1)	0.72	0.79	1.21 (0.94-1.55)	0.14	0.95 (0.89-1.03)	0.23	0.11
10p12.31	rs7072776	22032942	G	A	0.93 (0.87-0.99)	0.03	1.13 (1.01-1.26)	0.03	**$7.9x10^{-3}$**	0.82 (0.65-1.03)	0.09	1.04 (0.97-1.12)	0.27	0.07
10p12.31	rs11814448	22315843	A	C										
10q21.2	rs10995190	64278682	G	A	1.04 (0.96-1.12)	0.34	0.87 (0.75-1.02)	0.09	0.09	0.86 (0.61-1.21)	0.38	0.96 (0.87-1.06)	0.40	0.57
10q22.3	rs704010	80841148	G	A	0.98 (0.92-1.04)	0.47	1.11 (1-1.24)	0.06	0.09	0.92 (0.75-1.12)	0.42	1.01 (0.95-1.08)	0.71	0.41
10q25.2	rs7904519	114773927	A	G	1.08 (1.02-1.15)	**$6.9x10^{-3}$**	1.13 (1.03-1.25)	0.01	0.49	1.05 (0.83-1.34)	0.66	1.03 (0.96-1.10)	0.46	0.85
10q26.12	rs2981579	123337335	G	A	0.92 (0.87-0.98)	**$8.9x10^{-3}$**	1.18 (1.06-1.32)	**$2.1x10^{-3}$**	**$6.4x10^{-4}$**	0.86 (0.7-1.05)	0.15	1.33 (1.24-1.43)	**$3.8x10^{-16}$**	**$1.7x10^{-4}$**
10q26.12	rs11199914	123093901	G	A	1.02 (0.96-1.09)	0.45	1.02 (0.91-1.15)	0.70	0.98	0.93 (0.71-1.21)	0.58	0.96 (0.89-1.03)	0.24	0.85
11p15.5	rs3817198	1909006	A	G	1.07 (1.01-1.14)	0.03	1.09 (0.98-1.22)	0.11	0.80	1.09 (0.85-1.4)	0.50	1.13 (1.05-1.21)	**$1.7x10^{-3}$**	0.82
11q13.1	rs3903072	65583066	C	A	1.01 (0.95-1.07)	0.77	0.96 (0.86-1.06)	0.39	0.44	0.93 (0.75-1.14)	0.46	1.00 (0.94-1.07)	0.90	0.49
11q13.3	rs554219	69331642	C	G	1.01 (0.92-1.10)	0.87	1.09 (0.93-1.27)	0.30	0.49	0.82 (0.57-1.18)	0.29	1.14 (1.03-1.25)	**$8.4x10^{-3}$**	0.11
11q13.3	c11_pos69088342	69379161	C	A	1.02 (0.89-1.17)	0.76	1.07 (0.84-1.35)	0.58	0.78	0.76 (0.45-1.29)	0.31	1.14 (1.00-1.31)	0.06	0.17
11q13.3	rs494406	69344241	G	A	1.02 (0.95-1.09)	0.63	1.03 (0.91-1.15)	0.67	0.91	1.01 (0.8-1.28)	0.93	1.06 (0.98-1.14)	0.14	0.73
11q24.3	rs11820646	129461171	G	A	0.93 (0.88-0.99)	0.03	0.92 (0.82-1.02)	0.12	0.78	0.78 (0.61-0.99)	0.04	0.94 (0.88-1.01)	0.10	0.17
12p13.1	rs12422552	14413931	G	C	1.02 (0.95-1.09)	0.55	0.99 (0.88-1.13)	0.93	0.76	1.16 (0.87-1.55)	0.30	0.96 (0.88-1.04)	0.33	0.25
12p11.22	rs10771399	28155080	A	G	0.86 (0.78-0.95)	**$3.3x10^{-3}$**	0.82 (0.68-0.99)	0.03	0.69	0.75 (0.51-1.1)	0.14	0.92 (0.82-1.02)	0.13	0.36

12q22	rs17356907	96027759	A	G	0.94 (0.88-1.00)	0.05	0.99 (0.88-1.11)	0.83	0.51	0.91 (0.72-1.14)	0.41	1.02 (0.95-1.10)	0.58	0.38
12q24.21	rs1292011	115836522	A	G	1.06 (1.00-1.13)	0.05	0.88 (0.79-0.98)	0.02	**9.1x10^{-3}**	1.03 (0.82-1.3)	0.80	0.90 (0.84-0.97)	**3.4x10^{-3}**	0.31
13q13.1	rs11571833	32972626	T	A										
14q13.3	rs2236007	37132769	G	A	0.96 (0.89-1.03)	0.30	0.98 (0.86-1.11)	0.73	0.85	1.31 (0.98-1.75)	0.07	0.95 (0.87-1.04)	0.27	0.06
14q24.1	rs2588809	68660428	G	A	0.94 (0.87-1.02)	0.13	0.99 (0.87-1.14)	0.93	0.55	1.01 (0.74-1.39)	0.93	1.00 (0.91-1.10)	1.00	0.94
14q24.1	rs999737	69034682	G	A	1.01 (0.94-1.09)	0.77	0.84 (0.74-0.97)	0.02	0.05	1.03 (0.8-1.32)	0.84	0.95 (0.88-1.03)	0.26	0.61
14q32.11	rs941764	91841069	A	G	1.00 (0.93-1.07)	0.96	1.09 (0.96-1.22)	0.18	0.31	0.96 (0.78-1.18)	0.72	1.03 (0.96-1.11)	0.42	0.57
16q12.1_1	rs3803662	52586341	G	A	0.98 (0.92-1.05)	0.59	1.25 (1.12-1.39)	**1.1x10^{-4}**	**1.7x10^{-3}**	1.17 (0.93-1.48)	0.18	1.24 (1.15-1.34)	**1.0x10^{-8}**	0.67
16q12.1_2	rs11075995	53855291	A	T	0.98 (0.92-1.05)	0.66	1.07 (0.95-1.21)	0.24	0.28	1.18 (0.91-1.52)	0.22	0.99 (0.91-1.07)	0.76	0.24
16q12.1_2	rs17817449	53813367	A	C	0.95 (0.89-1.00)	0.07	0.96 (0.86-1.06)	0.41	0.90	1.02 (0.79-1.31)	0.90	0.93 (0.86-1.00)	0.05	0.55
16q23.2	rs13329835	80650805	A	G	1.03 (0.96-1.10)	0.47	1.09 (0.96-1.24)	0.17	0.47	1.16 (0.92-1.46)	0.21	1.01 (0.93-1.09)	0.82	0.30
17q22	rs6504950	53056471	G	A	1.00 (0.94-1.07)	0.94	0.95 (0.85-1.07)	0.43	0.53	1.05 (0.83-1.32)	0.70	1.04 (0.97-1.12)	0.28	0.98
18q11.2	rs527616	24337424	C	G	0.98 (0.92-1.04)	0.47	1.01 (0.91-1.13)	0.82	0.63	0.97 (0.77-1.22)	0.81	0.97 (0.90-1.04)	0.35	0.96
18q11.2	rs1436904	22824665	A	C	0.98 (0.93-1.04)	0.57	1 (0.9-1.11)	0.95	0.78	0.96 (0.77-1.2)	0.73	0.96 (0.90-1.03)	0.31	0.98
19p13.11	rs8170	17389704	G	A	1.15 (1.07-1.24)	**1.6x10^{-4}**	1.26 (1.12-1.43)	**1.6x10^{-4}**	0.26	0.89 (0.65-1.22)	0.47	0.97 (0.89-1.07)	0.58	0.62
19p13.11	rs4808801	18571141	A	G	0.99 (0.93-1.05)	0.66	0.97 (0.87-1.07)	0.53	0.77	1.01 (0.8-1.28)	0.94	0.97 (0.90-1.04)	0.38	0.75
19q13.31	rs3760982	44286513	G	A	1.04 (0.98-1.11)	0.18	1.02 (0.91-1.14)	0.74	0.75	0.87 (0.71-1.08)	0.21	1.09 (1.02-1.16)	0.02	0.07
21q21.1	rs2823093	16520832	G	A	0.98 (0.91-1.04)	0.45	0.9 (0.8-1.01)	0.08	0.31	0.99 (0.76-1.3)	0.96	0.93 (0.85-1.01)	0.07	0.66
22q12.2	rs132390	29621477	A	G	0.97 (0.82-1.14)	0.72	1.02 (0.77-1.34)	0.91	0.80	0.68 (0.31-1.48)	0.33	1.24 (1.05-1.48)	0.01	0.16
22q13.1	rs6001930	40876234	A	G	1.05 (0.95-1.16)	0.32	1.11 (0.94-1.32)	0.23	0.63	0.77 (0.51-1.14)	0.19	1.05 (0.94-1.17)	0.37	0.16

[#] Reference and effect allele

Table S7 Associations of 74 previously reported breast cancer susceptibility variants with ductal breast in *BRCA1* and *BRCA2* mutation carriers. Associations with p<0.01 in bold.

					***BRCA1* carriers**			***BRCA2* carriers**		
**Locus**	**SNP**	**Position**	**Ref#**	**Eff#**	**HR (95%CI)**	**p**	**p_{het}**	**HR (95%CI)**	**p**	**p_{het}**
1p36.22	rs616488	10566215	A	G	0.94 (0.89-0.99)	0.02	0.08	0.98 (0.91-1.05)	0.57	0.98
1p13.2	rs12022378	114448389	G	A	1.00 (0.94-1.07)	0.94	0.05	1.09 (0.99-1.19)	0.07	0.09
1p11.2	rs11249433	121280613	A	G	1.03 (0.98-1.08)	0.20	**$2.1x10^{-3}$**	1.06 (0.99-1.13)	0.08	0.77
1q32.1_1	rs6678914	202187176	G	A	0.99 (0.94-1.04)	0.60	0.70	1.03 (0.97-1.10)	0.31	0.21
1q32.1_2	rs4245739	204518842	A	C	1.09 (1.04-1.15)	**$8.1x10^{-4}$**	0.73	1.00 (0.93-1.08)	0.94	0.04
2p24.1	rs12710696	19320803	G	A	1.04 (0.99-1.09)	0.10	0.05	1.04 (0.97-1.11)	0.27	**$2.4x10^{-3}$**
2q14.2	rs4849887	121245122	G	A	1.03 (0.95-1.11)	0.47	0.80	0.92 (0.82-1.03)	0.14	0.03
2q31.1	rs2016394	172972971	G	A	1.02 (0.97-1.07)	0.50	0.76	1.01 (0.94-1.08)	0.83	0.18
2q31.1	rs1550623	174212894	A	G	0.99 (0.93-1.06)	0.78	0.30	0.99 (0.90-1.09)	0.83	0.42
2q35	rs13387042	217905832	A	G	0.97 (0.92-1.01)	0.17	0.28	0.97 (0.91-1.03)	0.31	0.79
2q35	rs16857609	218296508	G	A	1.05 (1.00-1.11)	0.05	0.58	0.97 (0.9-1.05)	0.45	0.69
3p26.1	rs6762644	4742276	A	G	1.01 (0.96-1.06)	0.77	0.03	0.98 (0.91-1.05)	0.55	0.61
3p24.1	rs4973768	27416013	G	A	1.03 (0.98-1.07)	0.28	0.25	1.12 (1.05-1.20)	**$9.4x10^{-4}$**	0.13
3p24.1	rs12493607	30682939	C	G	1.00 (0.95-1.05)	0.85	0.94	0.98 (0.91-1.05)	0.58	0.62
4q24	rs9790517	106084778	G	A	0.99 (0.94-1.05)	0.74	0.71	0.97 (0.90-1.05)	0.48	0.50
4q34.1	rs6828523	175846426	C	A	1.00 (0.92-1.08)	0.97	0.12	0.93 (0.84-1.04)	0.22	0.06
5p15.33	rs10069690	1279790	G	A	1.20 (1.14-1.26)	**$2.0x10^{-11}$**	0.67	1.12 (1.04-1.21)	**$2.4x10^{-3}$**	0.56
5p15.33	rs7725218	1282414	G	A	1.09 (1.04-1.15)	**$4.6x10^{-4}$**	0.49	1.05 (0.98-1.13)	0.15	0.84
5p15.33	rs2736108	1297488	G	A	0.90 (0.86-0.95)	**$1.4x10^{-4}$**	0.26	0.89 (0.83-0.96)	**$2.7x10^{-3}$**	0.10
5p12	rs10941679	44706498	A	G	0.98 (0.93-1.04)	0.56	0.76	1.07 (0.99-1.16)	0.07	0.91
5q11.2	rs889312	56031884	A	C	1.03 (0.98-1.09)	0.22	0.18	1.07 (0.99-1.15)	0.08	0.21
5q11.3	rs10472076	58184061	A	G	0.99 (0.94-1.04)	0.73	0.23	1.00 (0.93-1.07)	0.91	0.24
5q11.3	rs1353747	58337481	A	C	1.00 (0.92-1.08)	0.97	0.22	0.99 (0.89-1.11)	0.93	0.11
5q33.3	rs1432679	158244083	A	G	1.07 (1.02-1.12)	**$3.8x10^{-3}$**	0.04	1.01 (0.94-1.08)	0.81	0.19
6p25.3	rs11242675	1318878	A	G	0.91 (0.86-0.96)	**$3.4x10^{-4}$**	**$3.5x10^{-5}$**	0.98 (0.92-1.05)	0.65	0.71
6p24.3	rs9348512	10456706	C	A	1.00 (0.95-1.05)	0.85	0.88	0.86 (0.80-0.92)	**$2.0x10^{-5}$**	0.68

6p23	rs204247	13722523	A	G	0.99 (0.94-1.04)	0.62	0.29	1.11 (1.04-1.19)	**$1.6x10^{-3}$**	0.35
6q14	rs17530068	82193109	A	G	1.01 (0.95-1.06)	0.78	0.15	1.10 (1.02-1.18)	0.02	0.98
6q25.1	rs3757318	151914113	G	A	1.17 (1.08-1.28)	**$2.3x10^{-4}$**	0.29	1.09 (0.96-1.23)	0.17	0.04
6q25.1	rs2046210	151948366	G	A	1.17 (1.11-1.22)	**$9.4x10^{-10}$**	0.91	1.05 (0.98-1.13)	0.14	0.79
7q35	rs720475	144074929	G	A	0.99 (0.93-1.04)	0.66	0.49	0.98 (0.91-1.05)	0.57	0.56
8p12	rs9693444	29509616	C	A	1.01 (0.96-1.06)	0.66	0.63	1.01 (0.94-1.08)	0.74	0.20
8q21.11	rs6472903	76230301	A	C	0.99 (0.93-1.05)	0.73	0.23	0.93 (0.85-1.02)	0.15	0.10
8q21.11	rs2943559	76417937	A	G	1.05 (0.96-1.14)	0.29	0.66	1.04 (0.93-1.17)	0.49	0.13
8q24.21	rs11780156	129194641	G	A	0.96 (0.90-1.01)	0.14	0.61	1.01 (0.93-1.09)	0.87	0.03
8q24.21	rs13281615	128355618	A	G	1.03 (0.98-1.08)	0.23	0.23	1.05 (0.98-1.12)	0.16	0.95
9p21.3	rs1011970	22062134	C	A	1.00 (0.93-1.07)	0.96	0.17	1.03 (0.94-1.12)	0.53	0.92
9q31.2	rs10759243	110306115	C	A	1.01 (0.96-1.06)	0.75	0.05	1.00 (0.93-1.07)	0.98	0.97
9q31.2	rs865686	110888478	A	C	0.98 (0.93-1.03)	0.46	0.36	1.00 (0.94-1.08)	0.89	0.39
10p12.31	rs7072776	22032942	G	A	1.00 (0.95-1.05)	0.97	0.32	0.98 (0.91-1.06)	0.63	0.16
10p12.31	rs11814448	22315843	A	C						
10q21.2	rs10995190	64278682	G	A	0.98 (0.92-1.05)	0.53	0.57	0.95 (0.87-1.04)	0.28	0.73
10q22.3	rs704010	80841148	G	A	1.02 (0.97-1.07)	0.45	0.84	1.01 (0.94-1.07)	0.87	0.65
10q25.2	rs7904519	114773927	A	G	1.09 (1.04-1.14)	**$4.0x10^{-4}$**	0.44	1.04 (0.97-1.11)	0.24	0.60
10q26.12	rs2981579	123337335	G	A	1.00 (0.95-1.04)	0.89	0.93	1.26 (1.18-1.35)	**$5.1x10^{-12}$**	0.40
10q26.12	rs11199914	123093901	G	A	1.02 (0.97-1.07)	0.49	0.66	0.95 (0.88-1.02)	0.14	0.87
11p15.5	rs3817198	1909006	A	G	1.07 (1.02-1.13)	**$6.2x10^{-3}$**	0.71	1.14 (1.06-1.22)	**$4.1x10^{-4}$**	0.49
11q13.1	rs3903072	65583066	C	A	0.98 (0.93-1.03)	0.36	0.22	1.01 (0.94-1.08)	0.79	0.29
11q13.3	rs554219	69331642	C	G	1.03 (0.96-1.11)	0.43	0.90	1.10 (1.00-1.21)	0.06	0.81
11q13.3	c11_pos69088342	69379161	C	A	1.02 (0.92-1.13)	0.71	0.57	1.09 (0.95-1.25)	0.24	0.97
11q13.3	rs494406	69344241	G	A	1.05 (0.99-1.10)	0.11	0.06	1.06 (0.98-1.14)	0.15	0.84
11q24.3	rs11820646	129461171	G	A	0.92 (0.88-0.97)	**$1.2x10^{-3}$**	0.52	0.91 (0.85-0.97)	**$4.6x10^{-3}$**	0.62
12p13.1	rs12422552	14413931	G	C	1.01 (0.96-1.07)	0.66	0.97	0.95 (0.89-1.03)	0.22	0.05
12p11.22	rs10771399	28155080	A	G	0.83 (0.76-0.90)	**$2.7x10^{-6}$**	0.19	0.86 (0.77-0.95)	**$5.2x10^{-3}$**	0.13
12q22	rs17356907	96027759	A	G	0.96 (0.91-1.01)	0.14	0.40	0.98 (0.92-1.06)	0.66	0.25

12q24.21	rs1292011	115836522	A	G	1.00 (0.95-1.05)	0.96	0.94	0.93 (0.87-1.00)	0.04	0.35
13q13.1	rs11571833	32972626	T	A						
14q13.3	rs2236007	37132769	G	A	0.97 (0.92-1.03)	0.36	0.64	0.98 (0.90-1.06)	0.57	0.24
14q24.1	rs2588809	68660428	G	A	0.95 (0.89-1.01)	0.10	0.55	1.03 (0.95-1.12)	0.52	0.24
14q24.1	rs999737	69034682	G	A	0.97 (0.91-1.02)	0.26	0.57	0.97 (0.89-1.05)	0.40	0.87
14q32.11	rs941764	91841069	A	G	1.04 (0.99-1.10)	0.10	0.17	1.04 (0.97-1.12)	0.24	0.18
16q12.1_1	rs3803662	52586341	G	A	1.06 (1.01-1.12)	0.02	0.79	1.21 (1.13-1.29)	**$1.3x10^{-7}$**	0.24
16q12.1_2	rs11075995	53855291	A	T	1.02 (0.96-1.07)	0.58	0.73	1.02 (0.94-1.10)	0.70	0.95
16q12.1_2	rs17817449	53813367	A	C	0.96 (0.92-1.01)	0.14	0.17	0.95 (0.89-1.01)	0.13	0.68
16q23.2	rs13329835	80650805	A	G	1.04 (0.98-1.10)	0.19	0.53	1.05 (0.97-1.13)	0.22	0.32
17q22	rs6504950	53056471	G	A	0.96 (0.91-1.02)	0.17	0.06	1.04 (0.97-1.12)	0.25	0.98
18q11.2	rs527616	24337424	C	G	0.99 (0.94-1.04)	0.60	0.92	0.97 (0.91-1.04)	0.45	0.64
18q11.2	rs1436904	22824665	A	C	0.99 (0.94-1.04)	0.61	0.88	0.97 (0.91-1.04)	0.42	0.56
19p13.11	rs8170	17389704	G	A	1.17 (1.10-1.24)	**$5.4x10^{-7}$**	0.29	0.95 (0.87-1.03)	0.20	0.40
19p13.11	rs4808801	18571141	A	G	0.97 (0.92-1.02)	0.24	0.39	0.95 (0.89-1.02)	0.20	0.23
19q13.31	rs3760982	44286513	G	A	1.01 (0.97-1.06)	0.57	0.10	1.03 (0.97-1.10)	0.31	0.25
21q21.1	rs2823093	16520832	G	A	0.95 (0.9-1.00)	0.07	0.93	0.91 (0.85-0.98)	0.02	0.15
22q12.2	rs132390	29621477	A	G	1.06 (0.93-1.21)	0.38	0.02	1.14 (0.96-1.36)	0.13	0.75
22q13.1	rs6001930	40876234	A	G	1.10 (1.03-1.19)	**$8.1x10^{-3}$**	0.08	1.01 (0.90-1.12)	0.90	1.00

Reference and effect allele

Table S8 Associations of 74 previously reported breast cancer susceptibility variants with lobular breast cancer in *BRCA2* mutation carriers. Associations with p<0.01 in bold.

**Locus**	**SNP**	**Position**	**Ref**[#]	**Eff**[#]	**HR (95%CI)**	**p**	p_{het}
1p36.22	rs616488	10566215	A	G	0.95 (0.76-1.18)	0.62	0.75
1p13.2	rs12022378	114448389	G	A	0.95 (0.73-1.25)	0.74	0.48
1p11.2	rs11249433	121280613	A	G	1.06 (0.86-1.30)	0.58	0.97
1q32.1_1	rs6678914	202187176	G	A	1.20 (0.99-1.45)	0.07	0.19
1q32.1_2	rs4245739	204518842	A	C	0.86 (0.68-1.09)	0.22	0.32
2p24.1	rs12710696	19320803	G	A	0.76 (0.61-0.94)	0.01	0.01
2q14.2	rs4849887	121245122	G	A	1.02 (0.73-1.43)	0.89	0.77
2q31.1	rs2016394	172972971	G	A	0.81 (0.66-0.99)	0.04	0.05
2q31.1	rs1550623	174212894	A	G	0.93 (0.71-1.21)	0.58	0.70
2q35	rs13387042	217905832	A	G	0.85 (0.70-1.03)	0.09	0.14
2q35	rs16857609	218296508	G	A	1.14 (0.92-1.42)	0.24	0.15
3p26.1	rs6762644	4742276	A	G	1.02 (0.83-1.25)	0.84	0.73
3p24.1	rs4973768	27416013	G	A	1.09 (0.88-1.36)	0.41	0.99
3p24.1	rs12493607	30682939	C	G	0.99 (0.80-1.22)	0.90	0.99
4q24	rs9790517	106084778	G	A	1.08 (0.85-1.37)	0.54	0.32
4q34.1	rs6828523	175846426	C	A	0.82 (0.57-1.17)	0.27	0.30
5p15.33	rs10069690	1279790	G	A	0.97 (0.76-1.23)	0.77	0.23
5p15.33	rs7725218	1282414	G	A	0.96 (0.77-1.20)	0.75	0.44
5p15.33	rs2736108	1297488	G	A	1.01 (0.80-1.27)	0.96	0.43
5p12	rs10941679	44706498	A	G	1.03 (0.82-1.29)	0.79	0.74
5q11.2	rs889312	56031884	A	C	1.15 (0.92-1.43)	0.21	0.37
5q11.3	rs10472076	58184061	A	G	0.87 (0.69-1.09)	0.22	0.28
5q11.3	rs1353747	58337481	A	C	0.79 (0.54-1.15)	0.21	0.29
5q33.3	rs1432679	158244083	A	G	0.94 (0.76-1.17)	0.60	0.66
6p25.3	rs11242675	1318878	A	G	0.92 (0.75-1.14)	0.46	0.50
6p24.3	rs9348512	10456706	C	A	0.77 (0.63-0.95)	0.01	0.32
6p23	rs204247	13722523	A	G	1.06 (0.86-1.31)	0.61	0.75

6q14	rs17530068	82193109	A	G	1.13 (0.88-1.44)	0.34	0.81
6q25.1	rs3757318	151914113	G	A	1.06 (0.72-1.56)	0.78	0.64
6q25.1	rs2046210	151948366	G	A	1.02 (0.83-1.25)	0.87	0.70
7q35	rs720475	144074929	G	A	0.85 (0.67-1.08)	0.19	0.27
8p12	rs9693444	29509616	C	A	0.98 (0.79-1.21)	0.86	0.92
8q21.11	rs6472903	76230301	A	C	0.77 (0.55-1.06)	0.11	0.14
8q21.11	rs2943559	76417937	A	G	1.15 (0.81-1.63)	0.44	0.75
8q24.21	rs11780156	129194641	G	A	0.79 (0.60-1.03)	0.08	0.12
8q24.21	rs13281615	128355618	A	G	0.94 (0.76-1.16)	0.55	0.28
9p21.3	rs1011970	22062134	C	A	1.01 (0.78-1.31)	0.95	0.86
9q31.2	rs10759243	110306115	C	A	0.96 (0.76-1.21)	0.73	0.72
9q31.2	rs865686	110888478	A	C	1.00 (0.80-1.24)	0.97	0.96
10p12.31	rs7072776	22032942	G	A	1.07 (0.86-1.34)	0.53	0.55
10p12.31	rs11814448	22315843	A	C			
10q21.2	rs10995190	64278682	G	A	1.02 (0.75-1.39)	0.89	0.59
10q22.3	rs704010	80841148	G	A	0.86 (0.69-1.06)	0.15	0.13
10q25.2	rs7904519	114773927	A	G	1.14 (0.93-1.40)	0.21	0.31
10q26.12	rs2981579	123337335	G	A	1.25 (1.02-1.53)	0.03	0.99
10q26.12	rs11199914	123093901	G	A	1.05 (0.85-1.29)	0.65	0.34
11p15.5	rs3817198	1909006	A	G	1.10 (0.89-1.38)	0.38	0.89
11q13.1	rs3903072	65583066	C	A	1.06 (0.85-1.32)	0.61	0.54
11q13.3	rs554219	69331642	C	G	1.06 (0.79-1.43)	0.69	0.86
11q13.3	c11_pos69088342	69379161	C	A	1.04 (0.68-1.59)	0.85	0.84
11q13.3	rs494406	69344241	G	A	0.98 (0.78-1.22)	0.85	0.50
11q24.3	rs11820646	129461171	G	A	1.04 (0.85-1.27)	0.69	0.18
12p13.1	rs12422552	14413931	G	C	1.24 (0.99-1.55)	0.06	0.04
12p11.22	rs10771399	28155080	A	G	0.96 (0.69-1.36)	0.84	0.63
12q22	rs17356907	96027759	A	G	1.13 (0.92-1.38)	0.26	0.25
12q24.21	rs1292011	115836522	A	G	0.82 (0.68-1.00)	0.05	0.26

13q13.1	rs11571833	32972626	T	A			
14q13.3	rs2236007	37132769	G	A	1.2 (0.95-1.52)	0.13	0.12
14q24.1	rs2588809	68660428	G	A	0.94 (0.71-1.24)	0.67	0.64
14q24.1	rs999737	69034682	G	A	0.91 (0.72-1.15)	0.44	0.62
14q32.11	rs941764	91841069	A	G	0.87 (0.70-1.08)	0.21	0.13
16q12.1_1	rs3803662	52586341	G	A	1.57 (1.29-1.92)	**$8.2x10^{-6}$**	0.01
16q12.1_2	rs11075995	53855291	A	T	0.88 (0.69-1.13)	0.33	0.25
16q12.1_2	rs17817449	53813367	A	C	0.94 (0.75-1.17)	0.57	0.97
16q23.2	rs13329835	80650805	A	G	1.03 (0.82-1.29)	0.79	1.00
17q22	rs6504950	53056471	G	A	0.98 (0.78-1.21)	0.82	0.52
18q11.2	rs527616	24337424	C	G	1.10 (0.91-1.32)	0.34	0.17
18q11.2	rs1436904	22824665	A	C	0.87 (0.71-1.07)	0.19	0.31
19p13.11	rs8170	17389704	G	A	0.84 (0.62-1.12)	0.23	0.32
19p13.11	rs4808801	18571141	A	G	0.88 (0.72-1.09)	0.24	0.34
19q13.31	rs3760982	44286513	G	A	1.12 (0.92-1.36)	0.25	0.51
21q21.1	rs2823093	16520832	G	A	1.17 (0.92-1.47)	0.19	0.05
22q12.2	rs132390	29621477	A	G	0.87 (0.48-1.58)	0.65	0.33
22q13.1	rs6001930	40876234	A	G	1.04 (0.72-1.51)	0.83	0.85

[#] Reference and effect allele

Table S9 Associations of 74 previously reported breast cancer susceptibility variants with breast cancer by tumor grade *BRCA1* and *BRCA2* mutation carriers. Associations with p<0.01 in bold.

					***BRCA1* carriers**					***BRCA2* carriers**				
					**grade 1 or 2**		**grade 3**			**grade 1 or 2**		**grade 3**		
**Locus**	**SNP**	**Position**	**Ref[#]**	**Eff[#]**	**HR (95%CI)**	**p**	**HR (95%CI)**	**p**	**p_{het}**	**HR (95%CI)**	**p**	**HR (95%CI)**	**p**	**p_{het}**
1p36.22	rs616488	10566215	A	G	1.02 (0.91-1.13)	0.76	0.95 (0.90-1.00)	0.05	0.28	1.00 (0.90-1.10)	0.94	0.95 (0.87-1.05)	0.34	0.58
1p13.2	rs12022378	114448389	G	A	1.06 (0.92-1.22)	0.42	1.03 (0.96-1.10)	0.39	0.73	1.03 (0.91-1.16)	0.69	1.04 (0.92-1.18)	0.50	0.86
1p11.2	rs11249433	121280613	A	G	0.98 (0.88-1.09)	0.66	1.00 (0.95-1.05)	0.94	0.72	1.01 (0.92-1.10)	0.85	1.10 (1.00-1.20)	0.04	0.22
1q32.1_1	rs6678914	202187176	G	A	0.99 (0.89-1.10)	0.87	0.98 (0.93-1.03)	0.42	0.85	1.06 (0.97-1.17)	0.20	1.02 (0.93-1.12)	0.64	0.59
1q32.1_2	rs4245739	204518842	A	C	1.00 (0.89-1.12)	0.99	1.12 (1.07-1.18)	**$1.1x10^{-5}$**	0.08	0.90 (0.81-1.00)	0.04	1.03 (0.93-1.14)	0.55	0.09
2p24.1	rs12710696	19320803	G	A	1.05 (0.95-1.17)	0.33	1.01 (0.96-1.06)	0.79	0.46	0.93 (0.85-1.02)	0.13	1.08 (0.99-1.18)	0.09	0.03
2q14.2	rs4849887	121245122	G	A	0.87 (0.73-1.04)	0.13	1.06 (0.99-1.15)	0.11	0.06	0.95 (0.81-1.10)	0.48	1.01 (0.87-1.17)	0.92	0.60
2q31.1	rs2016394	172972971	G	A	0.98 (0.88-1.10)	0.77	1.02 (0.97-1.07)	0.41	0.57	0.93 (0.85-1.02)	0.12	1.05 (0.96-1.15)	0.30	0.09
2q31.1	rs1550623	174212894	A	G	0.95 (0.81-1.11)	0.50	1.02 (0.96-1.09)	0.50	0.40	0.92 (0.81-1.03)	0.16	1.02 (0.90-1.15)	0.78	0.24
2q35	rs13387042	217905832	A	G	0.98 (0.89-1.09)	0.73	0.98 (0.93-1.03)	0.39	0.96	0.93 (0.85-1.02)	0.11	1.02 (0.93-1.11)	0.69	0.17
2q35	rs16857609	218296508	G	A	1.05 (0.93-1.18)	0.45	1.05 (0.99-1.10)	0.10	0.98	0.91 (0.82-1.01)	0.09	1.02 (0.92-1.13)	0.69	0.17
3p26.1	rs6762644	4742276	A	G	1.16 (1.04-1.29)	**$7.2x10^{-3}$**	1.00 (0.96-1.06)	0.85	0.03	0.95 (0.86-1.05)	0.32	1.01 (0.92-1.10)	0.90	0.45
3p24.1	rs4973768	27416013	G	A	1.07 (0.97-1.19)	0.16	1.00 (0.95-1.04)	0.91	0.20	1.07 (0.98-1.18)	0.15	1.11 (1.02-1.22)	0.02	0.60
3p24.1	rs12493607	30682939	C	G	0.99 (0.89-1.11)	0.92	0.99 (0.95-1.04)	0.82	1.00	1.01 (0.92-1.12)	0.82	0.97 (0.88-1.07)	0.53	0.57
4q24	rs9790517	106084778	G	A	0.95 (0.84-1.07)	0.40	0.99 (0.94-1.05)	0.85	0.51	1.00 (0.90-1.12)	0.93	0.93 (0.84-1.04)	0.22	0.39
4q34.1	rs6828523	175846426	C	A	1.01 (0.85-1.19)	0.93	1.03 (0.96-1.12)	0.40	0.80	1.00 (0.86-1.17)	0.97	0.95 (0.82-1.10)	0.54	0.67
5p15.33	rs10069690	1279790	G	A	1.15 (1.02-1.29)	0.02	1.22 (1.16-1.29)	**$2.4x10^{-13}$**	0.39	1.05 (0.95-1.17)	0.35	1.17 (1.06-1.30)	**$1.9x10^{-3}$**	0.17
5p15.33	rs7725218	1282414	G	A	1.04 (0.93-1.17)	0.46	1.09 (1.04-1.15)	**$5.4x10^{-4}$**	0.51	1.06 (0.96-1.17)	0.23	1.05 (0.95-1.15)	0.33	0.88
5p15.33	rs2736108	1297488	G	A	0.94 (0.84-1.06)	0.32	0.88 (0.83-0.92)	**$1.3x10^{-6}$**	0.28	0.89 (0.81-0.99)	0.04	0.97 (0.88-1.07)	0.57	0.30
5p12	rs10941679	44706498	A	G	1.04 (0.92-1.18)	0.52	0.97 (0.92-1.03)	0.36	0.36	1.09 (0.98-1.21)	0.11	1.05 (0.95-1.16)	0.37	0.64
5q11.2	rs889312	56031884	A	C	0.99 (0.88-1.11)	0.82	1.02 (0.97-1.08)	0.41	0.60	1.00 (0.9-1.10)	0.98	1.08 (0.98-1.19)	0.11	0.29
5q11.3	rs10472076	58184061	A	G	1.03 (0.92-1.14)	0.62	1.00 (0.95-1.05)	1.00	0.67	0.97 (0.88-1.07)	0.54	1.00 (0.91-1.10)	0.98	0.70
5q11.3	rs1353747	58337481	A	C	0.91 (0.76-1.10)	0.34	0.99 (0.91-1.07)	0.81	0.45	0.96 (0.82-1.12)	0.56	0.97 (0.83-1.14)	0.72	0.89
5q33.3	rs1432679	158244083	A	G	1.00 (0.90-1.10)	0.94	1.06 (1.01-1.11)	0.02	0.31	0.96 (0.88-1.06)	0.43	1.03 (0.95-1.13)	0.46	0.31
6p25.3	rs11242675	1318878	A	G	1.01 (0.91-1.14)	0.79	0.95 (0.90-1.00)	0.03	0.28	1.00 (0.91-1.10)	0.94	0.99 (0.90-1.09)	0.83	0.86

6p24.3	rs9348512	10456706	C	A	0.93 (0.83-1.04)	0.19	1.01 (0.96-1.06)	0.70	0.20	0.82 (0.74-0.90)	**2.1x10^{-5}**	0.88 (0.81-0.97)	**9.1x10^{-3}**	0.27
6p23	rs204247	13722523	A	G	1.08 (0.98-1.19)	0.14	0.98 (0.93-1.03)	0.44	0.11	1.04 (0.95-1.14)	0.36	1.13 (1.04-1.24)	**6.1x10^{-3}**	0.24
6q14	rs17530068	82193109	A	G	1.01 (0.90-1.14)	0.84	1.03 (0.98-1.09)	0.27	0.78	1.08 (0.98-1.20)	0.14	1.12 (1.01-1.24)	0.03	0.67
6q25.1	rs3757318	151914113	G	A	1.04 (0.85-1.28)	0.68	1.24 (1.14-1.35)	**4.1x10^{-7}**	0.14	1.10 (0.93-1.30)	0.28	1.20 (1.02-1.41)	0.02	0.48
6q25.1	rs2046210	151948366	G	A	1.12 (1.01-1.25)	0.04	1.17 (1.12-1.23)	**2.2x10^{-10}**	0.51	1.03 (0.93-1.13)	0.58	1.09 (0.99-1.20)	0.07	0.42
7q35	rs720475	144074929	G	A	0.92 (0.82-1.05)	0.21	0.99 (0.94-1.05)	0.78	0.34	1.04 (0.94-1.16)	0.41	0.92 (0.83-1.02)	0.11	0.11
8p12	rs9693444	29509616	C	A	1.06 (0.95-1.18)	0.30	0.99 (0.94-1.04)	0.75	0.30	1.00 (0.91-1.10)	0.98	0.97 (0.88-1.06)	0.48	0.64
8q21.11	rs6472903	76230301	A	C	0.92 (0.80-1.06)	0.27	1.03 (0.97-1.10)	0.38	0.19	0.99 (0.87-1.13)	0.91	0.95 (0.84-1.08)	0.46	0.69
8q21.11	rs2943559	76417937	A	G	1.27 (1.07-1.52)	**7.1x10^{-3}**	1.00 (0.92-1.10)	0.94	0.02	1.08 (0.92-1.27)	0.35	1.12 (0.96-1.31)	0.15	0.76
8q24.21	rs11780156	129194641	G	A	0.81 (0.71-0.93)	**3.6x10^{-3}**	0.98 (0.93-1.04)	0.60	0.02	1.00 (0.89-1.12)	0.99	0.95 (0.85-1.06)	0.33	0.53
8q24.21	rs13281615	128355618	A	G	1.04 (0.93-1.15)	0.51	1.01 (0.96-1.06)	0.68	0.68	0.98 (0.89-1.07)	0.63	1.10 (1.01-1.21)	0.04	0.09
9p21.3	rs1011970	22062134	C	A	0.96 (0.82-1.11)	0.54	1.04 (0.97-1.11)	0.26	0.33	1.06 (0.95-1.20)	0.30	0.98 (0.87-1.10)	0.77	0.38
9q31.2	rs10759243	110306115	C	A	1.00 (0.89-1.12)	0.95	0.98 (0.93-1.03)	0.45	0.80	1.02 (0.92-1.13)	0.75	0.98 (0.89-1.09)	0.76	0.68
9q31.2	rs865686	110888478	A	C	0.97 (0.87-1.09)	0.63	1.00 (0.95-1.05)	0.95	0.70	0.98 (0.89-1.08)	0.64	1.00 (0.91-1.10)	0.99	0.77
10p12.31	rs7072776	22032942	G	A	1.04 (0.93-1.16)	0.50	0.98 (0.93-1.03)	0.35	0.33	1.00 (0.91-1.10)	0.99	1.00 (0.91-1.10)	0.95	0.97
10p12.31	rs11814448	22315843	A	C										
10q21.2	rs10995190	64278682	G	A	1.02 (0.88-1.18)	0.84	0.98 (0.92-1.05)	0.58	0.69	0.96 (0.83-1.10)	0.53	0.94 (0.82-1.07)	0.32	0.83
10q22.3	rs704010	80841148	G	A	1.08 (0.97-1.21)	0.15	1.00 (0.95-1.05)	1.00	0.22	1.01 (0.93-1.11)	0.77	0.99 (0.90-1.08)	0.82	0.73
10q25.2	rs7904519	114773927	A	G	1.11 (1.00-1.23)	0.04	1.09 (1.04-1.15)	**2.1x10^{-4}**	0.79	1.03 (0.94-1.13)	0.50	1.01 (0.92-1.10)	0.85	0.75
10q26.12	rs2981579	123337335	G	A	1.15 (1.03-1.28)	0.01	0.96 (0.91-1.01)	0.09	**4.1x10^{-3}**	1.31 (1.20-1.44)	**4.8x10^{-9}**	1.17 (1.07-1.28)	**7.1x10^{-4}**	0.09
10q26.12	rs11199914	123093901	G	A	0.99 (0.88-1.10)	0.82	1.03 (0.98-1.09)	0.20	0.48	0.94 (0.85-1.03)	0.19	0.97 (0.88-1.07)	0.56	0.63
11p15.5	rs3817198	1909006	A	G	1.12 (1.00-1.25)	0.06	1.07 (1.02-1.12)	**9.8x10^{-3}**	0.52	1.05 (0.95-1.16)	0.32	1.18 (1.07-1.29)	**7.0x10^{-4}**	0.13
11q13.1	rs3903072	65583066	C	A	0.98 (0.88-1.08)	0.65	1.00 (0.95-1.04)	0.89	0.74	1.00 (0.91-1.10)	0.97	0.97 (0.88-1.05)	0.43	0.59
11q13.3	rs554219	69331642	C	G	1.06 (0.90-1.24)	0.49	1.02 (0.95-1.10)	0.50	0.74	1.12 (0.99-1.28)	0.08	1.06 (0.93-1.21)	0.37	0.58
11q13.3	c11_pos69088342	69379161	C	A	1.00 (0.80-1.25)	0.99	1.04 (0.94-1.16)	0.41	0.74	1.09 (0.91-1.32)	0.35	1.05 (0.87-1.27)	0.58	0.80
11q13.3	rs494406	69344241	G	A	0.98 (0.87-1.11)	0.76	1.03 (0.97-1.09)	0.31	0.50	1.02 (0.93-1.13)	0.64	1.07 (0.97-1.19)	0.15	0.53
11q24.3	rs11820646	129461171	G	A	0.89 (0.80-0.99)	0.03	0.94 (0.89-0.99)	**0.01**	0.35	0.99 (0.90-1.08)	0.79	0.85 (0.77-0.93)	**5.0x10^{-4}**	0.03
12p13.1	rs12422552	14413931	G	C	0.95 (0.84-1.07)	0.39	1.03 (0.98-1.09)	0.29	0.26	0.98 (0.89-1.09)	0.74	1.01 (0.91-1.11)	0.87	0.75
12p11.22	rs10771399	28155080	A	G	0.94 (0.79-1.12)	0.47	0.83 (0.76-0.90)	**4.8x10^{-6}**	0.24	0.80 (0.68-0.94)	**6.6x10^{-3}**	0.97 (0.85-1.12)	0.69	0.10

12q22	rs17356907	96027759	A	G	0.97 (0.86-1.09)	0.61	0.95 (0.90-1.00)	0.04	0.72	1.04 (0.94-1.14)	0.44	0.95 (0.86-1.05)	0.30	0.23
12q24.21	rs1292011	115836522	A	G	0.87 (0.78-0.97)	0.01	1.04 (0.99-1.09)	0.14	**$6.1x10^{-3}$**	0.86 (0.79-0.95)	**$1.8x10^{-3}$**	0.97 (0.89-1.06)	0.51	0.09
13q13.1	rs11571833	32972626	T	A										
14q13.3	rs2236007	37132769	G	A	0.94 (0.83-1.07)	0.37	0.97 (0.92-1.03)	0.36	0.68	1.00 (0.90-1.12)	0.97	1.00 (0.89-1.12)	0.96	0.96
14q24.1	rs2588809	68660428	G	A	0.95 (0.83-1.09)	0.49	0.96 (0.90-1.02)	0.18	0.95	0.92 (0.82-1.04)	0.19	1.10 (0.98-1.23)	0.11	0.06
14q24.1	rs999737	69034682	G	A	1.01 (0.89-1.14)	0.92	0.95 (0.89-1.00)	0.07	0.42	0.96 (0.86-1.07)	0.49	0.97 (0.87-1.08)	0.58	0.93
14q32.11	rs941764	91841069	A	G	1.05 (0.94-1.18)	0.34	1.02 (0.97-1.07)	0.53	0.58	1.05 (0.96-1.15)	0.31	1.01 (0.92-1.11)	0.83	0.59
16q12.1_1	rs3803662	52586341	G	A	1.05 (0.94-1.18)	0.40	1.06 (1.01-1.12)	0.03	0.89	1.20 (1.09-1.33)	**$2.1x10^{-4}$**	1.26 (1.15-1.39)	**$1.8x10^{-6}$**	0.54
16q12.1_2	rs11075995	53855291	A	T	1.01 (0.89-1.14)	0.86	1.01 (0.96-1.07)	0.69	1.00	1.05 (0.94-1.16)	0.41	1.00 (0.90-1.11)	1.00	0.59
16q12.1_2	rs17817449	53813367	A	C	0.88 (0.79-0.99)	0.03	0.97 (0.92-1.01)	0.17	0.17	0.96 (0.87-1.05)	0.35	0.93 (0.84-1.02)	0.11	0.69
16q23.2	rs13329835	80650805	A	G	0.99 (0.87-1.12)	0.85	1.06 (1.00-1.12)	0.04	0.34	1.07 (0.97-1.19)	0.17	1.00 (0.90-1.10)	0.93	0.32
17q22	rs6504950	53056471	G	A	1.02 (0.9-1.14)	0.79	0.98 (0.93-1.04)	0.48	0.61	1.03 (0.93-1.14)	0.55	1.05 (0.95-1.16)	0.31	0.78
18q11.2	rs527616	24337424	C	G	1.12 (1.00-1.24)	0.04	0.96 (0.91-1.01)	0.09	0.01	0.95 (0.87-1.04)	0.26	0.98 (0.89-1.07)	0.65	0.64
18q11.2	rs1436904	22824665	A	C	1.03 (0.92-1.14)	0.61	0.98 (0.93-1.03)	0.41	0.44	0.96 (0.88-1.06)	0.44	0.94 (0.86-1.03)	0.20	0.75
19p13.11	rs8170	17389704	G	A	1.14 (1.00-1.30)	0.05	1.20 (1.13-1.27)	**$4.6x10^{-9}$**	0.51	0.98 (0.86-1.10)	0.70	0.96 (0.86-1.08)	0.53	0.89
19p13.11	rs4808801	18571141	A	G	0.97 (0.86-1.08)	0.54	0.98 (0.94-1.03)	0.53	0.78	0.99 (0.90-1.09)	0.82	0.95 (0.87-1.05)	0.33	0.62
19q13.31	rs3760982	44286513	G	A	0.98 (0.88-1.09)	0.74	1.05 (1.00-1.10)	0.05	0.30	1.06 (0.97-1.15)	0.22	1.04 (0.96-1.14)	0.33	0.87
21q21.1	rs2823093	16520832	G	A	0.86 (0.76-0.97)	0.01	0.98 (0.93-1.03)	0.39	0.07	0.89 (0.80-1.00)	0.04	0.99 (0.89-1.10)	0.81	0.23
22q12.2	rs132390	29621477	A	G	1.15 (0.89-1.50)	0.29	0.94 (0.82-1.08)	0.40	0.20	1.20 (0.93-1.55)	0.17	1.18 (0.93-1.49)	0.18	0.92
22q13.1	rs6001930	40876234	A	G	1.16 (0.98-1.37)	0.08	1.05 (0.97-1.13)	0.24	0.31	1.12 (0.97-1.29)	0.13	0.91 (0.78-1.06)	0.23	0.08

Reference and effect allele

Table S10 Associations of 74 previously reported breast cancer susceptibility variants with breast cancer by nodal involvement in *BRCA1* and *BRCA2* mutation carriers. Associations with p<0.01 are in bold.

					***BRCA1* carriers**					***BRCA2* carriers**				
					**node-negative**		**node-positive**			**node-negative**		**node-positive**		
**Locus**	**SNP**	**Position**	**Ref#**	**Eff#**	**HR (95%CI)**	**p**	**HR (95%CI)**	**p**	**Phet**	**HR (95%CI)**	**p**	**HR (95%CI)**	**p**	**Phet**
1p36.22	rs616488	10566215	A	G	0.92 (0.87-0.98)	**$5.9x10^{-3}$**	1.05 (0.97-1.14)	0.26	0.02	0.96 (0.88-1.05)	0.35	1.00 (0.90-1.10)	0.93	0.61
1p13.2	rs12022378	114448389	G	A	1.07 (0.99-1.15)	0.07	0.97 (0.87-1.08)	0.57	0.17	0.97 (0.86-1.09)	0.58	1.12 (0.99-1.27)	0.06	0.09
1p11.2	rs11249433	121280613	A	G	0.99 (0.94-1.05)	0.75	1.00 (0.92-1.09)	0.98	0.89	1.04 (0.96-1.13)	0.35	1.07 (0.98-1.17)	0.15	0.66
1q32.1_1	rs6678914	202187176	G	A	0.98 (0.93-1.04)	0.55	0.98 (0.90-1.06)	0.62	0.94	1.05 (0.97-1.14)	0.23	1.03 (0.94-1.13)	0.55	0.75
1q32.1_2	rs4245739	204518842	A	C	1.08 (1.02-1.14)	0.01	1.14 (1.05-1.24)	**$2.4x10^{-3}$**	0.30	0.95 (0.87-1.04)	0.28	0.98 (0.89-1.09)	0.73	0.66
2p24.1	rs12710696	19320803	G	A	1.01 (0.96-1.07)	0.69	1.03 (0.95-1.11)	0.52	0.77	1.02 (0.94-1.11)	0.58	0.98 (0.89-1.07)	0.65	0.49
2q14.2	rs4849887	121245122	G	A	1.04 (0.95-1.13)	0.38	0.99 (0.87-1.13)	0.90	0.59	1.06 (0.93-1.21)	0.36	0.87 (0.73-1.02)	0.09	0.07
2q31.1	rs2016394	172972971	G	A	1.04 (0.98-1.1)	0.16	0.96 (0.88-1.04)	0.35	0.15	1.03 (0.95-1.12)	0.49	0.94 (0.85-1.03)	0.19	0.17
2q31.1	rs1550623	174212894	A	G	0.95 (0.88-1.03)	0.20	1.13 (1.01-1.26)	0.03	0.02	0.91 (0.82-1.02)	0.12	1.04 (0.92-1.18)	0.54	0.13
2q35	rs13387042	217905832	A	G	0.98 (0.93-1.03)	0.34	0.99 (0.91-1.07)	0.80	0.77	1.00 (0.92-1.08)	0.94	0.94 (0.86-1.03)	0.21	0.39
2q35	rs16857609	218296508	G	A	1.05 (0.99-1.11)	0.10	1.04 (0.95-1.14)	0.43	0.83	0.96 (0.87-1.05)	0.37	0.98 (0.88-1.09)	0.73	0.75
3p26.1	rs6762644	4742276	A	G	1.04 (0.98-1.09)	0.21	1.03 (0.95-1.12)	0.45	0.96	0.98 (0.90-1.07)	0.70	0.97 (0.89-1.07)	0.60	0.90
3p24.1	rs4973768	27416013	G	A	1.02 (0.97-1.07)	0.47	1.00 (0.92-1.08)	0.96	0.67	1.05 (0.96-1.14)	0.27	1.15 (1.05-1.26)	**$3.2x10^{-3}$**	0.15
3p24.1	rs12493607	30682939	C	G	1.0 (0.95-1.06)	0.92	0.98 (0.90-1.06)	0.59	0.63	0.96 (0.88-1.05)	0.34	1.03 (0.94-1.14)	0.53	0.28
4q24	rs9790517	106084778	G	A	0.95 (0.89-1.01)	0.13	1.05 (0.96-1.16)	0.26	0.09	0.99 (0.89-1.09)	0.78	0.95 (0.84-1.06)	0.34	0.61
4q34.1	rs6828523	175846426	C	A	1.05 (0.97-1.14)	0.22	0.98 (0.86-1.12)	0.76	0.4	1.00 (0.88-1.14)	0.97	0.95 (0.81-1.11)	0.49	0.60
5p15.33	rs10069690	1279790	G	A	1.19 (1.12-1.26)	**$6.2x10^{-9}$**	1.24 (1.13-1.35)	**$2.0x10^{-6}$**	0.5	1.11 (1.01-1.21)	0.03	1.12 (1.01-1.25)	0.03	0.86
5p15.33	rs7725218	1282414	G	A	1.06 (1.01-1.12)	0.03	1.12 (1.03-1.22)	**$8.9x10^{-3}$**	0.36	1.04 (0.96-1.14)	0.35	1.07 (0.97-1.18)	0.18	0.70
5p15.33	rs2736108	1297488	G	A	0.88 (0.83-0.94)	**$2.8x10^{-5}$**	0.91 (0.83-0.99)	0.03	0.59	0.96 (0.88-1.05)	0.40	0.90 (0.81-1.00)	0.05	0.36
5p12	rs10941679	44706498	A	G	0.98 (0.92-1.04)	0.47	1.01 (0.92-1.11)	0.85	0.6	1.04 (0.95-1.14)	0.36	1.1 (0.99-1.22)	0.07	0.47
5q11.2	rs889312	56031884	A	C	1.03 (0.98-1.10)	0.25	0.98 (0.89-1.07)	0.59	0.31	1.02 (0.94-1.12)	0.60	1.06 (0.96-1.18)	0.24	0.60
5q11.3	rs10472076	58184061	A	G	1.02 (0.97-1.08)	0.47	0.98 (0.90-1.06)	0.56	0.4	0.96 (0.88-1.05)	0.36	1.02 (0.92-1.12)	0.74	0.41
5q11.3	rs1353747	58337481	A	C	0.99 (0.90-1.08)	0.79	0.95 (0.82-1.09)	0.44	0.64	0.96 (0.84-1.11)	0.61	0.96 (0.82-1.13)	0.65	1.00
5q33.3	rs1432679	158244083	A	G	1.04 (0.99-1.10)	0.10	1.05 (0.97-1.14)	0.22	0.93	1.01 (0.93-1.09)	0.81	0.98 (0.90-1.08)	0.73	0.68
6p25.3	rs11242675	1318878	A	G	0.99 (0.93-1.04)	0.65	0.90 (0.83-0.99)	0.02	0.12	1.04 (0.96-1.14)	0.33	0.94 (0.85-1.03)	0.20	0.13

6p24.3	rs9348512	10456706	C	A	1.00 (0.95-1.06)	0.95	0.98 (0.90-1.06)	0.57	0.63	0.84 (0.77-0.91)	**3.9x10^{-5}**	0.87 (0.79-0.96)	**4.2x10^{-3}**	0.58
6p23	rs204247	13722523	A	G	1.00 (0.95-1.05)	0.98	1.00 (0.93-1.08)	0.96	0.95	1.05 (0.97-1.14)	0.21	1.14 (1.03-1.25)	**7.2x10^{-3}**	0.27
6q14	rs17530068	82193109	A	G	1.04 (0.98-1.11)	0.18	1.00 (0.91-1.10)	1.00	0.48	1.11 (1.01-1.22)	0.03	1.09 (0.98-1.22)	0.11	0.84
6q25.1	rs3757318	151914113	G	A	1.13 (1.02-1.24)	0.01	1.35 (1.19-1.55)	**7.4x10^{-6}**	0.04	1.10 (0.95-1.27)	0.22	1.22 (1.04-1.43)	0.02	0.37
6q25.1	rs2046210	151948366	G	A	1.14 (1.08-1.21)	**1.4x10^{-6}**	1.21 (1.11-1.31)	**6.4x10^{-6}**	0.32	1.08 (0.99-1.17)	0.09	1.04 (0.94-1.15)	0.42	0.63
7q35	rs720475	144074929	G	A	0.99 (0.93-1.05)	0.75	0.95 (0.87-1.05)	0.33	0.55	1.00 (0.91-1.10)	0.99	0.95 (0.86-1.06)	0.38	0.53
8p12	rs9693444	29509616	C	A	1.00 (0.95-1.06)	0.91	1.01 (0.93-1.10)	0.83	0.92	0.97 (0.89-1.05)	0.43	1.01 (0.92-1.11)	0.89	0.55
8q21.11	rs6472903	76230301	A	C	1.06 (0.99-1.13)	0.12	0.91 (0.81-1.01)	0.09	0.03	1.01 (0.90-1.12)	0.89	0.93 (0.81-1.06)	0.27	0.37
8q21.11	rs2943559	76417937	A	G	1.04 (0.94-1.14)	0.47	1.10 (0.95-1.27)	0.19	0.52	1.13 (0.98-1.3)	0.10	1.07 (0.91-1.26)	0.41	0.67
8q24.21	rs11780156	129194641	G	A	0.97 (0.91-1.04)	0.40	0.90 (0.81-1.00)	0.05	0.26	1.02 (0.93-1.13)	0.63	0.90 (0.80-1.02)	0.10	0.14
8q24.21	rs13281615	128355618	A	G	1.03 (0.98-1.09)	0.22	0.98 (0.90-1.06)	0.59	0.28	1.05 (0.97-1.14)	0.24	1.03 (0.93-1.13)	0.58	0.73
9p21.3	rs1011970	22062134	C	A	1.06 (0.98-1.14)	0.13	0.95 (0.85-1.06)	0.35	0.13	1.00 (0.90-1.12)	0.99	1.05 (0.93-1.19)	0.44	0.59
9q31.2	rs10759243	110306115	C	A	0.98 (0.93-1.04)	0.48	0.99 (0.91-1.08)	0.86	0.82	1.03 (0.94-1.13)	0.52	0.96 (0.86-1.07)	0.48	0.37
9q31.2	rs865686	110888478	A	C	0.98 (0.92-1.03)	0.42	1.03 (0.94-1.12)	0.54	0.37	1.00 (0.92-1.09)	1.00	0.97 (0.88-1.08)	0.60	0.71
10p12.31	rs7072776	22032942	G	A	0.97 (0.92-1.03)	0.35	1.02 (0.94-1.11)	0.65	0.4	1.03 (0.95-1.13)	0.48	0.95 (0.86-1.05)	0.36	0.26
10p12.31	rs11814448	22315843	A	C										
10q21.2	rs10995190	64278682	G	A	1.03 (0.96-1.11)	0.41	0.90 (0.80-1.01)	0.08	0.07	0.95 (0.85-1.08)	0.45	0.94 (0.82-1.07)	0.34	0.84
10q22.3	rs704010	80841148	G	A	1.00 (0.95-1.06)	0.92	1.04 (0.96-1.14)	0.29	0.43	1.02 (0.94-1.10)	0.64	0.98 (0.89-1.07)	0.63	0.51
10q25.2	rs7904519	114773927	A	G	1.12 (1.06-1.18)	**1.4x10^{-5}**	1.05 (0.97-1.13)	0.24	0.18	1.03 (0.95-1.12)	0.5	1.01 (0.92-1.10)	0.86	0.76
10q26.12	rs2981579	123337335	G	A	0.97 (0.92-1.02)	0.27	1.05 (0.97-1.14)	0.23	0.13	1.20 (1.10-1.30)	**2.2x10^{-5}**	1.29 (1.18-1.42)	**3.8x10^{-8}**	0.21
10q26.12	rs11199914	123093901	G	A	1.00 (0.94-1.05)	0.87	1.09 (1.00-1.18)	0.06	0.12	0.98 (0.90-1.07)	0.63	0.92 (0.84-1.02)	0.12	0.41
11p15.5	rs3817198	1909006	A	G	1.08 (1.02-1.14)	**7.0x10^{-3}**	1.07 (0.99-1.17)	0.10	0.9	1.11 (1.01-1.21)	0.02	1.12 (1.02-1.24)	0.02	0.84
11q13.1	rs3903072	65583066	C	A	0.99 (0.94-1.04)	0.72	1.00 (0.92-1.08)	0.93	0.9	0.96 (0.88-1.04)	0.31	1.02 (0.92-1.12)	0.75	0.38
11q13.3	rs554219	69331642	C	G	1.02 (0.94-1.1)	0.70	1.06 (0.94-1.20)	0.31	0.55	1.01 (0.89-1.14)	0.88	1.20 (1.06-1.37)	**5.1x10^{-3}**	0.06
11q13.3	c11_pos69088342	69379161	C	A	1.06 (0.95-1.19)	0.33	0.99 (0.83-1.18)	0.89	0.54	0.96 (0.80-1.15)	0.62	1.23 (1.03-1.48)	0.02	0.06
11q13.3	rs494406	69344241	G	A	1.02 (0.96-1.08)	0.62	1.03 (0.94-1.12)	0.56	0.84	1.03 (0.94-1.13)	0.54	1.08 (0.98-1.19)	0.14	0.51
11q24.3	rs11820646	129461171	G	A	0.94 (0.89-1.00)	0.03	0.9 (0.83-0.98)	0.01	0.4	0.91 (0.83-0.99)	0.02	0.92 (0.84-1.01)	0.10	0.81
12p13.1	rs12422552	14413931	G	C	0.99 (0.93-1.05)	0.66	1.07 (0.97-1.17)	0.16	0.19	1.02 (0.93-1.12)	0.62	0.96 (0.86-1.07)	0.45	0.40
12p11.22	rs10771399	28155080	A	G	0.83 (0.76-0.91)	**4.1x10^{-5}**	0.89 (0.78-1.01)	0.07	0.43	0.95 (0.83-1.08)	0.44	0.81 (0.68-0.96)	0.01	0.16

12q22	rs17356907	96027759	A	G	0.96 (0.90-1.01)	0.13	0.94 (0.86-1.03)	0.17	0.75	0.92 (0.85-1.01)	0.08	1.09 (0.99-1.20)	0.10	0.02
12q24.21	rs1292011	115836522	A	G	1.00 (0.95-1.06)	0.93	1.00 (0.92-1.09)	0.98	0.98	0.91 (0.84-0.99)	0.03	0.92 (0.84-1.01)	0.09	0.91
13q13.1	rs11571833	32972626	T	A										
14q13.3	rs2236007	37132769	G	A	0.97 (0.91-1.03)	0.30	0.97 (0.88-1.07)	0.50	0.99	1.02 (0.93-1.13)	0.67	0.97 (0.86-1.09)	0.62	0.53
14q24.1	rs2588809	68660428	G	A	0.95 (0.89-1.01)	0.11	0.98 (0.88-1.09)	0.70	0.6	1.06 (0.96-1.18)	0.25	0.94 (0.83-1.06)	0.33	0.16
14q24.1	rs999737	69034682	G	A	0.97 (0.91-1.03)	0.33	0.94 (0.85-1.04)	0.22	0.64	0.97 (0.88-1.07)	0.58	0.96 (0.86-1.07)	0.45	0.84
14q32.11	rs941764	91841069	A	G	1.02 (0.96-1.08)	0.55	1.04 (0.95-1.13)	0.40	0.72	1.01 (0.93-1.09)	0.89	1.06 (0.96-1.16)	0.23	0.42
16q12.1_1	rs3803662	52586341	G	A	1.00 (0.95-1.06)	0.90	1.18 (1.08-1.28)	**2.3x10^{-4}**	**4.7x10^{-3}**	1.20 (1.10-1.31)	**3.1x10^{-5}**	1.27 (1.15-1.41)	**1.7x10^{-6}**	0.41
16q12.1_2	rs11075995	53855291	A	T	1.00 (0.94-1.06)	0.88	1.04 (0.95-1.15)	0.36	0.42	1.06 (0.96-1.17)	0.23	0.97 (0.87-1.09)	0.63	0.26
16q12.1_2	rs17817449	53813367	A	C	0.93 (0.88-0.98)	**7.0x10^{-3}**	0.99 (0.91-1.07)	0.83	0.23	0.92 (0.85-1.00)	0.06	0.97 (0.88-1.06)	0.48	0.51
16q23.2	rs13329835	80650805	A	G	1.06 (0.99-1.12)	0.08	1.03 (0.93-1.13)	0.59	0.63	1.04 (0.94-1.14)	0.46	1.03 (0.93-1.15)	0.58	0.94
17q22	rs6504950	53056471	G	A	1.02 (0.96-1.08)	0.60	0.93 (0.85-1.02)	0.12	0.13	1.08 (0.99-1.18)	0.08	0.99 (0.89-1.10)	0.85	0.20
18q11.2	rs527616	24337424	C	G	1.02 (0.97-1.08)	0.38	0.92 (0.84-1.00)	0.04	0.04	0.93 (0.85-1.01)	0.07	1.02 (0.92-1.12)	0.75	0.16
18q11.2	rs1436904	22824665	A	C	0.97 (0.92-1.02)	0.24	1.03 (0.95-1.12)	0.42	0.21	0.97 (0.90-1.06)	0.53	0.93 (0.84-1.02)	0.13	0.47
19p13.11	rs8170	17389704	G	A	1.19 (1.11-1.27)	**4.6x10^{-7}**	1.18 (1.07-1.31)	**8.7x10^{-4}**	0.95	0.99 (0.89-1.10)	0.80	0.95 (0.84-1.07)	0.39	0.65
19p13.11	rs4808801	18571141	A	G	0.99 (0.93-1.04)	0.65	0.97 (0.89-1.05)	0.41	0.68	0.96 (0.88-1.04)	0.33	0.99 (0.90-1.09)	0.82	0.64
19q13.31	rs3760982	44286513	G	A	1.04 (0.99-1.10)	0.11	1.02 (0.94-1.10)	0.69	0.63	1.04 (0.96-1.13)	0.31	1.06 (0.97-1.16)	0.20	0.79
21q21.1	rs2823093	16520832	G	A	0.96 (0.90-1.02)	0.15	0.94 (0.86-1.03)	0.17	0.74	0.94 (0.86-1.04)	0.23	0.93 (0.84-1.04)	0.21	0.88
22q12.2	rs132390	29621477	A	G	0.95 (0.82-1.10)	0.49	1.06 (0.86-1.31)	0.58	0.42	1.13 (0.90-1.41)	0.29	1.26 (0.98-1.62)	0.07	0.53
22q13.1	rs6001930	40876234	A	G	1.06 (0.97-1.15)	0.19	1.09 (0.96-1.25)	0.18	0.70	0.96 (0.84-1.10)	0.56	1.08 (0.92-1.26)	0.35	0.30

[#] Reference and effect allele

Figure legends

Figure S1. QQ plots for SNP associations with ER-positive and ER-negative disease in *BRCA1* mutation carriers. The inflation coefficient was computed using SNP subsets that had been chosen for inclusion on the genotyping array independent of their associations in CIMBA *BRCA1* mutation carriers

Figure S2. QQ plots for SNP associations with ER-positive and ER-negative disease in *BRCA2* mutation carriers. The inflation coefficient was computed using SNP subsets that had been chosen for inclusion on the genotyping array independent of their associations in CIMBA *BRCA2* mutation carriers

Figure S3. Log HR/OR estimates for the association of previously reported breast cancer susceptibility SNPs with breast cancer in A. BCAC and *BRCA1* carriers, B. in BCAC and *BRCA2* carriers, C. in *BRCA1* and *BRCA2* carriers

Figure S4. Log HR estimates for the association of previously reported breast cancer susceptibility SNPs with ER-positive and ER-negative breast cancer in *BRCA1* and *BRCA2* mutation carriers

Figure S5. Log HR/OR estimates for the association of previously reported breast cancer susceptibility SNPs with ER-positive and ER-negative breast cancer breast cancer in BCAC and in *BRCA1* mutation carriers

Figure S6. Log HR/OR estimates for the association of previously reported breast cancer susceptibility SNPs with ER-positive and ER-negative breast cancer in BCAC and *BRCA2* mutation carriers

Figure S1

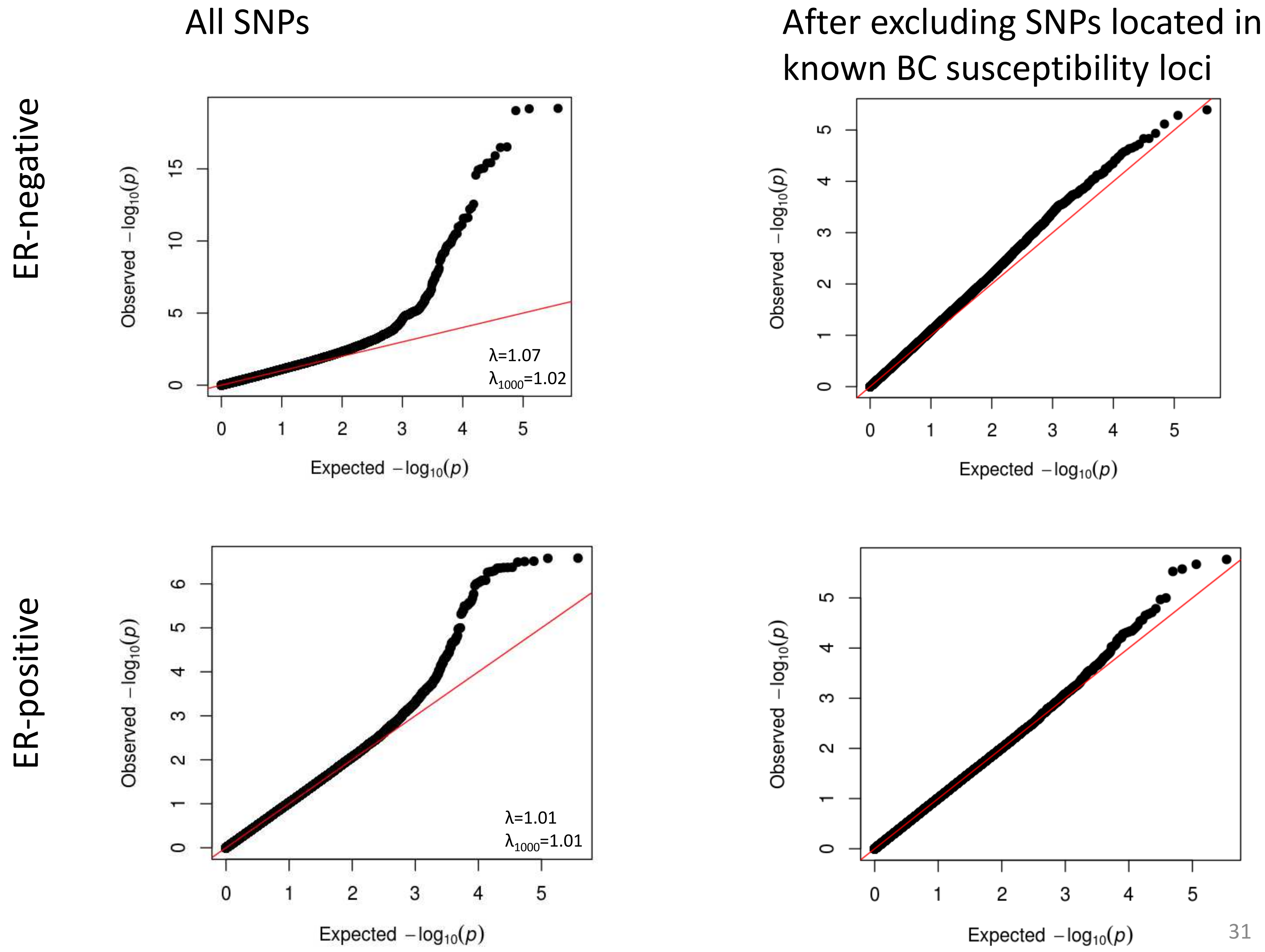
All SNPs
After excluding SNPs located in known BC susceptibility loci
ER-negative
ER-positive
Observed −log10(p)
Expected −log10(p)
λ=1.07
λ1000=1.02
λ=1.01
λ1000=1.01

Figure S2

All SNPs

After excluding SNPs located in known BC susceptibility loci

ER-negative

ER-positive

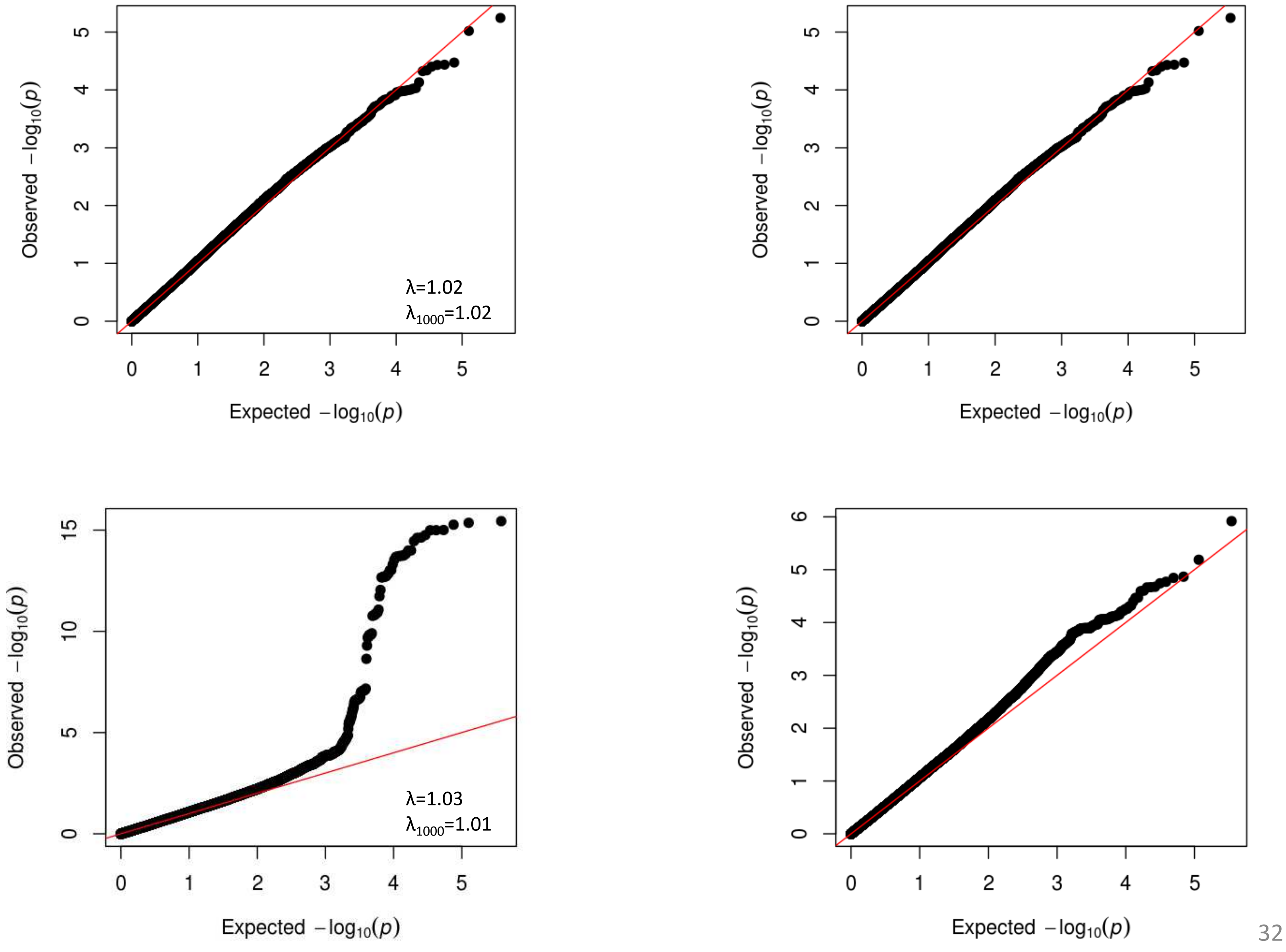

Figure S3

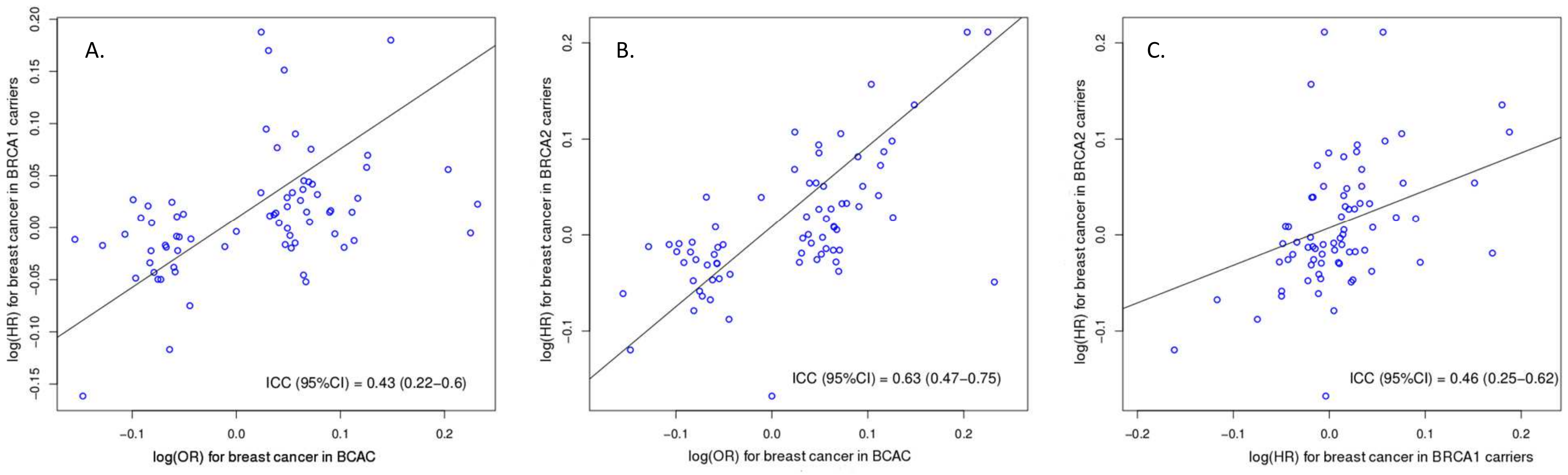
A.
log(HR) for breast cancer in BRCA1 carriers
log(OR) for breast cancer in BCAC
ICC (95%CI) = 0.43 (0.22–0.6)
B.
log(HR) for breast cancer in BRCA2 carriers
log(OR) for breast cancer in BCAC
ICC (95%CI) = 0.63 (0.47–0.75)
C.
log(HR) for breast cancer in BRCA2 carriers
log(HR) for breast cancer in BRCA1 carriers
ICC (95%CI) = 0.46 (0.25–0.62)

Figure S4

BRCA2 ER-positive | *BRCA2* ER-negative

BRCA1 ER-positive

BRCA1 ER-negative

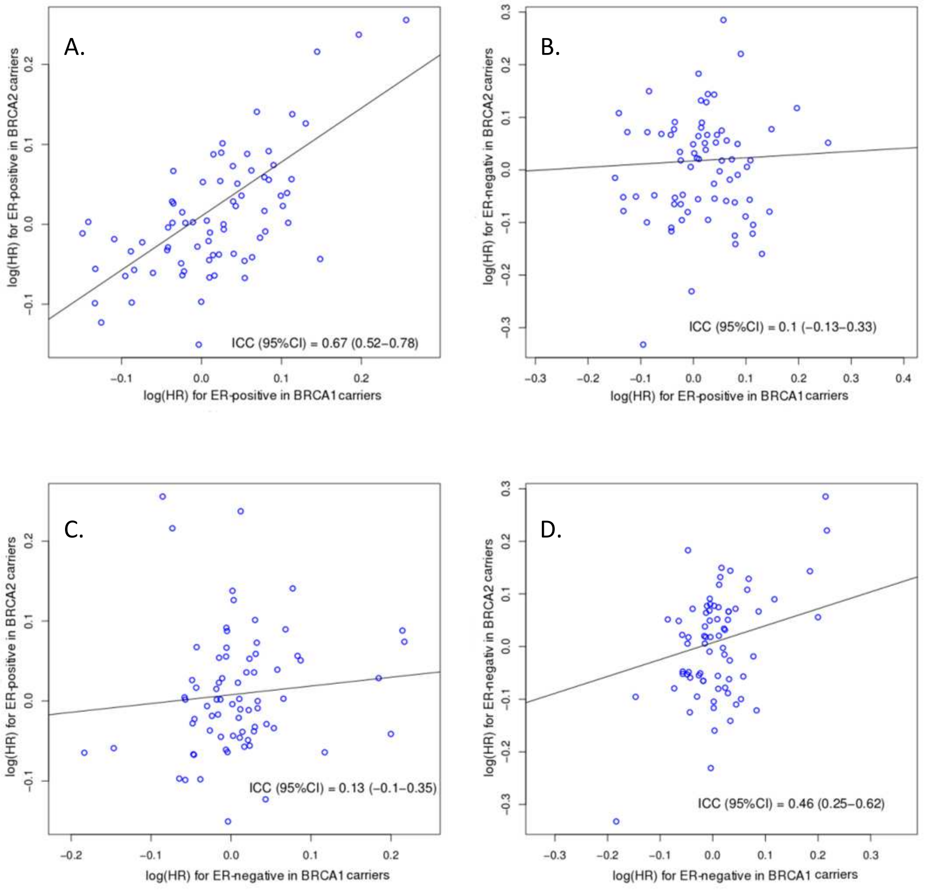

Figure S5

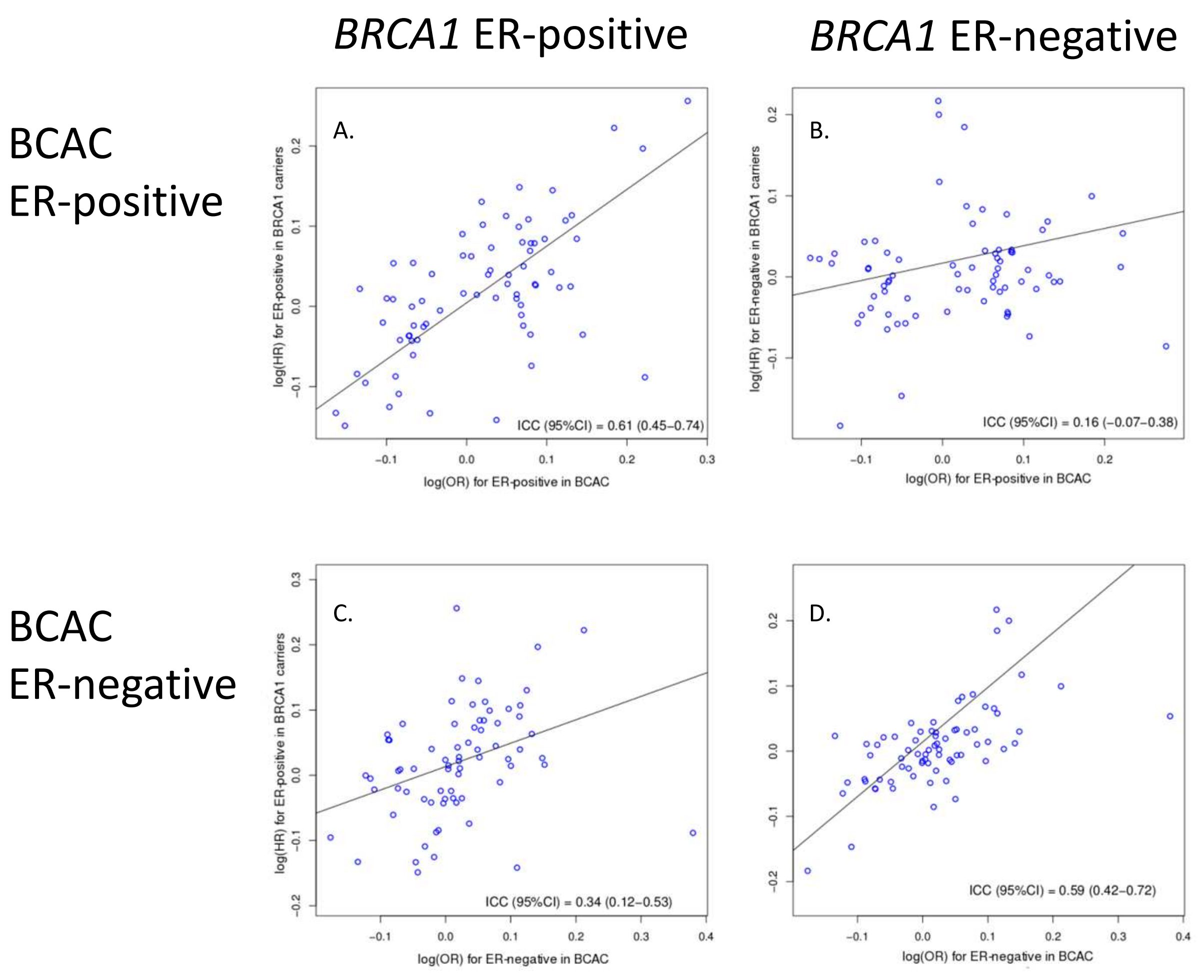

BRCA1 ER-positive
BRCA1 ER-negative
BCAC ER-positive
BCAC ER-negative
A.
B.
C.
D.
ICC (95%CI) = 0.61 (0.45–0.74)
ICC (95%CI) = 0.16 (−0.07–0.38)
ICC (95%CI) = 0.34 (0.12–0.53)
ICC (95%CI) = 0.59 (0.42–0.72)
log(HR) for ER-positive in BRCA1 carriers
log(HR) for ER-negative in BRCA1 carriers
log(OR) for ER-positive in BCAC
log(OR) for ER-negative in BCAC

Figure S6

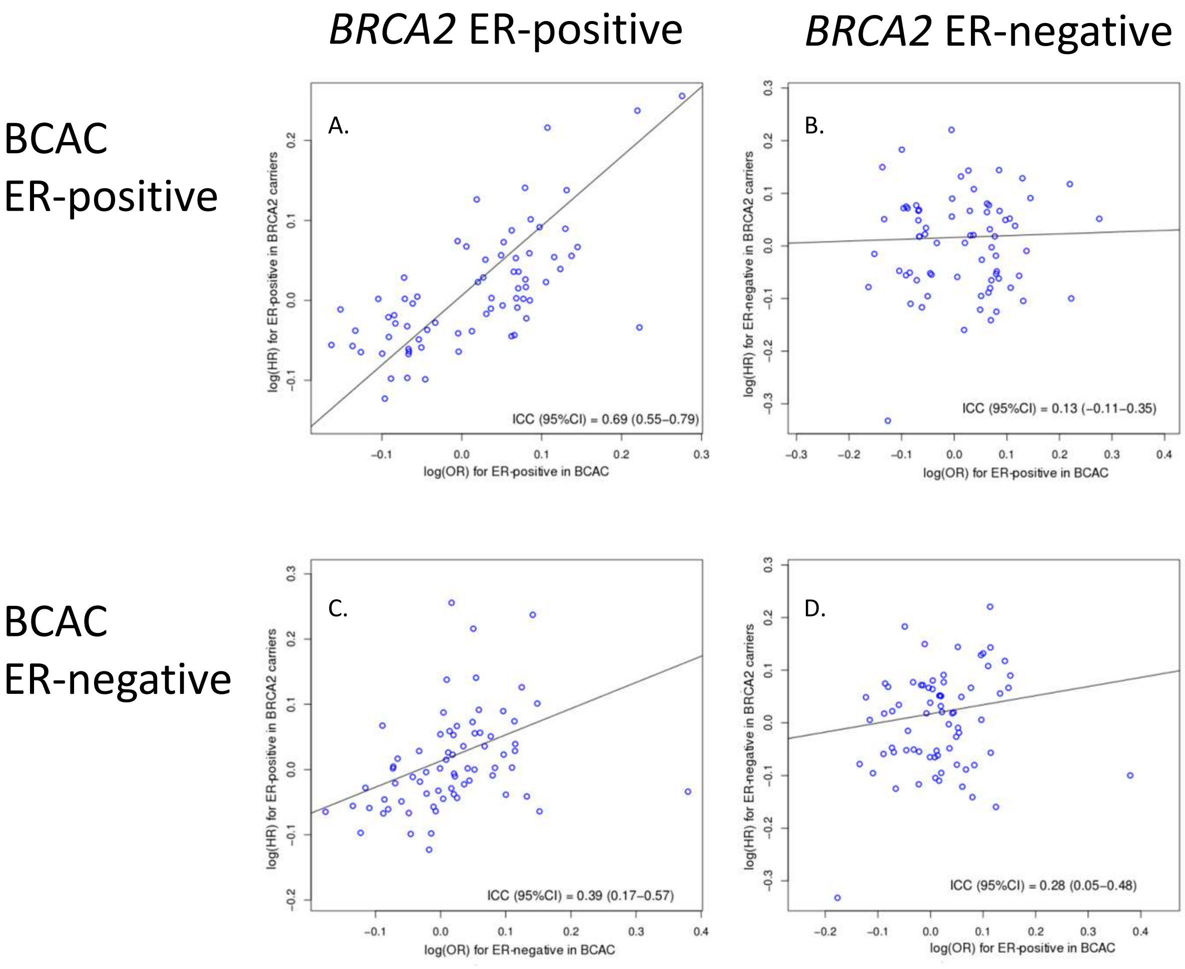

BRCA2 ER-positive
BRCA2 ER-negative
BCAC ER-positive
BCAC ER-negative
A.
ICC (95%CI) = 0.69 (0.55–0.79)
log(OR) for ER-positive in BCAC
log(HR) for ER-positive in BRCA2 carriers
B.
ICC (95%CI) = 0.13 (−0.11–0.35)
log(OR) for ER-positive in BCAC
log(HR) for ER-negative in BRCA2 carriers
C.
ICC (95%CI) = 0.39 (0.17–0.57)
log(OR) for ER-negative in BCAC
log(HR) for ER-positive in BRCA2 carriers
D.
ICC (95%CI) = 0.28 (0.05–0.48)
log(OR) for ER-positive in BCAC
log(HR) for ER-negative in BRCA2 carriers
