# Supplementary figures and images for: Associations of common breast cancer susceptibility alleles with risk of breast cancer subtypes in BRCA1 and BRCA2 mutation carriers
Source: Breast Cancer Res. 2014 Dec 31;16:3416. doi: 10.1186/s13058-014-0492-9 (PMC4406179; doi:10.1186/s13058-014-0492-9)

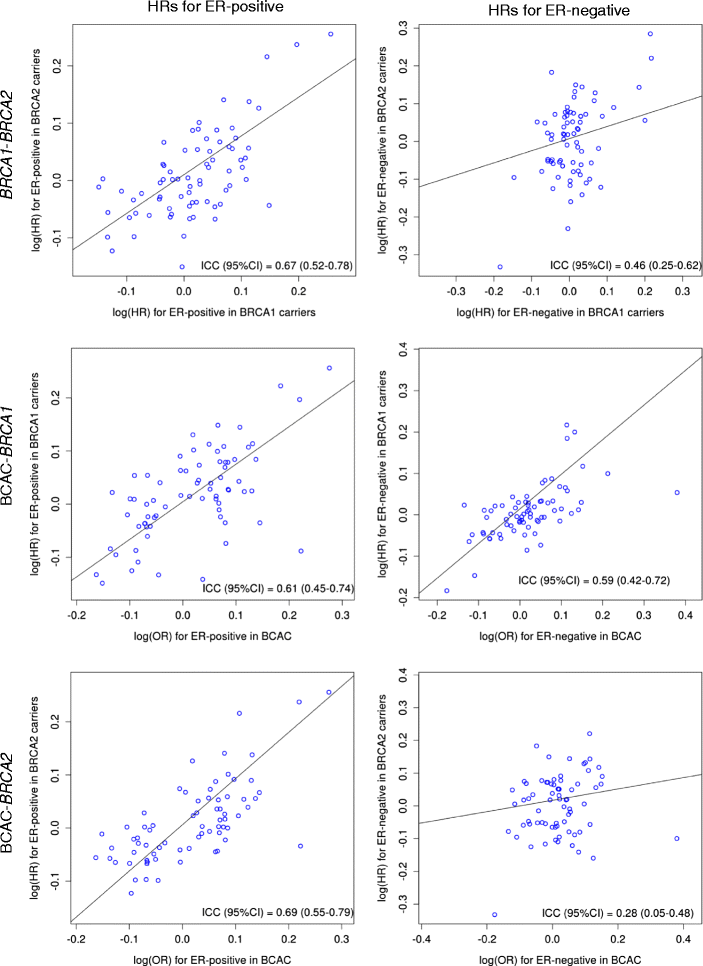

Supplement: Supplementary file 2 — Authors’ original file for figure 1 [file 13058_2014_492_MOESM2_ESM.gif]
